# GO/MNPs–TEA–CuI in water: a green and efficient catalytic system for multicomponent preparation of highly substituted imidazoles and oxazoles

**DOI:** 10.3389/fchem.2025.1732911

**Published:** 2026-02-05

**Authors:** Mohamed Abu Shuheil, Ahmed Aldulaimi, Rekha M. M., Subhashree Ray, Omayma Salim Waleed, C. P. Surya, Renu Sharma, Vatsal Jain

**Affiliations:** 1 Faculty of Allied Medical Sciences, Hourani Center for Applied Scientific Research, Al-Ahliyya Amman University, Amman, Jordan; 2 Faculty of Pharmacy, Al-Zahrawi University, Karbala, Iraq; 3 Department of Chemistry and Biochemistry, School of Sciences, Jain (Deemed to be University), Bangalore, Karnataka, India; 4 Department of Biochemistry, IMS and SUM Hospital, Siksha “O” Anusandhan, Bhubaneswar, Odisha, India; 5 Department of Anesthesia Techniques, Health and Medical Techniques College, Alnoor University, Mosul, Iraq; 6 Department of Chemistry, Sathyabama Institute of Science and Technology, Chennai, Tamil Nadu, India; 7 Department of Chemistry, University Institute of Sciences, Chandigarh University, Mohali, Punjab, India; 8 Centre for Research Impact and Outcome, Chitkara University Institute of Engineering and Technology, Chitkara University, Rajpura, Punjab, India

**Keywords:** aqueous medium, GO/MNPs–TEA–CuI nanocatalyst, imidazoles, oxazoles, recyclable catalyst

## Abstract

A sustainable and highly efficient catalytic protocol has been developed employing graphene oxide-supported magnetic nanoparticles functionalized with triethanolamine and copper(I) iodide (GO/MNPs–TEA–CuI) for the synthesis of functionalized imidazole and oxazole derivatives. This magnetically recoverable catalyst promotes multicomponent coupling reactions between aryl aldehydes, aryl nitriles, and terminal alkynes in aqueous media under reflux conditions, emphasizing its environmentally benign nature. The system efficiently affords a broad scope of products in excellent yields (77%–99%) within short reaction times (15–80 min for imidazoles and 1–8 h for oxazoles). The catalyst exhibits outstanding activity, attributed to the synergistic interaction between the GO surface, magnetic core, and Cu(I) active centers, resulting in high TON and TOF values. Moreover, it tolerates diverse functional groups, including electron-donating, electron-withdrawing, and heteroaromatic substituents, enabling versatility in substrate design. The catalyst can be conveniently separated using an external magnet and reused for several consecutive cycles without appreciable loss of efficiency. Overall, this green and recyclable catalytic system offers an economical and scalable approach for constructing nitrogen- and oxygen-containing heterocycles, providing a promising route for sustainable synthesis in pharmaceutical and fine chemical industries.

## Graphical Abstract

**Figure:**
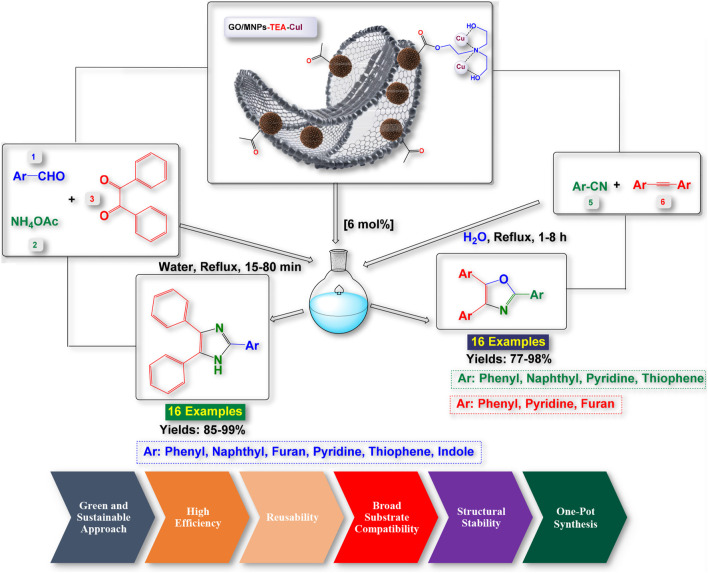

## 1 Introduction

Heterocyclic compounds, particularly imidazoles and oxazoles, hold a central place in the realms of medicinal, agricultural, and materials chemistry, owing to their remarkable biological activities and structural diversity (Al-Mutabagani et al., 2021; Basouti and Saadatjoo, 2017; Mahmudzadeh and Saberi, 2024; Naderi, 2023). Imidazoles, with their characteristic five-membered rings, frequently grace the molecular architecture of natural products and pharmaceutical agents, showcasing a spectrum of properties such as antifungal, anti-inflammatory, and anticancer effects (Chundawat et al., 2015; Moghadasi and Noory Fajer, 2023; Noory Fajer et al., 2023; Prashanth et al., 2022). On the other hand, oxazoles, distinguished by their unique fused structures, are essential building blocks in a range of bioactive molecules, enzyme inhibitors, and innovative organic materials (Hou and Kazemi, 2024; Kandula et al., 2021; Safari and Zarnegar, 2013; Shroff and Panu, 2023). Given their profound significance in various scientific fields, the quest for efficient, cost-effective, and environmentally sustainable methods to construct these heterocycles has become a paramount focus within the realm of synthetic organic chemistry (Aghaseyedkarimi and Naeimi, 2025; Alalaq et al., 2025; Shroff and Panu, 2023).

Traditional synthesis methods often unfold as intricate narratives, requiring laborious multistep procedures, and employing harsh reaction conditions that may involve toxic solvents or stoichiometric quantities of heavy metal reagents (Amaya-García et al., 2024; Kumaraswamy and Gangadhar, 2019). Such practices inherently prompt concerns regarding financial viability, environmental repercussions, and scalability (Liu et al., 2021; Sanchez-Mendoza and Lago, 2024). In response to these pressing challenges, multicomponent reactions (MCRs) have emerged as formidable allies in the synthetic toolkit. These elegant reactions facilitate the assembly of complex molecules in a single, streamlined step, boasting high atom economy and reduced waste generation (Singh and Abraham, 2023; Xu et al., 2013). However, a common thread runs through many reported MCRs: reliance on non-recyclable catalysts, the use of volatile organic solvents, and energy-intensive protocols, underscoring the necessity for continued innovation and refinement in synthetic methodologies (Chatterjee et al., 2016; Kazemi, 2020).

Graphene oxide-supported magnetic nanoparticles (GO/MNPs) offer several key advantages that make them highly attractive in modern catalysis (Sadighian et al., 2021). The high surface area of graphene oxide provides ample active sites for catalytic species, enhancing reaction rates and efficiency (Kholghi Eshkalak et al., 2018; Sheikh et al., 2022). Additionally, the presence of oxygen-containing functional groups (such as hydroxyl, epoxy, and carboxyl groups) on the GO surface allows for strong anchoring of metal ions or organic ligands, preventing catalyst leaching and improving stability (Hojati et al., 2020; Zhu et al., 2010). The magnetic core (typically Fe_3_O_4_ or other magnetic oxides) allows for easy recovery of the catalyst using an external magnet, simplifying the workup process and minimizing waste (Pawar et al., 2024). This magnetic feature not only facilitates recyclability but also reduces the need for filtration or centrifugation, making the process more sustainable and cost-effective. Moreover, GO/MNPs catalysts exhibit excellent chemical, thermal, and mechanical stability, enabling their use under a broad range of reaction conditions, including aqueous media, high temperatures, or in the presence of various functional groups (Mirshafiee and Rezaei, 2023; Naderi, 2023). Their tunable surface properties allow for the immobilization of diverse catalytic species, including transition metals, organocatalysts, and enzymes, enabling wide applicability across different types of reactions (Daraie et al., 2019). These features make GO/MNPs catalysts ideal for promoting green, efficient, and selective transformations in organic synthesis, aligning well with the principles of sustainable and environmentally friendly chemistry (Chen et al., 2019).

Among various catalytic systems, copper-based catalysts have demonstrated significant utility in promoting heterocycle-forming reactions due to their affordability, availability, and compatibility with nitrogen-containing substrates. In recent years, nanostructured-copper catalysts, particularly those supported on graphene oxide (GO) and magnetic nanoparticles (MNPs), have attracted attention due to their high surface area, ease of functionalization, and convenient magnetic separation. When combined with GO and MNP supports, copper(I) iodide (CuI) can offer enhanced dispersion, improved reactivity, and easy recyclability.

In this context, we report an efficient, green, and recyclable catalytic protocol using a GO/MNPs–TEA–CuI nanocomposite for the synthesis of triaryl imidazoles and trisubstituted oxazoles under aqueous reflux conditions. The catalyst system leverages the synergistic effects of graphene oxide, magnetic nanoparticles, and CuI, with triethanolamine serving as ligand. The reactions proceed smoothly in water, obviating the need for organic solvents and external additives, and afford the desired heterocycles in excellent yields (up to 99%) with short reaction times and broad substrate scope.

## 2 Experimental

### 2.1 Materials and methods

All the essential chemicals for this project were sourced from reputable suppliers, Fisher and Merck. We employed solvents and reagents from Sigma-Aldrich, Fluka, or Merck, directly utilizing them without any further purification. To closely monitor our reactions, we employed the thin-layer chromatography (TLC) technique, while purifying our compounds was achieved through meticulous column chromatography using high-quality Merck silica gel, graded between 230 and 400 mesh. Furthermore, we captured detailed 1H NMR and 13C NMR spectra, utilizing the sophisticated Bruker DRX-400 spectrophotometer operating at frequencies of 400 MHz and 100 MHz, respectively, to gain deeper insights into our molecular structures.

### 2.2 Preparation of GO/MNPs nanocomposite

A 1-g quantity of graphene oxide (GO) was dispersed in 50 mL of distilled, deionized water and sonicated for 30 min, converting its surface carboxylic acid groups into carboxylate anions. Separately, 1 mmol (1.99 g) of iron (II) chloride tetrahydrate and 2 mmol (5.40 g) of iron (III) chloride hexahydrate were dissolved in 25 mL of distilled water, with their crystalline forms dissolving completely. This iron-rich solution was then gradually added to the GO suspension at room temperature, with vigorous stirring while a gentle nitrogen flow created a dynamic reaction atmosphere. Ammonia solution was slowly introduced, raising the pH to a brisk 12 and promoting the formation of magnetite nanoparticles (Fe_3_O_4_). As the reaction proceeded and the mixture reached 80 °C, stirring continued steadily for 5 h, allowing complex interactions to unfold. After cooling to room temperature, the suspension was rinsed with deionized water and subjected to magnetic separation to collect the product, which was then gently dried at 60 °C for 12 h. The result was a batch of magnetite nanoparticles embedded in or associated with the graphene oxide matrix, poised for further exploration of their properties and potential applications.

### 2.3 Preparation of GO/MNPs-COCl nanocomposite

In a meticulously controlled laboratory environment, 2 g of GO/MNPs were subjected to vigorous reflux with 60 mL of thionyl chloride (SOCl_2_) at 100 °C for 8 h, under a protective nitrogen blanket to prevent unwanted reactions. This crucial step activates and functionalizes the surface carboxyl groups of the graphene oxide. During the process, the carbonyl (C=O) group of the carboxylic acid reacts with the chloride ions released by thionyl chloride, forming acyl chlorides. To ensure product purity, anhydrous THF was used to thoroughly remove residual thionyl chloride from the newly formed acyl-functionalized graphene oxide, now designated GO/MNPs-COCl.

Following activation, the GO/MNPs-COCl nanomaterial underwent extensive purification, including three successive washes with anhydrous toluene to ensure complete elimination of impurities. After this cleansing sequence, the resulting solid GO/MNPs-COCl was efficiently separated using an external magnet. The final step involved gentle vacuum-drying at room temperature, a careful method chosen to preserve the functionalized material’s integrity and properties.

### 2.4 Preparation of GO/MNPs-TEA nanocomposite

To construct the GO/MNPs-TEA nanocomposite, we began by combining 1 g of GO/MNPs-COCl with 2.5 g of triethanolamine in 50 mL of dimethylformamide (DMF) as the solvent, which promoted smooth dispersion. The mixture was then sonicated for 30 min, allowing high-frequency sound waves to agitate the components and promote thorough uniform integration. Following sonication, the blend was heated to 100 °C and stirred for 8 h to facilitate adequate bonding and enhance the nanocomposite’s structure. After the reaction, the GO/MNPs-TEA nanocomposite was isolated using a magnet to ensure efficient extraction. To ensure purity, the solid was washed three times with methylene chloride, using an external magnet to aid collection after each wash. Finally, the material was dried under vacuum, yielding a dry and stable GO/MNPs-TEA nanocomposite ready for future applications and research.

### 2.5 Preparation of GO/MNPs-TEA-CuI nanocomposite

To prepare the innovative GO/MNPs-TEA-CuI nanocomposite, we began by adding 3 mmol of CuI to a well-blended suspension containing 1 g of ultrasonically dispersed graphene oxide and magnetic nanoparticles modified with triethanolamine (GO/MNPs-TEA), all in 50 mL of DMF. The mixture was heated to 100 °C and maintained at this temperature for 5 h to promote interactions and transformations among the components, ultimately yielding the iron oxide-coated silica nanocatalyst designated GO/MNPs-TEA-CuI. After synthesis, magnetic separation was used to recover the nanocomposite from the solution, and the material was thoroughly washed with hot water and ethanol to remove residual impurities. Finally, to ensure stability and optimal form, the product was gently dried at 60 °C, yielding a nanocomposite ready for further applications.

### 2.6 General method for the synthesis of triaryl imidazole derivatives catalyzed by GO/MNPs-TEA-CuI

In a 20 mL flask, a carefully measured combination was assembled: 0.3 mmol of aryl aldehydes, 0.6 mmol of ammonium acetate, and 0.3 mmol of benzyl derivatives. To this reactive mixture, the GO/MNPs-TEA-CuI catalyst (6% molar scale) was added, initiating the transformation. The mixture was then gently stirred in 3 mL of water and brought to reflux, allowing the reaction to proceed for the designated intervals described in Table 2. The reaction was monitored closely by TLC to capture each stage of the transformation.

Upon completion, the catalyst was readily separated with a magnet, and the reaction mixture was cooled to room temperature. The cooled solution was diluted with 20 mL of dichloromethane (CH_2_Cl_2_) and filtered to remove any particulates, ensuring a clean organic phase. Solvent evaporation yielded the target product, which was subsequently isolated by silica gel flash chromatography using a eluent composed of hexane and ethyl acetate. The resulting 2,4,5-triaryl imidazole products were obtained as well-characterized compounds, with spectroscopic data aligning with established literature values. Additionally, comprehensive NMR data for the benzimidazole derivatives are documented in the Supplementary Material, providing a thorough record of the synthetic outcomes.

### 2.7 General method for the synthesis of triaryl oxazole derivatives catalyzed by GO/MNPs-TEA-CuI

In a 20 mL round-bottom flask, a reaction was initiated as an aryl nitrile (0.5 mmol) and an alkyne (0.2 mmol) joined forces in the presence of the GO/MNPs-TEA-CuI catalyst (6% mol), all dissolved in a 4 mL of water. The mixture was brought to a gentle reflux, bubbling softly as the transformation progressed, with TLC diligently monitoring the reaction’s course. Upon completion, the catalyst was readily removed with a stirring bar, and the mixture was allowed to cool to room temperature. Aqueous Na_2_CO_3_ was added, the organic layer was separated and the aqueous phase was subjected. The organic phase was carefully separated, and the aqueous phase was subjected to a second extraction with ethyl acetate (2 × 10 mL) to recover any remaining product. Purification was achieved by silica gel column chromatography using a carefully chosen solvent system of petroleum ether and ethyl acetate (50:1), yielding a clean separation. The process yielded the desired 2,4,5-triaryl oxazole products, which were obtained as well-characterized compounds with spectroscopic data aligning with established literature values. Additionally, detailed NMR data for the benzimidazole derivatives are documented in the Supplementary Material, offering a comprehensive record of the successful outcomes from this synthetic endeavor.

## 3 Results and discussions

**SCHEME 1:**
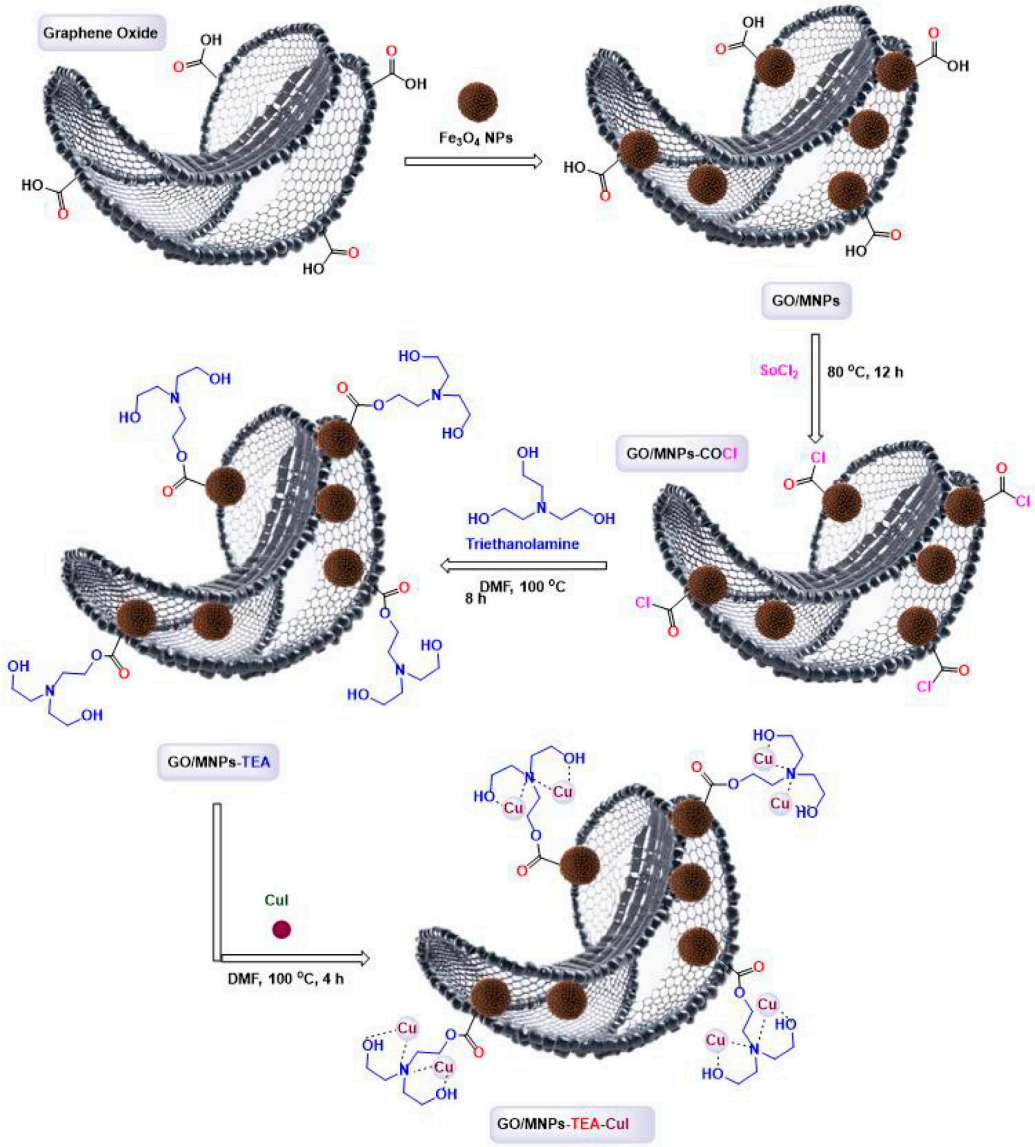
Details of the structural preparation of GO/MNPs-TEA-CuI nanocomposite.

The structural preparation of the GO/MNPs–TEA–CuI nanocomposite involves a multi-step chemical functionalization process (Scheme 1). Initially, graphene oxide (GO), rich in oxygen-containing functional groups such as hydroxyl, epoxy, and carboxyl groups, is decorated with Fe_3_O_4_ magnetic nanoparticles (MNPs) to produce GO/MNPs. These magnetic particles enable easy separation and recovery of the material. Next, the carboxyl groups on GO are converted to more reactive acyl chloride groups using thionyl chloride (SOCl_2_), resulting in GO/MNPs–COCl. This intermediate is then reacted with triethanolamine (TEA) in DMF at elevated temperature, where the hydroxyl groups of TEA react with the acyl chlorides, forming covalent linkages and yielding GO/MNPs–TEA. In the final step, the GO/MNPs–TEA complex is treated with copper(I) iodide (CuI) in DMF. The nitrogen and hydroxyl functionalities of TEA act as coordinating ligands for Cu(I) ions, anchoring them firmly onto the surface of the GO composite. This results in the formation of the final GO/MNPs–TEA–CuI nanocomposite, which features magnetic properties, a stable carbon framework, and catalytically active Cu(I) sites.

**SCHEME 2:**
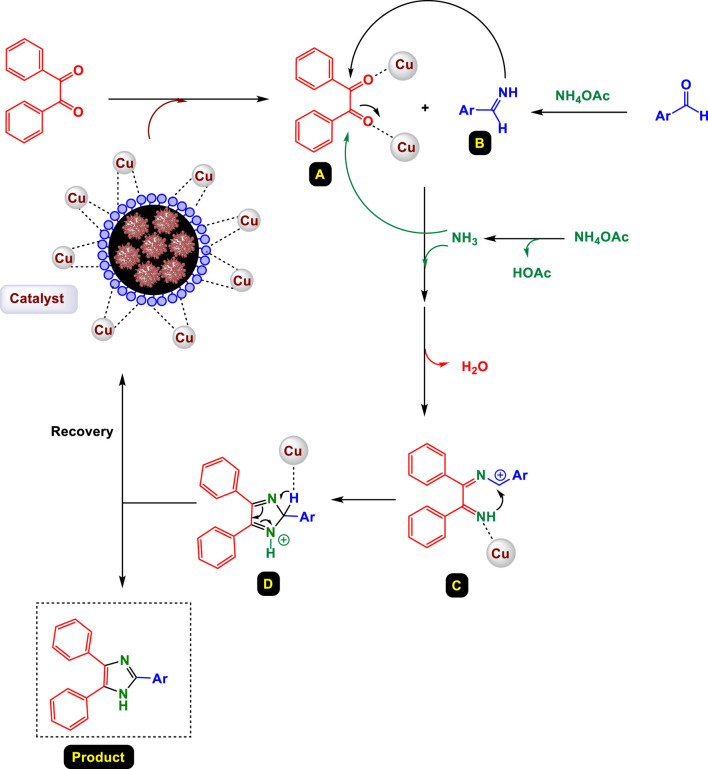
Suggested mechanism for the synthesis of highly substituted imidazoles catalyzed by GO/MNPs-TEA-CuI nanocomposite.

**SCHEME 3:**
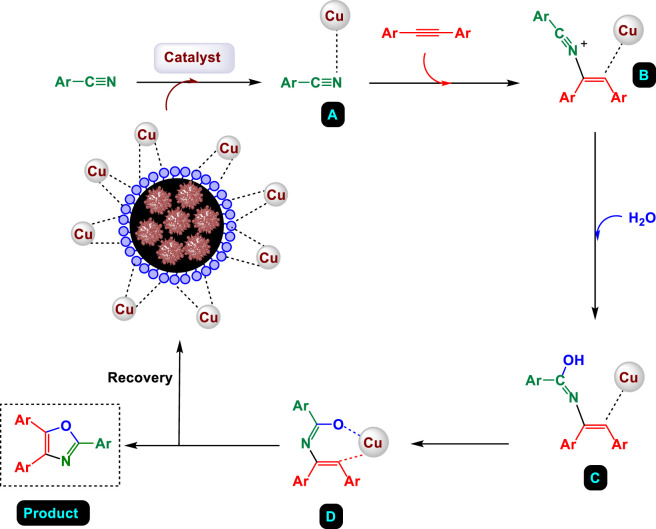
Suggested mechanism for the synthesis of highly substituted oxazoles catalyzed by GO/MNPs-TEA-CuI nanocomposite.

### 3.1 Characterization of GO/MNPs-TEA-CuI nanocomposite

**FIGURE 1:**
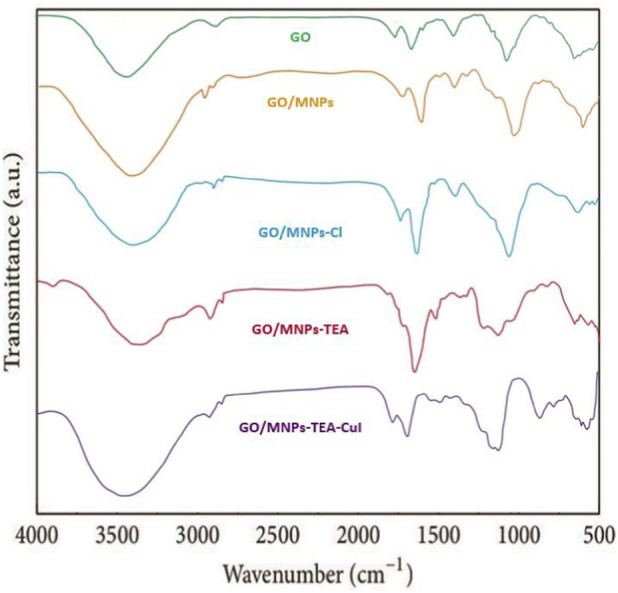
FT-IR spectra of Graphene Oxide (GO), GO/MNPs, GO/MNPs-Cl, GO/MNPs-TEA, and GO/MNPs-TEA-CuI nanocomposite.

The FT-IR spectra shown in Figure 1 provide insight into the stepwise chemical modifications of graphene oxide (GO) through the formation of the GO/MNPs–TEA–CuI nanocomposite. Each spectrum reflects the vibrational modes of functional groups present at various stages of synthesis, confirming successful chemical transformations. The FT-IR spectrum of Graphene Oxide (GO) displays characteristic absorption bands indicative of oxygen-containing functional groups. A broad peak around 3400 cm^-1^ corresponds to O–H stretching vibrations from hydroxyl and carboxylic acid groups. The band at 1720 cm^-1^ is attributed to C=O stretching of carbonyl groups, while peaks near 1620 cm^-1^ correspond to C=C stretching from the aromatic ring. Additional peaks at 1220–1050 cm^-1^ are assigned to C–O stretching vibrations from epoxy and alkoxy groups. In the GO/MNPs spectrum, there is a noticeable change compared to GO. The broad O–H peak persists, but the intensity of the C=O band around 1720 cm^-1^ slightly decreases, suggesting interactions between GO’s oxygen groups and the Fe_3_O_4_ nanoparticles. A new band appears around 580–600 cm^-1^, which is characteristic of Fe–O stretching, confirming the successful deposition of magnetic nanoparticles onto the GO surface.

The spectrum of GO/MNPs–Cl (after treatment with thionyl chloride) reveals a significant reduction in the O–H band, and the emergence of new bands near 1800–1780 cm^-1^, indicating the formation of acid chloride (COCl) functionalities. The suppression of hydroxyl and carboxylic peaks confirms successful chlorination, replacing these groups with more reactive acyl chlorides. Upon reaction with triethanolamine (TEA), the GO/MNPs–TEA spectrum shows a reappearance and broadening of the O–H stretching band (∼3400 cm^-1^), along with new peaks around 1100–1250 cm^-1^, corresponding to C–N and C–O stretching from the TEA structure. These features confirm the successful attachment of TEA to the GO framework via ester or amide linkages, as well as the presence of its hydroxyl arms. Finally, the spectrum of GO/MNPs–TEA–CuI shows further changes. There is a slight shift and broadening of the bands in the region of 1000–1250 cm^-1^, suggesting coordination between Cu(I) ions and the nitrogen or oxygen atoms of the TEA ligand. Additionally, new weaker peaks may appear below 600 cm^-1^, typically associated with metal–ligand (Cu–O and Cu–N) interactions, supporting the successful incorporation of Cu(I) into the nanocomposite. Overall, the FT-IR data clearly illustrate the sequential chemical transformations and functionalizations throughout the synthesis process.

**FIGURE 2:**
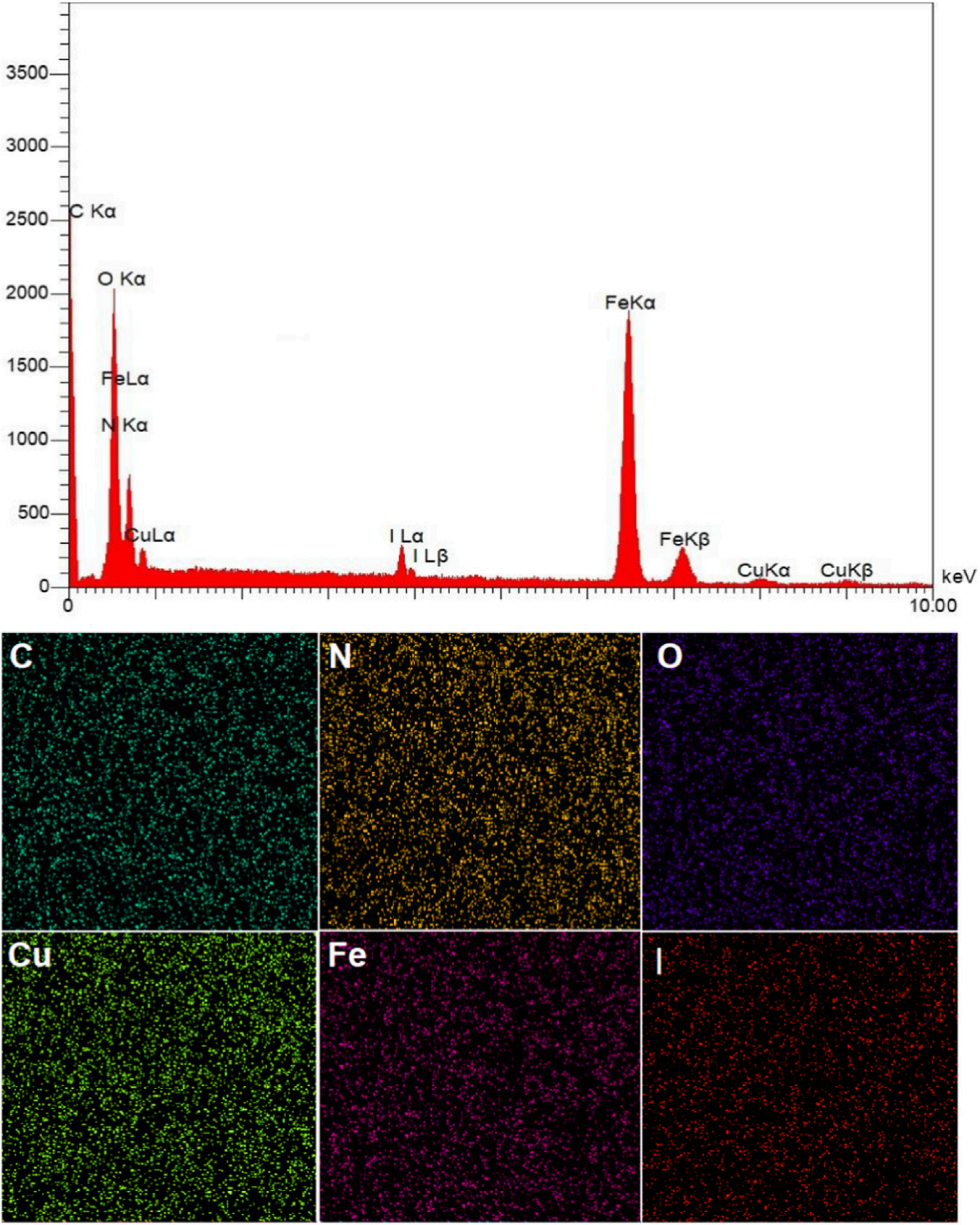
EDX and Elemental mapping analysis of GO/MNPs-TEA-CuI nanocomposite.

Figure 2 showcases the EDX spectrum and the elemental mapping analysis of the GO/MNPs–TEA–CuI nanocomposite, confirming the successful integration of several key elements into the material. The EDX spectrum reveals significant peaks for carbon (C), oxygen (O), nitrogen (N), iron (Fe), copper (Cu), and iodine (I). The presence of carbon and oxygen aligns with the expected characteristics of the graphene oxide structure, while the detection of iron signals indicates the successful embedding of Fe3O4 magnetic nanoparticles. The nitrogen signal stems from triethanolamine (TEA), which contains these nitrogen atoms. Notably, the peaks for copper and iodine highlight the successful coordination of copper(I) iodide with the TEA-functionalized GO/MNPs, confirming that the nanocomposite formation process has been completed.

Moreover, the elemental mapping images further reinforce the uniform distribution of each element across the composite’s surface. The consistent presence of carbon, nitrogen, and oxygen reflects the distribution of the GO backbone and TEA functional groups, while iron mapping shows an even incorporation of Fe_3_O_4_ nanoparticles, enhancing the composite’s magnetic properties. The consistent distribution of copper and iodine reinforces their stable coordination to the TEA ligands. This uniformity is essential for maintaining the structural integrity of the GO/MNPs–TEA–CuI nanocomposite, which is critical for its functionality in catalytic and environmental applications. Additionally, the ICP-OES analysis indicates a noteworthy copper concentration of 1.39 × 10^−3^ mol/g, confirming a substantial incorporation of copper within the composite structure, which is vital for its catalytic efficiency.

**FIGURE 3:**
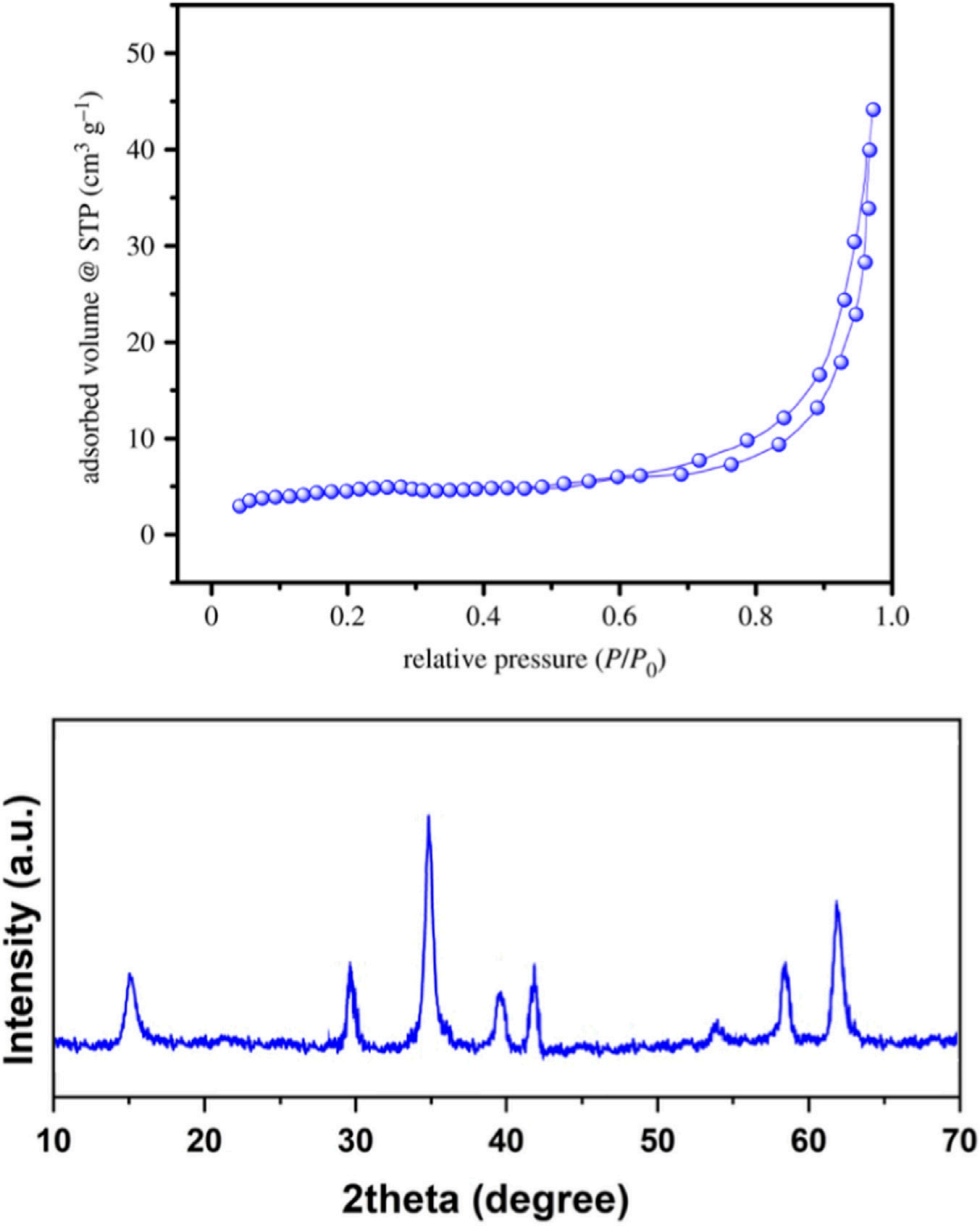
BET and XRD patterns of GO/MNPs-TEA-CuI nanocomposite.

The Brunauer–Emmett–Teller (BET) analysis illustrated in Figure 3 (top graph) showcases the nitrogen adsorption–desorption isotherm for the GO/MNPs-TEA-CuI nanocomposite. The observed isotherm distinctly follows a Type IV curve, characterized by a pronounced hysteresis loop, a clear indication that the material possesses mesoporous properties. Upon a detailed investigation of the pore size distribution, it was determined that the average pore diameter of the catalyst measures 27.48 nm. Furthermore, the pore volume analysis revealed a significant reduction in this parameter, decreasing from an initial value of 11.298 cm^3^/g to 6.743 cm^3^/g. This reduction in pore volume might be attributed to alterations in the catalyst’s structural integrity or the occurrence of pore blockage phenomena. The porosity of the material is of paramount importance for catalytic applications, as it not only enhances the overall surface area but also promotes better accessibility of reactants to the active sites embedded within the composite structure.

The XRD pattern of the GO/MNPs-TEA-CuI nanocomposite reveals distinct peaks corresponding to each of its components, confirming successful synthesis (Figure 3). Notably, the broad peak typically observed for graphene oxide (GO) around 2θ ≈ 10°, which indicates the (001) plane, is either absent or considerably reduced. This significant change suggests that exfoliation and chemical modification of the GO layers have taken place, transforming their structure. Furthermore, the XRD analysis reveals characteristic peaks associated with Fe_3_O_4_ magnetic nanoparticles, appearing at specific angles of 2θ ≈ 30.1°, 35.5°, 43.2°, 53.5°, 57.1°, and 62.7°. These peaks correspond to the (220), (311), (400), (422), (511), and (440) crystallographic planes, respectively, which confirm the presence of crystalline magnetite within the composite. In addition to the GO and Fe_3_O_4_ signatures, distinct peaks observed at approximately 25.5°, 29.6°, 42.3°, 50.1°, and 61.3° further indicate the successful formation of the cubic-phase CuI. This peak positioning substantiates that copper(I) iodide has been effectively integrated and anchored within the nanocomposite structure. These results collectively confirm the structural integrity and integration of GO, Fe_3_O_4_, and CuI in the nanocomposite.

**FIGURE 4:**
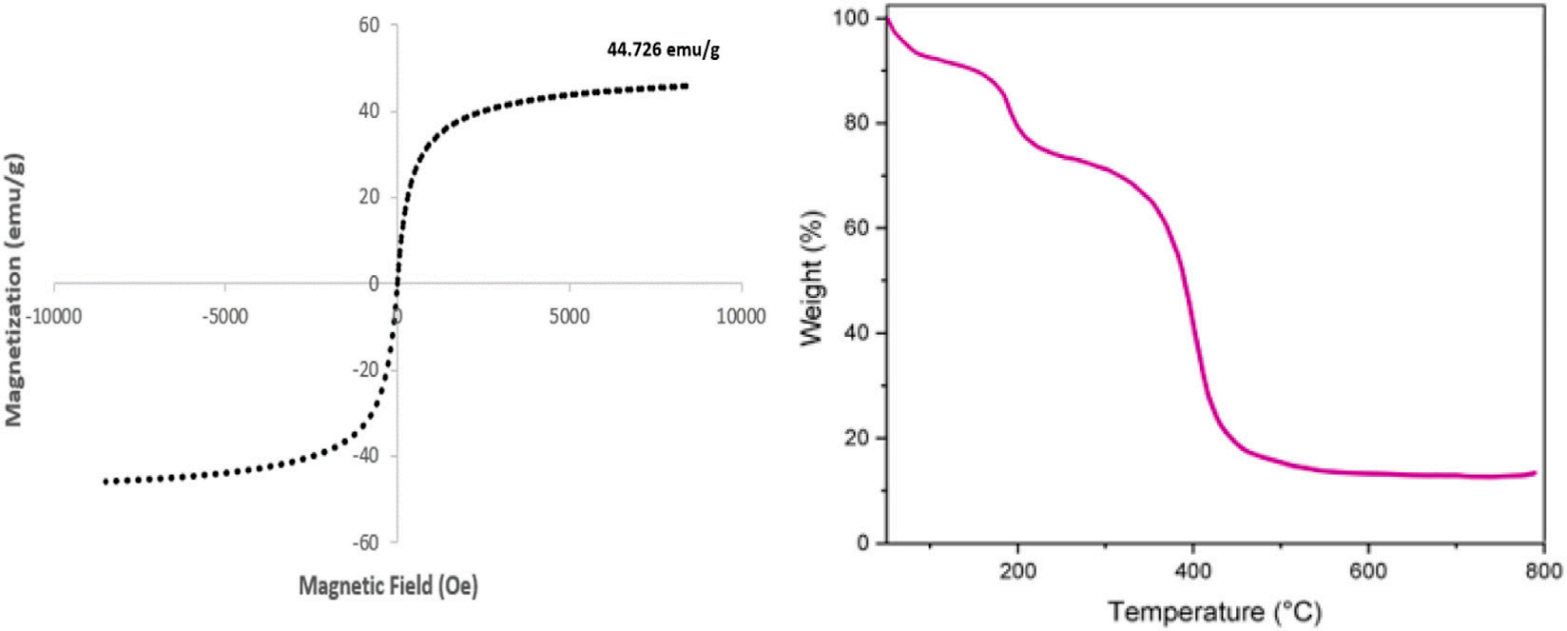
VSM and TGA spectra of GO/MNPs-TEA-CuI nanocomposite.

The Vibrating Sample Magnetometry (VSM) spectrum presented in the left graph of Figure 4 reveals the magnetic properties of the GO/MNPs-TEA-CuI nanocomposite, showcasing a clear hysteresis loop that indicates superparamagnetic behavior with minimal coercivity and remanence. This behavior is complemented by a remarkable saturation magnetization value of 44.726 emu/g, signifying a strong magnetic response primarily due to the presence of Fe_3_O_4_ magnetic nanoparticles (MNPs) within the graphene oxide (GO) matrix. Such impressive magnetic characteristics make the nanocomposite particularly advantageous for applications like magnetic separation, where the nanocatalyst can be easily and efficiently retrieved from reaction mixtures using an external magnet, thereby enhancing the effectiveness and simplicity of the recovery process.

The Thermogravimetric Analysis (TGA) spectrum, depicted in the right graph of Figure 4, offers valuable insights into the thermal stability and composition of the nanocomposite. The TGA curve reveals three distinct stages of weight loss that correspond to different thermal events. Initially, between 100 °C and 150 °C, the curve indicates the loss of adsorbed water and moisture trapped within the material. As the temperature rises to between 200 °C and 400 °C, we see a significant decline likely due to the thermal degradation of organic compounds, particularly triethanolamine (TEA), and the breakdown of oxygenated functional groups on graphene oxide (GO). The most significant weight loss occurs beyond 400 °C, marking the decomposition of the GO framework and the degradation of any remaining organic components. Notably, the residual weight observed beyond 600 °C points to the presence of thermally stable inorganic materials, like Fe_3_O_4_ and CuI, highlighting the composite’s hybrid nature. Overall, these TGA results emphasize the excellent thermal stability of the nanocomposite, positioning it as a promising candidate for high-temperature applications.

**FIGURE 5:**
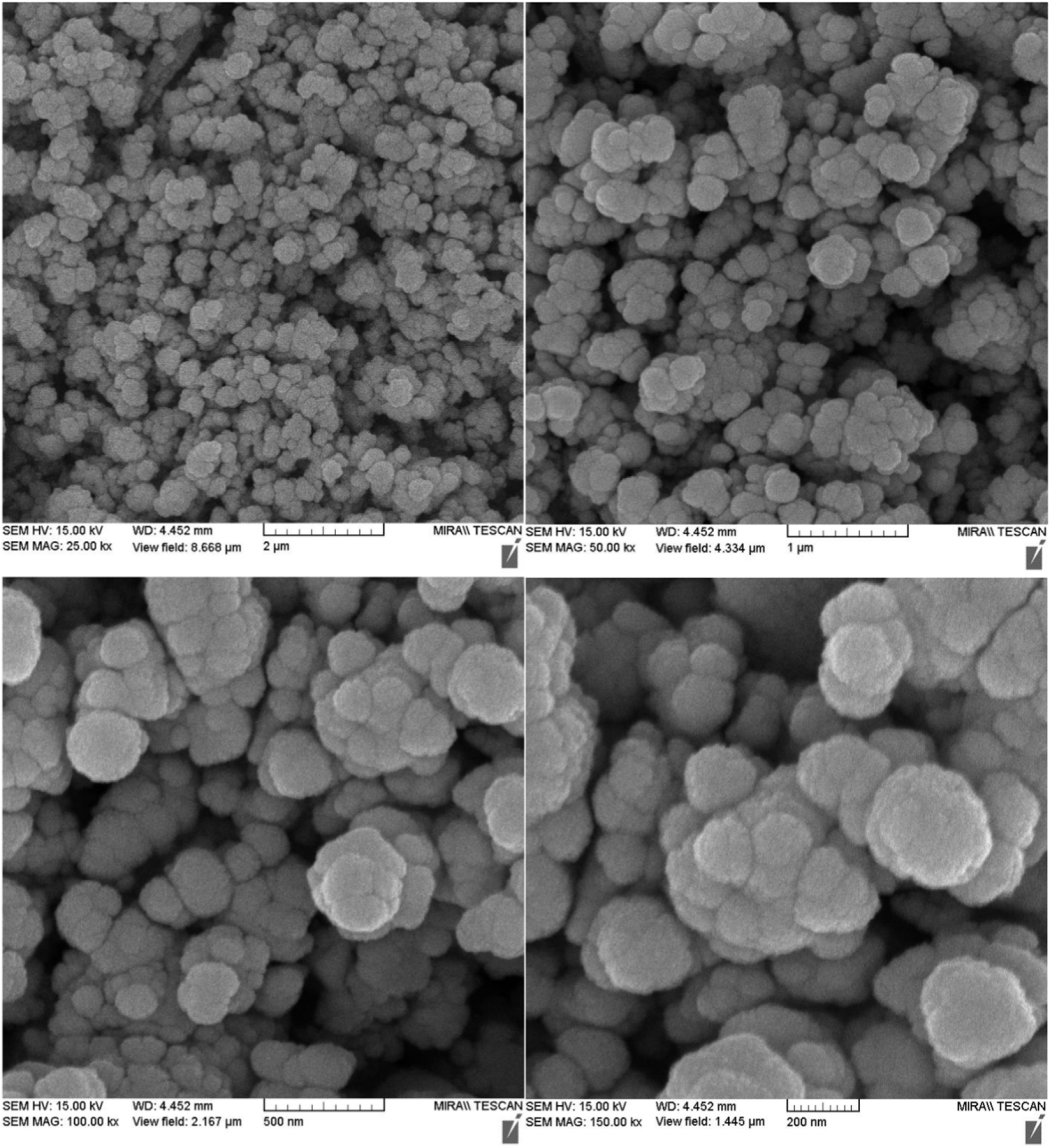
SEM images (2µm, 1µm, 500 nm, 200 nm) of GO/MNPs-TEA-CuI catalyst.

In Figure 5, the SEM images taken at different magnifications (2 μm, 1 μm, 500 nm, and 200 nm) provide comprehensive surface morphology information about the GO/MNPs-TEA-CuI catalyst. At lower magnifications (2 μm and 1 µm), the catalyst exhibits a highly aggregated, clustered morphology composed of spherical and semi-spherical nanoparticles forming dense, coral-like structures. These particles appear to be evenly distributed with some degree of agglomeration, which is typical for magnetic nanoparticles (MNPs) and indicates strong interparticle interactions. As the magnification increases (500 nm and 200 nm), the surface texture becomes clearer, revealing individual nanoparticles with smooth surfaces and relatively uniform sizes. The higher-resolution images suggest that the catalyst has a porous, high-surface-area structure, which is advantageous for catalytic reactions due to the increased number of accessible active sites. The observed particle size distribution and surface roughness imply that the functionalization with TEA and loading of CuI were successful and well-integrated into the GO matrix.

**FIGURE 6:**
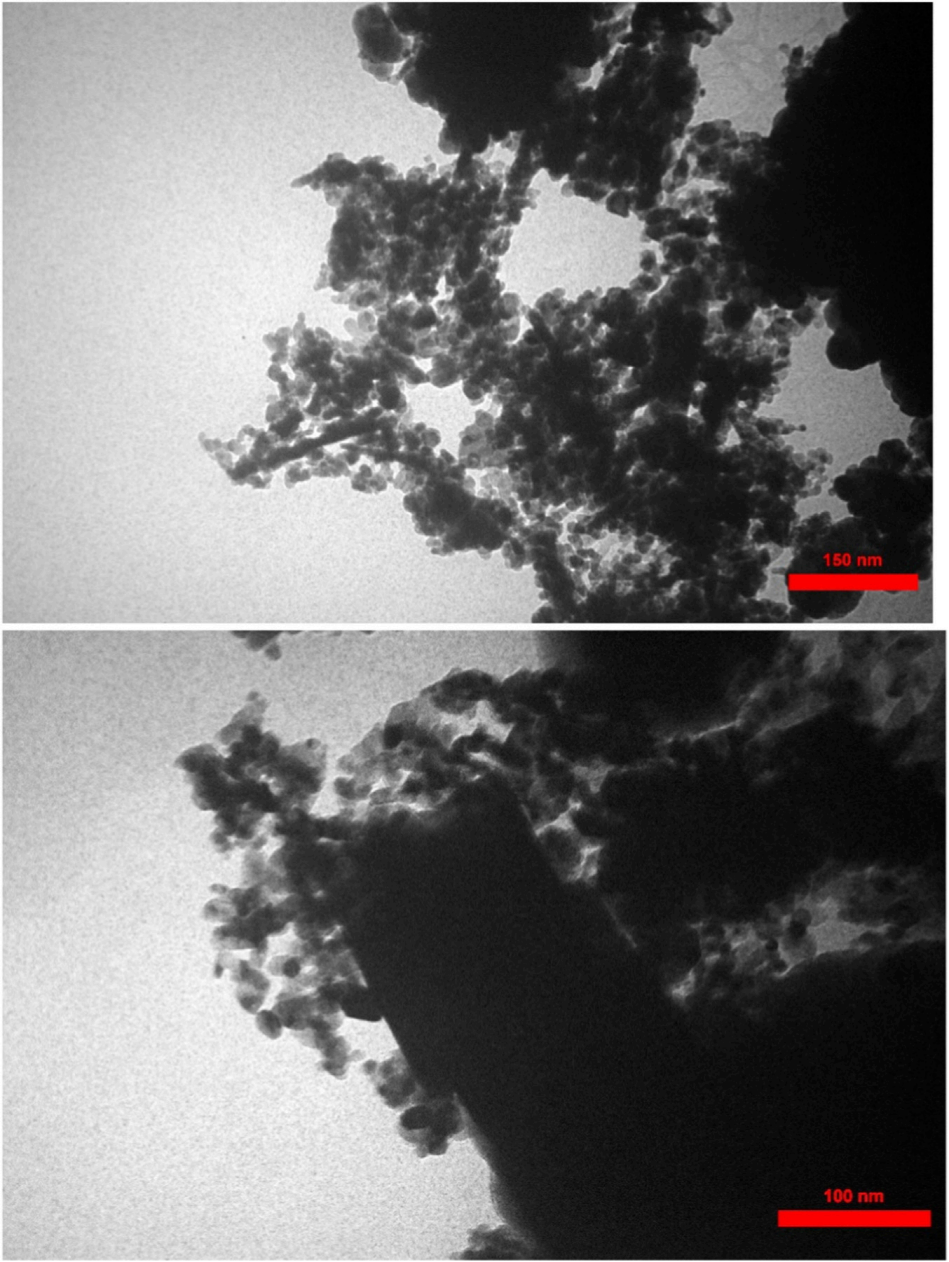
TEM images (150 nm, 100 nm) of GO/MNPs-TEA-CuI catalyst.

In Figure 6, the TEM images (150 nm and 100 nm scales) provide detailed internal and interfacial structural information of the GO/MNPs-TEA-CuI catalyst. These images show a distinct contrast between the darker, electron-dense regions—representing the MNPs/CuI nanoparticles—and the lighter, semi-transparent areas, which correspond to the thin layers of graphene oxide (GO). The TEM at 150 nm reveals a loosely packed network of nanoparticles spread over a wrinkled GO sheet, confirming the successful anchoring of the nanoparticles on the GO support. At 100 nm, more detail emerges: individual nanoparticles are clearly visible with well-defined boundaries, suggesting a narrow particle size distribution and minimal agglomeration. The intimate contact between the nanoparticles and GO sheets implies good chemical bonding and stable integration, likely due to the coordination with the TEA linker. This structural configuration enhances the dispersion of the active CuI sites and supports efficient electron transfer, which are critical factors for catalytic performance. Overall, the TEM analysis corroborates the SEM findings and further validates the nanocomposite’s homogeneity and well-organized microstructure.

### 3.2 Catalytic investigation in synthesis of highly substituted imidazoles and oxazoles

Table 1 presents a detailed investigation into the optimization of reaction conditions for the synthesis of product 4a via a one-pot multicomponent reaction involving 4-chlorobenzaldehyde, benzyl, and ammonium acetate, catalyzed by a functionalized copper catalyst, GO/MNPs–TEA–Cu(I). The key goal was to identify the ideal catalyst loading, solvent, reaction time, and temperature to achieve the highest yield of the desired product.

**TABLE 1:** Optimization conditions for model reaction (product 4a).

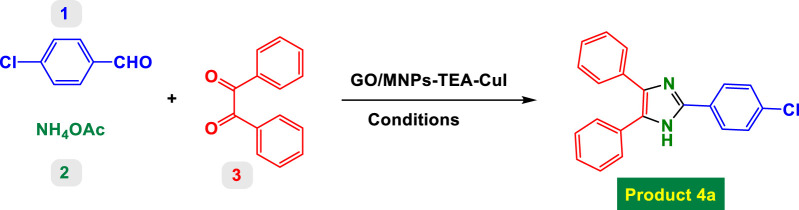				

Entry	Catalyst [mol%]	Solvent (^o^C)	Time (min)	Yield (%)^a^
1	No	EtOH (Reflux)	600	NR
2	GO [3 mol%]	EtOH (Reflux)	300	NR
3	GO/MNPs [3 mol%]	EtOH (Reflux)	300	Trace
4	GO/MNPs–TEA [3 mol%]	EtOH (Reflux)	300	14%
5	GO/MNPs–TEA–CuI [3 mol%]	EtOH (Reflux)	90	70%
6	GO/MNPs–TEA–CuI [3 mol%]	PEG (100 °C)	60	75%
7	GO/MNPs–TEA–CuI [3 mol%]	MeCN (Reflux)	60	34%
8	GO/MNPs–TEA–CuI [3 mol%]	Toluene (100 °C)	60	9%
9	GO/MNPs–TEA–CuI [3 mol%]	DMSO (100 °C)	60	39%
10	GO/MNPs–TEA–CuI [3 mol%]	Water (Reflux)	40	85%
11	GO/MNPs–TEA–CuI [3 mol%]	ChCl-Urea (100 °C)	40	81%
12	GO/MNPs–TEA–CuI [3 mol%]	[BMIM]PF_6_	40	79%
13	GO/MNPs–TEA–CuI [3 mol%]	1,4-Dioxane	40	16%
14	GO/MNPs–TEA–CuI [3 mol%]	Water (90 °C)	40	80%
15	GO/MNPs–TEA–CuI [4 mol%]	Water (Reflux)	30	93%
16	GO/MNPs–TEA–CuI [5 mol%]	Water (Reflux)	20	97%
17	GO/MNPs–TEA–CuI [6 mol%]	Water (Reflux)	15	99%
18	GO/MNPs–TEA–CuI [7 mol%]	Water (Reflux)	15	99%
19	GO/MNPs–TEA–CuI [8 mol%]	Water (Reflux)	15	99%
20	GO/MNPs–TEA–CuI [10 mol%]	Water (Reflux)	15	99%

^a^ Yields referred to isolated products.

#### 3.2.1 Catalyst screening

The initial entries (1–5) in Table 1 show the influence of different catalytic systems. Entry 1, conducted without any catalyst, resulted in no reaction (NR) even after 600 min, confirming the necessity of a catalyst. Entries 2 and 3 used GO and GO/MNPs respectively, both showing negligible activity. Entry 4 used GO/MNPs–TEA and gave a low yield of 14%. A significant improvement was observed in entry 5 with GO/MNPs–TEA–Cu(I), where a 70% yield was achieved in just 90 min in refluxing ethanol. This clearly demonstrates the essential role of the copper-functionalized nanocatalyst in driving the reaction efficiently.

#### 3.2.2 Solvent effects

Solvent screening (entries 6–14) revealed that solvent polarity and water-compatibility were critical to reaction efficiency. Water as a solvent (entry 10) gave a notably high yield of 85% in just 40 min, indicating its suitability both from a green chemistry and reactivity standpoint. Other solvents like PEG (entry 6) and ChCl-Urea (entry 11) also gave decent yields (75% and 81%, respectively), but water remained superior due to simplicity, cost, and environmental impact. Notably, non-polar solvents like toluene (entry 8) and even common organic solvents like MeCN (entry 7) and DMSO (entry 9) gave significantly lower yields (9%–39%).

#### 3.2.3 Temperature optimization

Water as a solvent was further examined under varying temperatures (entries 10 and 14). While entry 10 (reflux) gave 85% yield in 40 min, entry 14 at 90 °C slightly improved the yield to 80%, indicating that a modest increase in temperature might enhance efficiency. Still, the most efficient condition in terms of time and yield was achieved under reflux with water.

#### 3.2.4 Catalyst loading optimization

Entries 15–20 investigate the effect of catalyst loading using GO/MNPs–TEA–Cu(I) in water under reflux conditions. Increasing the catalyst loading from 4 mol% (entry 15) to 6 mol% (entry 17) steadily improved the yield, peaking at 99% in just 15 min with 6 mol%. Beyond this, increasing the loading to 7–10 mol% did not further improve the yield, indicating that 6 mol% was the optimal loading for maximum efficiency and atom economy.

The optimal conditions for synthesizing product 4a were identified as: using 6 mol% of GO/MNPs–TEA–Cu(I) catalyst in water under reflux conditions for 15 min, yielding 99%. This condition is not only highly efficient but also aligns with green chemistry principles due to the use of water as solvent and a recyclable heterogeneous catalyst. This table systematically showcases how each parameter—catalyst, solvent, temperature, and loading—critically influences the reaction’s efficiency and provides a robust methodology for further synthetic applications.

The catalytic system based on GO/MNPs–TEA–CuI (graphene oxide/magnetic nanoparticles–triethylamine–copper(I) iodide) presents a highly efficient and environmentally benign platform for the synthesis of highly substituted imidazoles (Table 2) and oxazoles (Table 3). This nanocomposite catalyst operates in aqueous medium under reflux conditions, which aligns with green chemistry principles by reducing the use of hazardous organic solvents. It offers notable advantages including high yields (up to 99%), short reaction times, good substrate compatibility, and recyclability of the catalyst, as implied by consistent performance across diverse entries. The scope covered in both tables includes a broad range of electron-donating and electron-withdrawing substituents, various heteroaryl systems, and the tolerance for sterically demanding substrates.

**TABLE 2:** Scope of GO/MNPs-TEA-CuI catalyst in the preparation of highly substituted imidazoles^a^.

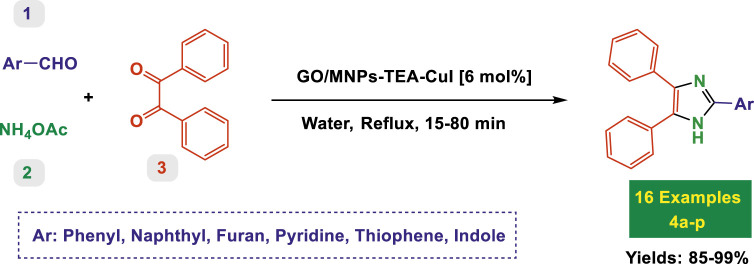						

Entry	Imidazole products	Time (min)	Yield (%)^b^	TON	TOF (min)^−1^	M.P [Ref]
1	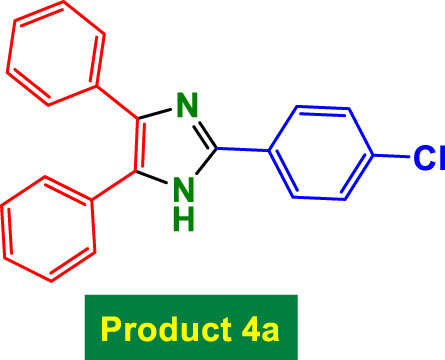	15	99%	16.5	1.10	269–271 °C (Yang et al., 2021)
2	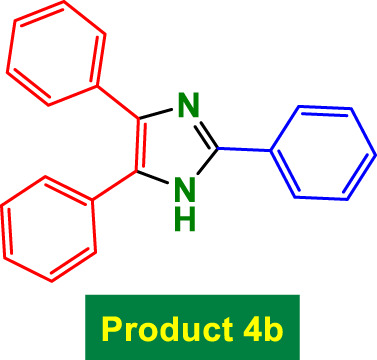	25	98%	16.33	0.65	269–271 °C (Aghaseyedkarimi and Naeimi, 2025)
2	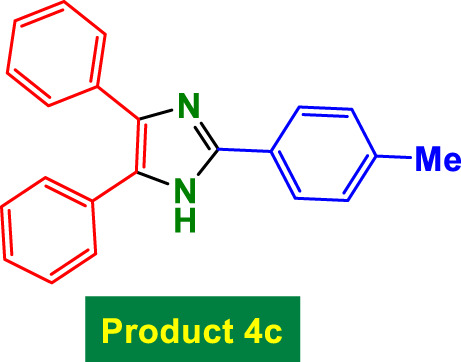	20	97%	16.17	0.81	231–233 °C (Aghaseyedkarimi and Naeimi, 2025)
4	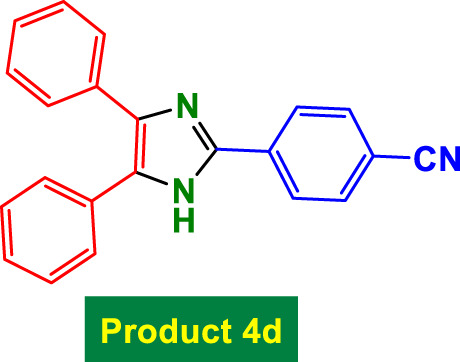	75	87%	14.50	0.19	230–232 °C (Khalifeh and Niknam, 2020)
5	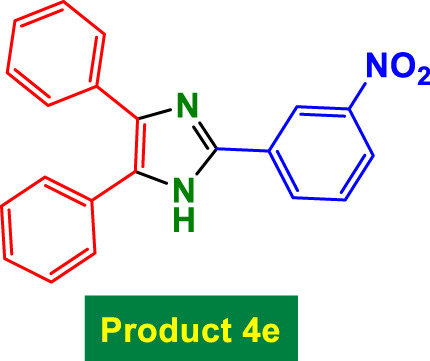	80	85%	14.17	0.18	313–315 °C (Alalaq et al., 2025)
6	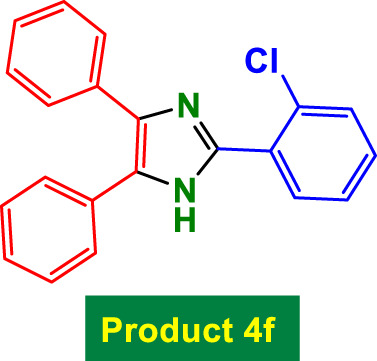	25	92%	15.33	0.61	187–189 °C (Allahvirdinesbat et al., 2017)
7	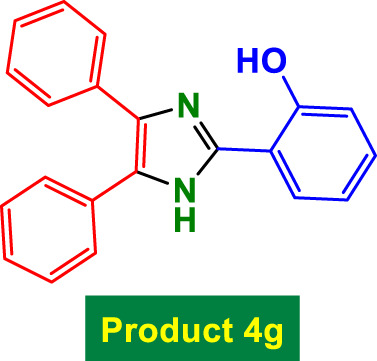	35	90%	15.00	0.43	203–205 °C (Zhang et al., 2025)
8	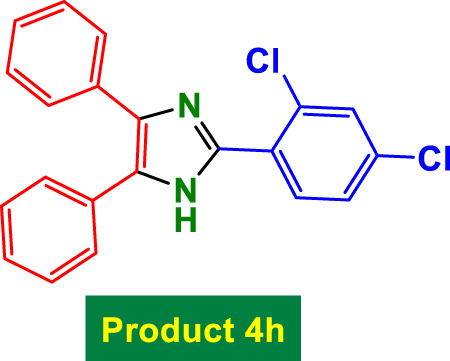	30	93%	15.50	0.52	170–172 °C (Naeimi and Aghaseyedkarimi, 2015)
9	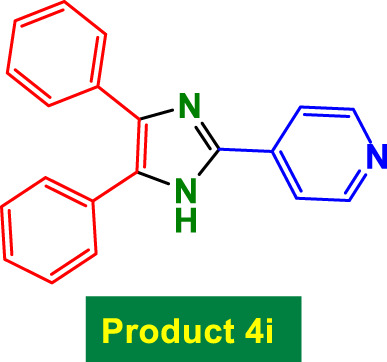	25	94%	15.67	0.63	231–233 °C (Selvakumar et al., 2017)
10	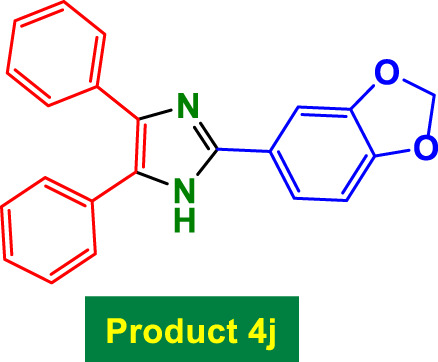	20	96%	16.00	0.80	255–257 °C (Higuera et al., 2019)
11	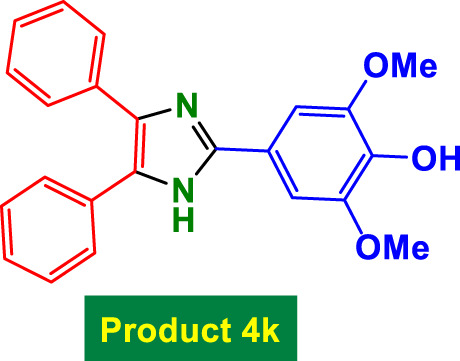	45	91%	15.17	0.34	281–283 °C (Gurjar et al., 2018)
12	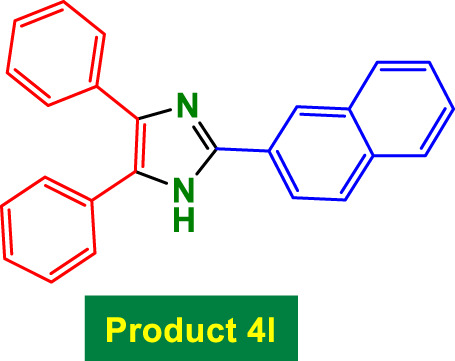	20	98%	16.33	0.82	278–280 °C (Naidoo and Jeena, 2020)
13	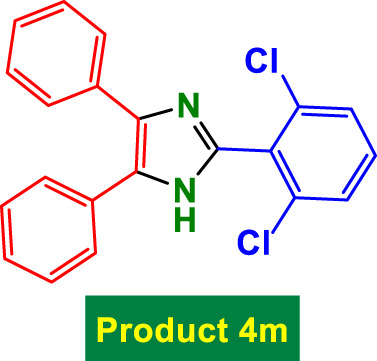	40	91%	15.17	0.38	234–236 °C (Asressu et al., 2021)
14	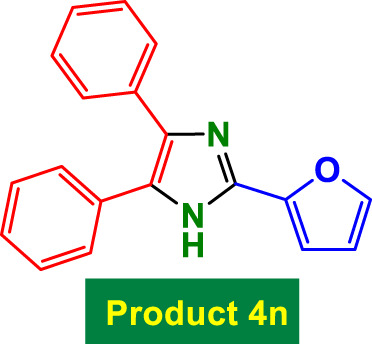	40	96%	16.00	0.4	232–235 °C (Bahrami et al., 2016)
15	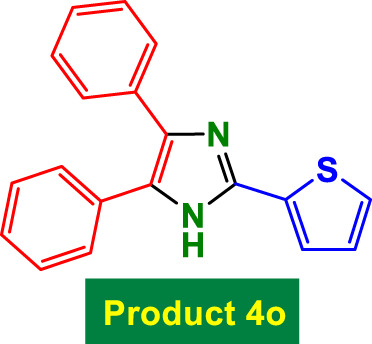	20	98%	16.33	0.82	256–258 °C (Nukala et al., 2023)
16	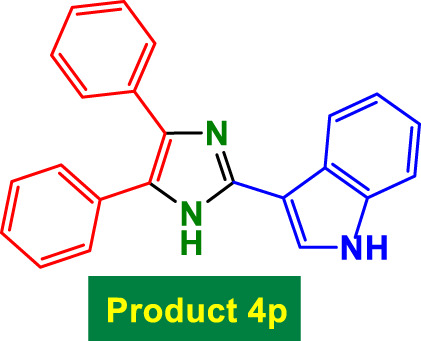	35	97%	16.17	0.46	306–308 °C (Jopale et al., 2024)
17	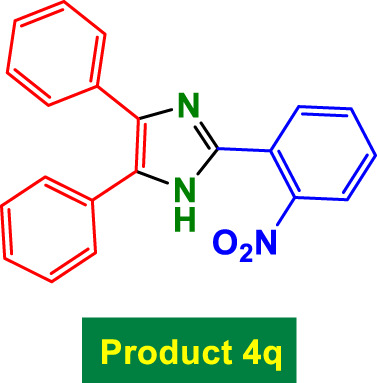	80	85%	14.17	0.18	235–237 °C [40]
18	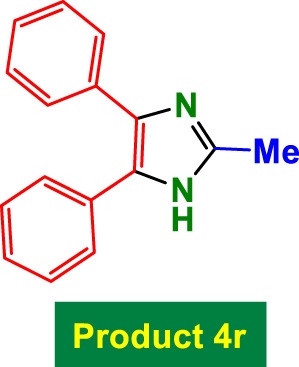	70	88%	14.66	0.21	240–242 °C [41]
19	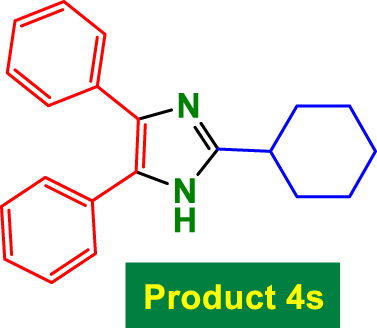	85	87%	14.50	0.17	241–243 °C [42]

^a^ Reaction conditions: Aryl aldehydes (0.3 mmol), NH_4_OAc (0.6 mmol), Benzyl (0.3 mmol), GO/MNPs-TEA-CuI, catalyst [6 mol%] in water (3 mL) at reflux temperature.

^b^ Yields referred to isolated products.

**TABLE 3:** Scope of GO/MNPs-TEA-CuI catalyst in the preparation of highly substituted oxazoles^a^.

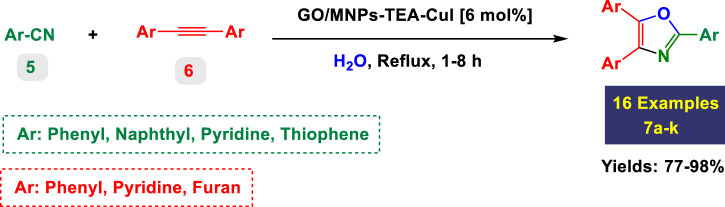						

Entry	Oxazole products	Time (h)	Yield (%)^b^	TON	TOF (h)^−1^	M.P [Ref]
1	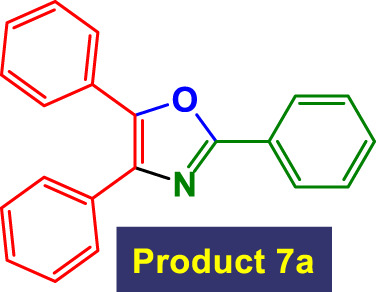	1	98%	16.33	16.33	111–113 °C (Hsieh et al., 2018)
2	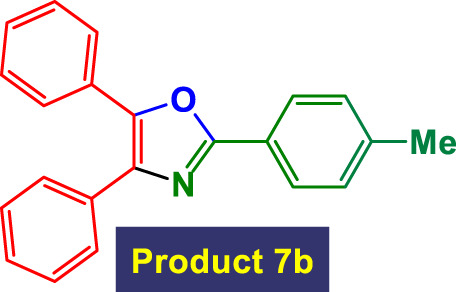	1	97%	16.17	16.17	126–128 °C (Mazibuko and Jeena, 2023)
3	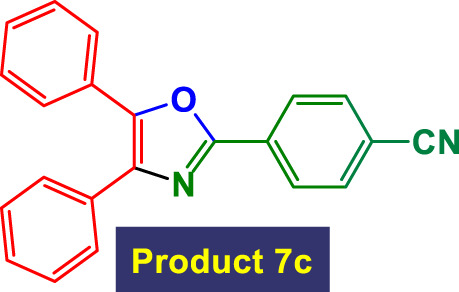	6	84%	14.00	2.33	129–131 °C (Khazipov et al., 2022)
4	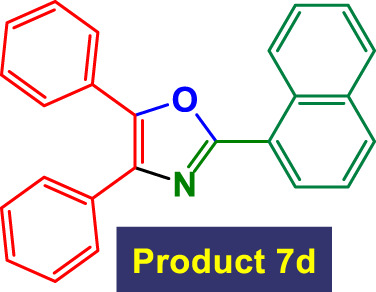	1.5	96%	16.00	10.67	125–127 °C (Sarkar and Mukhopadhyay, 2015)
5	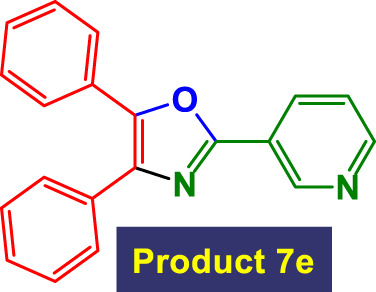	2	97%	16.17	8.08	105–107 °C (Dai et al., 2020)
6	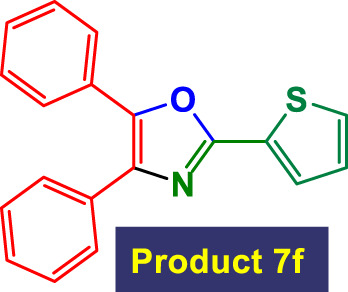	3	93%	15.50	5.17	113–115 °C (Kandula et al., 2021)
7	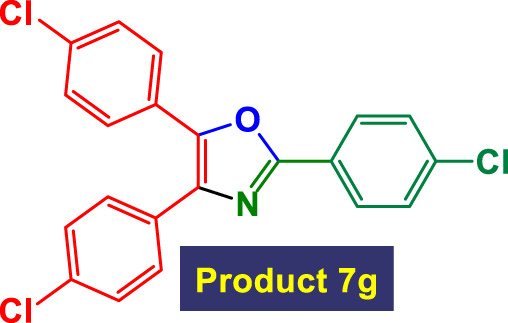	2	94%	15.67	7.83	144–146 °C (Amaya-García et al., 2024)
8	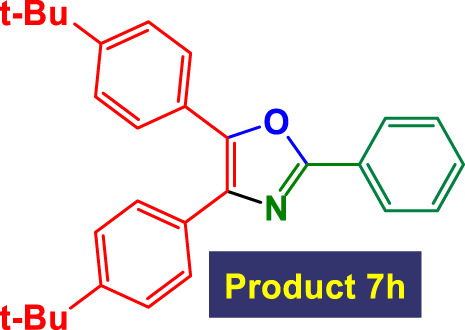	8	77%	12.83	1.60	151–153 °C (Yamamoto et al., 2017)
9	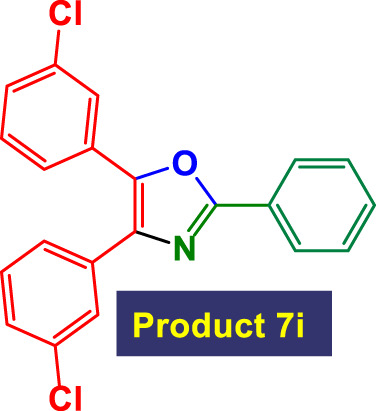	2	84%	14.00	7.00	116–118 °C (Hu et al., 2013)
10	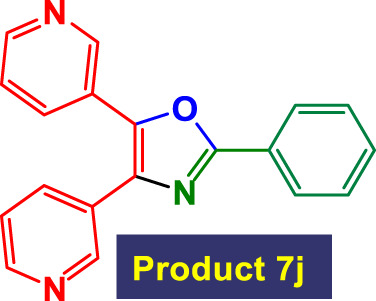	3.5	86%	14.33	4.10	131–133 °C (Jiang, 2025)
11	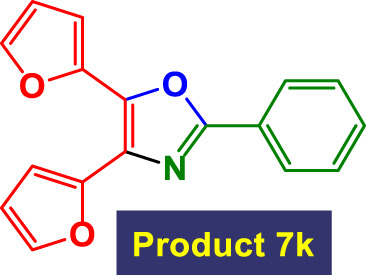	5	91%	15.17	3.03	102–104 °C (Wei and Yuan, 2023)
12	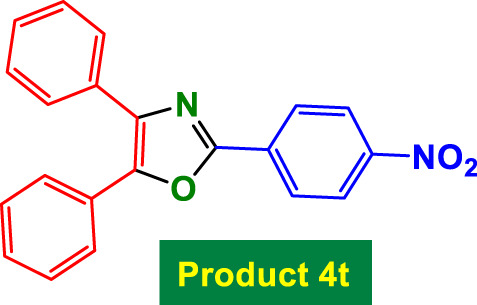	75	86%	14.33	0.19	143–145 °C [43]

^a^ Reaction conditions: Aryl nitrile (0.5 mmol), Alkyne (0.2 mmol) GO/MNPs-TEA-CuI, catalyst [6 mol%] in water (4 mL) at reflux temperature.

^b^ Yields referred to isolated products.

#### 3.2.5 Substituted imidazoles—substrate scope and functional group effects

In Table 2, the condensation of aryl aldehydes with ammonium acetate and benzyl in the presence of 6 mol% GO/MNPs–TEA–CuI leads to the formation of triaryl imidazoles. The substrate scope includes aldehydes bearing a variety of substituents, including electron-withdrawing groups such as–Cl (Entry 1, 4a), –NO_2_ (Entry 5, 4e), –CN (Entry 4, 4d), and electron-donating groups such as–Me (Entry 3, 4c) and–OH (Entry 7, 4g). It is observed that electron-withdrawing groups tend to increase the reactivity of the aldehydes, facilitating a higher electrophilicity of the carbonyl group, and thus tend to give high yields in relatively short reaction times (e.g., Entry 1: 15 min, 99%). Conversely, more deactivating or sterically hindered substituents may require longer reaction times (e.g., Entry 5: 80 min for 85%) (Table 2).

The catalyst also tolerates heteroaryl groups (Entries 9–16), including pyridine (4i), furan (4j), thiophene (4k), and indole (4p). These heteroaryl aldehydes generally performed well, with yields >90% in most cases, reflecting the catalyst’s robustness toward heteroatoms, which might typically coordinate to copper and deactivate other catalysts. Notably, even di-substituted aromatic aldehydes like 2,4-dichlorobenzaldehyde (Entry 8, 4h) performed efficiently, suggesting that steric hindrance does not severely affect the catalytic turnover. Overall, TON values are consistently high (∼15–16), and TOF varies with time but remains efficient.

Scheme 2 illustrates the proposed catalytic mechanism for the synthesis of highly substituted imidazoles using a GO/MNPs-TEA-CuI nanocomposite catalyst. This heterogeneous catalyst combines graphene oxide (GO), magnetic nanoparticles (MNPs), triethanolamine (TEA), and copper(I) iodide (CuI) to provide a highly efficient and recyclable system for promoting the multicomponent reaction. The reaction begins with the activation of benzyl (1,2-diphenylethane-1,2-dione) by coordination with the Cu(I) center on the nanocomposite surface, generating intermediate **A**. This activation facilitates nucleophilic attack and coordination events necessary for subsequent transformations.

Simultaneously, aryl aldehyde and ammonium acetate react to form an *in-situ* imine or arylamine intermediate **B**, likely through a condensation process mediated by NH_4_OAc. The imine **B** then undergoes nucleophilic addition to the activated diketone intermediate **A**, forming a Cu-coordinated intermediate that quickly dehydrates to form the key imidazole precursor. The mechanism proceeds with cyclization and coordination steps leading to intermediate **C**, in which the imidazole ring framework begins to form. Intermediate **C** undergoes further intramolecular rearrangement and proton shifts, assisted by the catalyst surface, forming the protonated imidazole complex **D**. Final deprotonation yields the fully substituted imidazole product. The catalyst is then regenerated through the dissociation of the product from the copper center, allowing for recovery and reuse.

#### 3.2.6 Substituted oxazoles—substrate scope and functional group effects

In Table 3, the catalytic system is used for the synthesis of highly substituted oxazoles from aryl nitriles and terminal alkynes under similar aqueous reflux conditions. The catalyst exhibits strong versatility with respect to both the nitrile and alkyne partners. A wide array of nitriles with substituents including–Me (7b), –Cl (7g), –CN (7c), pyridyl (7e), and furyl/thiophenyl rings (7f) are well tolerated. Electron-rich nitriles (e.g., Entry 2, 7b) and electron-poor nitriles (Entry 3, 7c) both deliver high to excellent yields, although the reaction time is notably longer for substrates with strong electron-withdrawing groups (6 h for 84% in Entry 3), suggesting reduced nucleophilicity of the nitrile nitrogen slows the cyclization process (Table 3).

The system also handles diverse alkynes, including simple aryl alkynes, bulky tert-butyl-substituted alkynes (7h), and heteroaryl alkynes. However, more sterically hindered or less reactive alkynes tend to result in longer reaction times and slightly reduced yields (e.g., Entry 8, 7h, takes 8 h with only 77% yield). This highlights a potential limitation with bulky or less nucleophilic alkynes under these conditions. Importantly, high TON values (up to 16.3) and high TOFs (over 16 h^-1^) in entries with short reaction times (1 h) indicate the catalyst’s exceptional performance, particularly with less hindered and more electron-neutral substrates.

The proposed mechanism for the synthesis of highly substituted oxazoles catalyzed by the GO/MNPs-TEA-CuI nanocomposite involves a multistep process initiated by the activation of aryl nitrile (Ar–C≡N) and arylacetylene (Ar–C≡C–Ar) by the copper catalyst (Scheme 3). In the first step, the copper species coordinates with the nitrile group to form intermediate **A**. Subsequent interaction with the alkyne leads to the formation of a copper-stabilized intermediate **B**. Upon addition of water, hydrolysis occurs to generate intermediate **C**, which contains a hydroxy-imine moiety coordinated to copper. This transformation sets the stage for cyclization, where the hydroxyl group attacks the imine carbon, resulting in the formation of the oxazole ring system and intermediate **D.** In the final step, the highly substituted oxazole product is released from the copper complex, and the GO/MNPs-TEA-CuI catalyst is recovered magnetically for reuse. The nanocomposite catalyst, which combines graphene oxide for high surface area, magnetic nanoparticles for easy recovery, triethanolamine for stabilization, and copper(I) iodide as the active catalytic center, efficiently drives the reaction through C–C and C–N bond formation.

### 3.3 Recycling results

**FIGURE 7:**
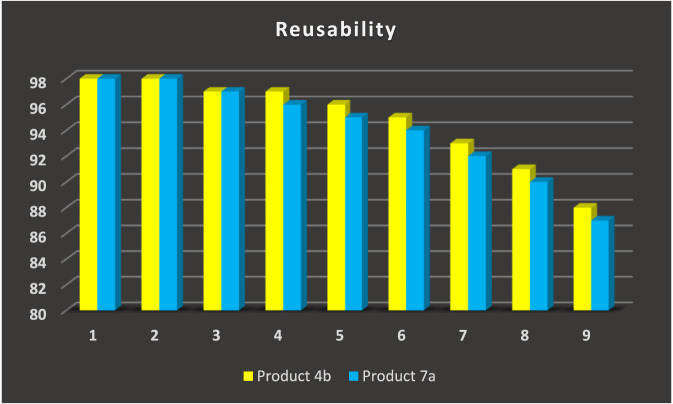
Reusability of GO/MNPs-TEA-CuI catalyst for the preparation of 2,4,5-triphenyl-1*H*-imidazole (product **4b)** and 2,4,5-triphenyloxazole (product **7a)**.

Figure 7 illustrates the reusability of the GO/MNPs-TEA-CuI nanocatalyst in the synthesis of 2,4,5-triphenyl-1H-imidazole (**product 4b**) and 2,4,5-triphenyloxazole (**product 7a**) across nine consecutive reaction cycles. In the first three cycles, the catalyst exhibits excellent performance, maintaining a consistent yield of 98% for both products, indicating high catalytic stability and efficiency. From the fourth to sixth cycle, only a slight reduction is observed, with yields gradually decreasing to 97%–94% for product 4b and 97%–93% for product 7a. This slight decline suggests minor loss in catalytic activity, likely due to minimal structural or surface deactivation. In later cycles (7–9), a more pronounced decrease in yield is evident. Product 4b shows yields of 92%, 90%, and 88%, while product 7a exhibits slightly lower values of 91%, 87%, and 87%, respectively. Despite this gradual reduction, the yields remain within a high and acceptable range, confirming the durability and effectiveness of the GO/MNPs-TEA-CuI catalyst over multiple uses. The ability to retain over 87% yield after nine cycles highlights the catalyst’s excellent reusability, making it a promising and sustainable option for repeated applications in heterocyclic compound synthesis.

**FIGURE 8:**
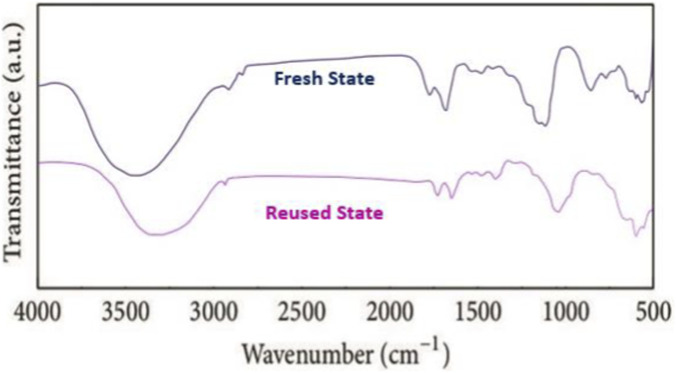
FT-IR spectra of GO/MNPs-TEA-CuI catalyst (in fresh and reused state after 9 times).

**FIGURE 9:**
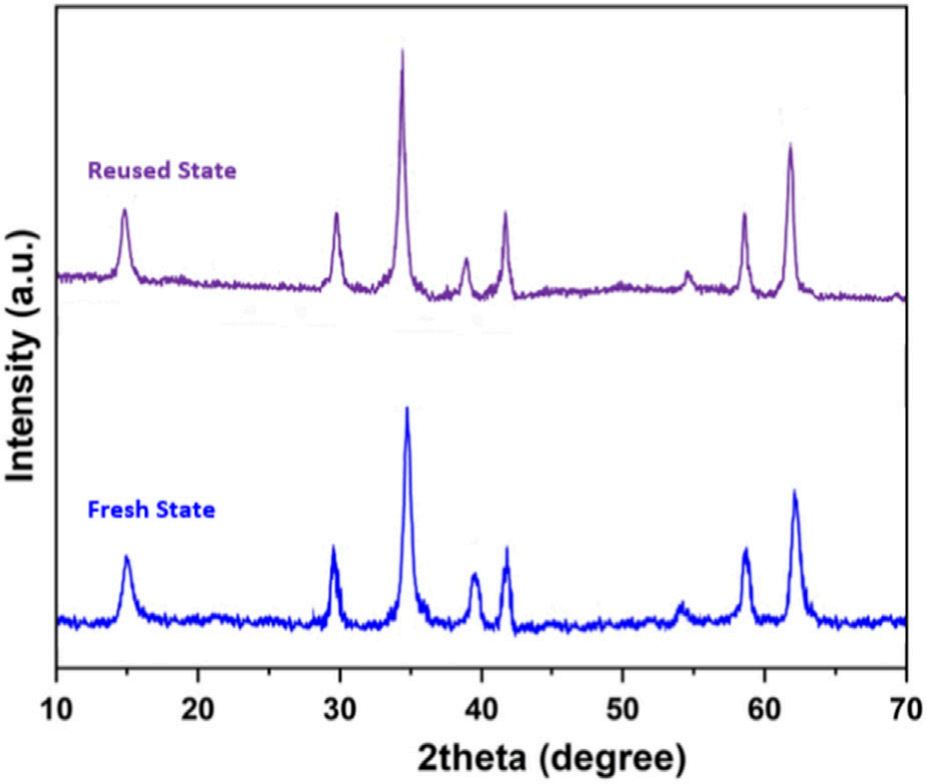
XRD spectra of GO/MNPs-TEA-CuI catalyst (in fresh and reused state after 9 times).

Figures 8, 9 illustrate the FT-IR and XRD spectra of the GO/MNPs-TEA-CuI catalyst in both fresh and reused states, clearly demonstrating the catalyst’s structural integrity after seven reaction cycles. The FT-IR spectra (Figure 8) for the fresh and reused catalysts exhibit nearly identical characteristic peaks with only slight shifts, indicating that the functional groups associated with GO, TEA, and CuI remain largely unchanged after repeated use. Similarly, the XRD patterns (Figure 9) show consistent diffraction peaks between the fresh and reused samples, confirming that the crystalline structure and phase purity of the catalyst are well-preserved. These findings suggest that the catalyst maintains its original morphology, crystallinity, and chemical structure, proving its high thermal and chemical stability during catalytic processes.

**FIGURE 10:**
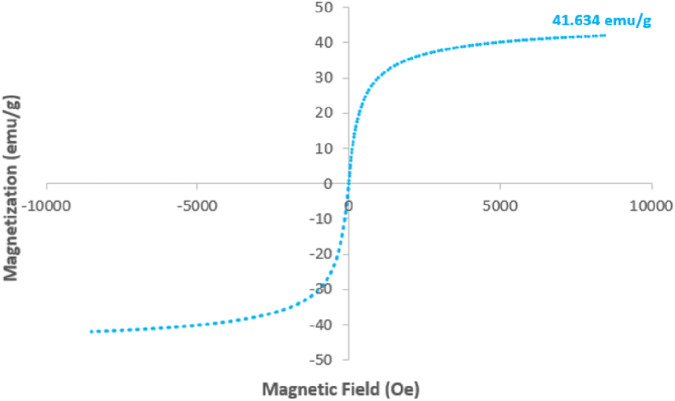
VSM spectra of the reused GO/MNPs-TEA-CuI catalyst after 9 times.

Figure 10 presents the Vibrating Sample Magnetometry (VSM) curve of the reused GO/MNPs-TEA-CuI catalyst, displaying a strong magnetic response with a saturation magnetization value of 41.634 emu/g. Although slightly lower than the fresh state, this high value confirms that the magnetic characteristics are still well-retained, allowing for efficient magnetic recovery and reuse of the catalyst. Additionally, the inductively coupled plasma-optical emission spectrometry (ICP-OES) analysis shows that the copper content in the reused catalyst (1.36 × 10^−3^ mol/g) is only marginally lower than in the fresh catalyst (1.39 × 10^−3^ mol/g), indicating negligible metal leaching during the reaction process. Together, these results validate that the GO/MNPs-TEA-CuI nanocomposite not only offers excellent structural and magnetic stability but also possesses strong durability and reusability for prolonged catalytic applications.

### 3.4 Comparison results

Tables 4, 5 provide a comparative evaluation of the catalytic efficiency of the GO/MNPs-TEA-CuI nanocomposite (Entry 6) against other previously reported catalytic systems in the synthesis of triphenyl imidazole **(4b)** and triphenyl oxazole (**7a**), respectively. The comparison includes key metrics such as reaction time, reaction conditions, reusability, and product yield, offering a comprehensive assessment of the method’s overall performance.

**TABLE 4:** Comparison of the efficiency of this method (Entry 6) with reported methods (Entries 1–5) in the synthesis of triphenyl imidazole (product 4b).

Entry	Catalyst	Conditions	Time (min)	Reusability	Yield (%) [Ref]
1	SBA-15/TFE	Solvent-Free, 120 °C	210	No	92% (Rostamnia and Zabardasti, 2012)
2	L-Proline	MeOH/60 °C	540	No	88% (Samai et al., 2009)
3	Al-MCM-41	Solvent-Free, 120 °C	45	3 Runs	91% (Olyaei et al., 2016)
4	γ-Alumina NPs	EtOH, Reflux	40	4 Runs	92% (Reddy et al., 2015)
5	Benzotriazole	n-BuOH, 80 °C	720	No	88% (Xu et al., 2013)
**6**	**GO/MNPs-TEA-CuI**	**Water, Reflux**	**25**	**8 Runs**	**98% [This work]**

**TABLE 5:** Comparison of the efficiency of this method (Entry 6) with reported methods (Entries 1–5) in the synthesis of triphenyl oxazole (product 7a).

Entry	Catalyst	Conditions	Time (h)	Reusability	Yield (%) [Ref]
1	B(C_6_F5)_3_	Solvent-Free, 120 °C	6	No	78% (Kumaraswamy and Gangadhar, 2019)
2	Pd(OAc)_2_/Ag_2_CO_3_	PFE, 100 °C, air	12	No	77% (Zhang et al., 2017)
3	PhI = O/TfOH	MeCN, 80 °C, O_2_	24	No	69% (Saito et al., 2012)
4	Cu(OTf)2	Solvent-Free, 80 °C	12	No	81% (Prashanth et al., 2022)
5	Fe_3_O_4_@AMNA-CuBr	PEG, 100 °C, O_2_	5	8 Runs	98% (Mahmood Saeed et al., 2023)
**6**	**GO/MNPs-TEA-CuI**	**Water, Reflux**	**1**	**8 Runs**	**98% [This work]**

#### 3.4.1 Comparison for triphenyl imidazole synthesis

In Table 4, the GO/MNPs-TEA-CuI catalyst (**Entry 6**) stands out as the most effective method for the synthesis of triphenyl imidazole. It achieves a remarkably high yield of 98% in only 25 min under mild aqueous reflux conditions, which are environmentally benign compared to other entries that often require organic solvents or high temperatures. Additionally, this catalyst exhibits excellent reusability, maintaining activity over 8 cycles, a key advantage in terms of sustainability and cost-efficiency (Table 4).

In contrast, other reported methods show notable limitations. For instance, entries using SBA-15/TFE (**Entry 1**) and L-Proline (**Entry 2**) involve significantly longer reaction times of 210 and 540 min, respectively, with no reusability, despite giving yields of around 88%–92%. Even the relatively faster catalysts, such as γ-Alumina NPs (**Entry 4**) and Al-MCM-41 (**Entry 3**), require organic solvents (EtOH or solvent-free at 120 °C) and are limited to 3–4 reuse cycles. Moreover, the benzotriazole-catalyzed method (**Entry 5**) needs 720 min for a moderate 88% yield and offers no recyclability. Overall, **Entry 6** in Table 4 demonstrates superior catalytic efficiency, faster kinetics, higher reusability, and the highest yield, establishing it as an advanced and green alternative for triphenyl imidazole synthesis.

#### 3.4.2 Comparison for triphenyl oxazole synthesis

Similarly, in Table 5, the GO/MNPs-TEA-CuI nanocomposite (**Entry 6**) again outperforms all other reported catalysts in the synthesis of triphenyl oxazole. It completes the reaction in just 1 h under water reflux, yielding 98% of product **7a** with 8-cycle reusability—a clear indication of excellent catalytic performance and stability (Table 5).

Compared to this, most of the other catalysts require significantly longer reaction times. For instance, B(C_6_F_5_)_3_ (**Entry 1**) and Cu(OTf)_2_ (**Entry 4**) need 6–12 h under solvent-free or 80 °C–120 °C conditions. Catalysts like Pd(OAc)_2_/Ag_2_CO_3_ (**Entry 2**) and PhI = O/TfOH (**Entry 3**) operate under oxidative conditions and take 12–24 h, delivering lower yields of 69%–77%, and show no recyclability. While Fe_3_O_4_@AMNA-CuBr (**Entry 5**) achieves the same yield and reusability as GO/MNPs-TEA-CuI, it requires 5 h under PEG and oxygen atmosphere, making the process more complex and potentially less green. Thus, in the synthesis of triphenyl oxazole, the GO/MNPs-TEA-CuI catalyst also proves to be the most efficient, eco-friendly, and robust, offering a combination of short reaction time, high yield, and excellent recyclability under simple conditions.

## 4 Conclusion

The GO/MNPs–TEA–CuI nanocatalyst has proven to be a highly efficient, stable, and reusable catalytic system for the synthesis of highly substituted imidazoles and oxazoles. The catalyst design—featuring graphene oxide-supported magnetic nanoparticles functionalized with triethanolamine and copper(I) iodide—offers a unique combination of high surface area, excellent dispersibility in aqueous media, and strong catalytic activity. These characteristics enable smooth multicomponent reactions under mild and environmentally benign conditions. The reactions were carried out in water under reflux, a green and safe solvent choice that supports sustainable chemistry goals. Notably, high product yields (77%–99%) were obtained within relatively short reaction times (15–80 min for imidazoles and 1–8 h for oxazoles), highlighting the catalyst’s remarkable efficiency. The system demonstrated broad substrate compatibility, accommodating a wide range of aryl aldehydes, aryl nitriles, and terminal alkynes—including those bearing electron-donating, electron-withdrawing, and heteroaromatic groups—without compromising yield or selectivity. Furthermore, the high turnover number (TON) and turnover frequency (TOF) values underscore the catalytic proficiency and robustness of the system. The ease of catalyst recovery via magnetic separation and its consistent reusability across multiple cycles add to its practical advantages. Altogether, this protocol offers a green, scalable, and highly versatile approach for the synthesis of valuable heterocycles, reinforcing the central role of GO/MNPs–TEA–CuI as a powerful nanocatalyst in modern synthetic methodology.

### 4.1 Advantages of the method


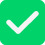
 Green Chemistry Approach: Reactions are performed in water without using toxic organic solvents or additives.


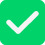
 Broad Substrate Scope: Works well with electron-donating, electron-withdrawing, and heteroaryl substrates.


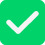
High Efficiency: High yields (77%–99%), low catalyst loading (6 mol%), and fast reaction times.


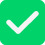
 Mild Reaction Conditions: Requires only reflux in water with no harsh reagents, making it safer and energy-efficient.


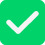
 Recyclable Catalyst: The magnetic GO/MNPs–TEA–CuI catalyst can be easily separated and potentially reused.


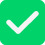
 High TON and TOF: The catalyst exhibits excellent productivity, as demonstrated by high Turnover Number (TON) and Turnover Frequency (TOF).


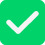
 Versatile Synthetic Utility: Applicable to the synthesis of imidazoles and oxazoles, which are key scaffolds in medicinal and organic chemistry.


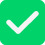
 Environmentally Friendly and Scalable: Simple workup and purification, minimal waste generation, and potential for large-scale applications.

## Data Availability

The original contributions presented in the study are included in the article/Supplementary Material, further inquiries can be directed to the corresponding authors.
