# Supplementary material for: GO/MNPs–TEA–CuI in water: a green and efficient catalytic system for multicomponent preparation of highly substituted imidazoles and oxazoles
Source: Front Chem. 2026 Feb 5;13:1732911. doi: 10.3389/fchem.2025.1732911 (PMC12919339; doi:10.3389/fchem.2025.1732911)

**GO/MNPs–TEA–CuI in Water: A Green and Efficient Catalytic System for Multicomponent Preparation of Highly Substituted Imidazoles and Oxazoles**

**Mohamed Abu Shuheil *^1^**, Ahmed Aldulaimi ^2^, Rekha M M ^3^, Subhashree Ray ^4^, Omayma Salim Waleed ^5^, C P. Surya ^6^, **Renu Sharma ^7^*,** Vatsal Jain ^8^

1

**Mohamed Abu Shuheil (Corresponding Author)**

**Faculty of Allied Medical Sciences, Hourani Center for Applied Scientific Research, Al-Ahliyya Amman University, Amman, Jordan.**

**Mail:** [**m.abushuhel@ammanu.edu.jo**](mailto:m.abushuhel@ammanu.edu.jo) **or mohamedabushuheil@gmail.com**

2

Ahmed Aldulaimi

Faculty of Pharmacy, Al-Zahrawi University, Karbala, Iraq.

**Mail: Ahmedaldulaimi1@gmai.com**

3

Rekha M M

Department of Chemistry and Biochemistry, School of Sciences, JAIN (Deemed to be University), Bangalore, Karnataka, India.

**Mail: mm.rekha@jainuniversity.ac.in**

4

Subhashree Ray

Department of Biochemistry, IMS and SUM Hospital, Siksha 'O' Anusandhan, Bhubaneswar, Odisha-751003, India.

**Mail: subhashreeray@soa.ac.in**

5

Omayma Salim Waleed

Department of Anesthesia Techniques, health and medical techniques college, Alnoor University, Mosul, Iraq.

**Mail: omayma.salim@alnoor.edu.iq**

6

C P. Surya

Department of Chemistry, Sathyabama Institute of Science and Technology, Chennai, Tamil Nadu, India.

**Mail: surya.cp.chemistry@sathyabama.ac.in**

7

**Renu Sharma (Co-corresponding Author)**

**Department of Chemistry, University Institute of Sciences, Chandigarh University, Mohali, Punjab, India.**

**Mail: drrenusharma01@outlook.com**

8

Vatsal Jain

Centre for Research Impact & Outcome, Chitkara University Institute of Engineering and Technology, Chitkara University, Rajpura, 140401, Punjab, India.

**Mail: VATSALJAIN0202@outlook.com**

**NMR Data for imidazole Products**

**2-(4-chlorophenyl)-4,5-diphenyl-1H-imidazole**

**(M.P: 259-261 ^o^C)**

^1^H NMR (400 MHz, DMSO) δ 13.82 (s, 1H), 8.43 (d, *J* = 8.0 Hz, 2H), 7.57-7.48 (m, 8H), 7.32-7.15 (m, 4H); ^13^C NMR (100 MHz, DMSO) δ 177.1, 138.1, 134.4, 131.8, 128.0, 127.6, 126.2 ppm.

**2,4,5-triphenyl-1*H*-imidazole**

**(M.P: 269-271 ^o^C)**

^1^H NMR (400 MHz, DMSO) δ 13.81 (s, 1H), 8.49 (d, *J* = 7.7 Hz, 2H), 7.62-7.51 (m, 9H), 7.49-7.40 (m, 4H); ^13^C NMR (100 MHz, DMSO) δ 177.4, 138.1, 134.5, 131.0, 128.7, 128.1, 127.6 ppm.

**4,5-diphenyl-2-(p-tolyl)-1*H*-imidazole**

**(M.P: 231-233 ^o^C)**

^1^H NMR (400 MHz, DMSO) δ 13.82 (s, 1H), 8.48 (d, *J* = 7.4 Hz, 2H), 7.58-7.40 (m, 8H), 7.39-7.30 (m, 4H), 2.43 (s, 3H); ^13^C NMR (100 MHz, DMSO) δ 177.0, 138.1, 130.6, 129.5, 128.3, 128.1, 127.4, 21.8 ppm.

**4-(4,5-diphenyl-1H-imidazol-2-yl)benzonitrile**

**(M.P: 230-232 ^o^C)**

^1^H NMR (400 MHz, DMSO) δ 13.83 (s, 1H), 8.01 (d, *J* = 7.6 Hz, 2H), 7.77 (d, *J* = 8.0 Hz, 2H), 7.54-7.38 (m, 6H), 7.16-7.04 (m, 4H); ^13^C NMR (100 MHz, DMSO) δ 177.6, 140.3, 138.1, 130.8, 129.6, 129.1, 128.3, 128.0, 127.1, 119.8, 116.9 ppm.

**2-(3-nitrophenyl)-4,5-diphenyl-1H-imidazole**

**(M.P: 313-315 ^o^C)**

^1^H NMR (400 MHz, DMSO) δ 13.84 (s, 1H), 7.97 (d, *J* = 8.6 Hz, 2H), 7.72 (d, *J* = 7.4 Hz, 1H), 7.53 (t, *J* = 8.0 Hz, 1H), 7.47-7.36 (m, 6H), 7.23-7.11 (m, 4H); ^13^C NMR (100 MHz, DMSO) δ 177.4, 148.7, 138.1, 132.0, 131.9, 130.5, 129.4, 128.5, 128.2, 127.3, 124.7, 123.6 ppm.

**2-(2-chlorophenyl)-4,5-diphenyl-1H-imidazole**

**(M.P: 187-189 ^o^C)**

^1^H NMR (400 MHz, DMSO) δ 13.79 (s, 1H), 8.04 (d, *J* = 7.4 Hz, 1H), 7.96 (d, *J* = 7.6 Hz, 1H), 7.63-7.56 (m, 4H), 7.49-7.34(m, 4H), 7.22-7.14 (m, 4H); ^13^C NMR (100 MHz, DMSO) δ 158.6, 139.0, 138.7, 132.6, 130.8, 127.5, 126.0, 125.6, 124.1 ppm.

**2-(4,5-diphenyl-1H-imidazol-2-yl)phenol**

**(M.P: 203-205 ^o^C)**

^1^H NMR (400 MHz, DMSO) δ 13.55 (s, 1H), 9.62 (s, 1H), 7.68 (d, *J* = 7.4 Hz, 2H), 7.55-7.42 (m, 5H), 7.38-7.27 (m, 3H), 7.11-7.02 (m, 4H); ^13^C NMR (100 MHz, DMSO) δ 155.8, 147.1, 138.5, 132.4, 129.7, 129.2, 128.4, 128.1, 127.0, 121.3, 118.1, 117.9 ppm.

**2-(2,4-dichlorophenyl)-4,5-diphenyl-1H-imidazole**

**(M.P: 170-172 ^o^C)**

^1^H NMR (400 MHz, DMSO) δ 13.54 (s, 1H), 7.91-7.80 (m, 2H), 7.62-7.50 (m, 6H), 7.10-7.02 (m, 5H); ^13^C NMR (100 MHz, DMSO) δ 148.2, 138.6, 137.5, 134.7, 131.9, 130.3, 129.4, 128.2, 126.1, 126.0, 125.4 ppm.

**4-(4,5-diphenyl-1H-imidazol-2-yl)pyridine**

**(M.P: 231-233 ^o^C)**

^1^H NMR (400 MHz, DMSO) δ 13.58 (s, 1H), 8.81 (d, *J* = 7.7 Hz, 2H), 8.16 (d, *J* = 7.6 Hz, 2H), 7.66-7.52 (m, 6H), 7.19-7.08 (m, 4H); ^13^C NMR (100 MHz, DMSO) δ 150.8, 146.1, 138.7, 130.6, 130.1, 129.5, 129.2, 128.9, 120.6 ppm.

**2-(benzo[d][1,3]dioxol-5-yl)-4,5-diphenyl-1H-imidazole**

**(M.P: 255-257 ^o^C)**

^1^H NMR (400 MHz, DMSO) δ 13.67 (s, 1H), 7.74 (d, *J* = 8.2 Hz, 1H), 7.65-7.40 (m, 6H), 7.39-7.31 (m, 5H), 7.03(d, *J* = 8.0 Hz, 1H), 6.04 (s, 2H); ^13^C NMR (100 MHz, DMSO) δ 150.1, 138.8, 129.7, 129.1, 128.6, 128.4, 125.7, 123.2, 113.9, 111.4, 102.3 ppm.

**4-(4,5-diphenyl-1H-imidazol-2-yl)-2,6-dimethoxyphenol**

**(M.P: 281-283 ^o^C)**

^1^H NMR (400 MHz, DMSO) δ 13.71 (s, 1H), 8.64 (s, 1H), 7.58-7.45 (m, 6H), 7.42-7.33 (m, 4H), 6.68 (s, 2H), 3.81 (s, 6H); ^13^C NMR (100 MHz, DMSO) δ 178.1, 151.0, 139.6, 138.2, 130.3, 129.5, 128.1, 124.8, 104.3, 57.2 ppm.

2-(naphthalen-2-yl)-4,5-diphenyl-1H-imidazole

**(M.P: 278-280 ^o^C)**

^1^H NMR (400 MHz, DMSO) δ 13.62 (s, 1H), 9.06 (s, 1H), 8.49 (d, *J* = 8.0 Hz, 1H), 8.43 (d, *J* = 7.7 Hz, 1H), 8.09 (d, *J* = 7.6 Hz, 1H), 7.98 (d, *J* = 8.0 Hz, 1H), 7.77-7.67 (m, 6H), 7.53-7.40 (m, 4H), 7.35 (t, *J* = 7.4 Hz, 2H); ^13^C NMR (100 MHz, DMSO) δ 138.7, 135.6, 134.0, 133.2, 132.5, 131.7, 130.1, 129.4, 129.2, 128.9, 127.7, 127.0, 126.5, 126.1 ppm.

2-(2,6-dichlorophenyl)-4,5-diphenyl-1H-imidazole

**(M.P: 234-236 ^o^C)**

^1^H NMR (400 MHz, DMSO) δ 13.63 (s, 1H), 7.86 (t, *J* = 7.7 Hz, 3H), 7.73-7.61 (m, 6H), 7.18-7.04 (m, 4H); ^13^C NMR (100 MHz, DMSO) δ 148.0, 138.7, 137.1, 134.5, 130.8, 129.5, 128.4, 128.2, 127.3, 126.8, 126.1 ppm.

2-(furan-2-yl)-4,5-diphenyl-1H-imidazole

**(M.P: 232-235 ^o^C)**

^1^H NMR (400 MHz, DMSO) δ 13.62 (s, 1H), 8.23 (d, *J* = 8.0 Hz, 2H), 7.89-7.81 (m, 3H), 7.53-7.39 (m, 5H), 6.86-6.79 (m, 3H); ^13^C NMR (100 MHz, DMSO) δ 155.6, 143.2, 141.9, 138.0, 130.7, 129.4, 128.1, 113.5, 107.6 ppm.

**4,5-diphenyl-2-(thiophen-2-yl)-1H-imidazole**

**(M.P: 256-258 ^o^C)**

^1^H NMR (400 MHz, DMSO) δ 13.62 (s, 1H), 7.92 (d, *J* = 7.7 Hz, 1H), 7.85 (d, *J* = 8.0 Hz, 1H), 7.55-7.42 (m, 6H), 7.32-7.09 (m, 5H); ^13^C NMR (100 MHz, DMSO) δ 145.6, 138.7, 132.1, 130.7, 129.8, 129.1, 128.3, 127.4, 127.2, 126.0 ppm.

**3-(4,5-diphenyl-1H-imidazol-2-yl)-1H-indole**

**(M.P: 306-308 ^o^C)**

^1^H NMR (400 MHz, DMSO) δ 13.60 (s, 1H), 12.82 (s, 1H), 8.63 (s, 1H), 8.10 (d, *J* = 7.4 Hz, 2H), 7.99-7.89 (m, 3H), 7.76-7.70 (m, 6H), 7.57-7.40 (m, 3H); ^13^C NMR (100 MHz, DMSO) δ 147.0, 138.1, 135.8, 130.1, 129.7, 129.2, 128.6, 128.3, 127.5, 125.4, 123.1, 122.7, 119.6, 111.7, 105.4 ppm.

**2-(2-nitrophenyl)-4,5-diphenyl-1H-imidazole**

**(M.P: 235-237 ^o^C)**

^1^H NMR (400 MHz, DMSO) δ 13.55 (s, 1H), 8.01 (d, *J* = 7.6 Hz, 2H), 7.79 (t, *J* = 8.3 Hz, 1H), 7.73 (t, *J* = 7.8 Hz, 1H), 7.46-7.41 (m, 4H), 7.29-7.24 (m, 4H); ^13^C NMR (100 MHz, DMSO) δ 150.8, 147.3, 138.1, 135.0, 133.4, 130.7, 129.2, 128.5, 127.6, 126.0, 124.9 ppm.

**2-methyl-4,5-diphenyl-1H-imidazole**

**(M.P: 240-242 ^o^C)**

^1^H NMR (400 MHz, DMSO) δ 12.91 (s, 1H), 77.49-7.38 (m, 6H), 7.29 (t, *J* = 8.4 Hz, 4H), 2.72 (s, 3H); ^13^C NMR (100 MHz, DMSO) δ 193.2, 138.0, 129.7, 128.5, 127.1, 14.3 ppm.

**2-cyclohexyl-4,5-diphenyl-1H-imidazole**

**(M.P: 241-243 ^o^C)**

^1^H NMR (400 MHz, DMSO) δ 12.90 (s, 1H), 7.48-7.42 (m, 6H), 7.26 (t, *J* = 8.0 Hz, 4H), 2.73-2.69 (m, 1H), 1.63 (t, *J* = 7.6 Hz, 4H), 1.53-1.45 (m, 6H); ^13^C NMR (100 MHz, DMSO) δ 149.3, 138.2, 130.8, 129.1, 128.4, 127.0, 40.7, 33.4, 26.3, 25.1 ppm.

**2-(4-nitrophenyl)-4,5-diphenyloxazole**

**(M.P: 143-145 ^o^C)**

^1^H NMR (400 MHz, DMSO) δ 8.29 (d, *J* = 7.7 Hz, 2H), 8.02 (d, *J* = 8.0 Hz, 2H), 7.46 (d, *J* = 7.4 Hz, 4H), 7.27-7.12 (m, 6H); ^13^C NMR (100 MHz, DMSO) δ 159.6, 147.2, 138.0, 135.4, 130.3, 129.8, 128.1, 127.5, 124.0 ppm.


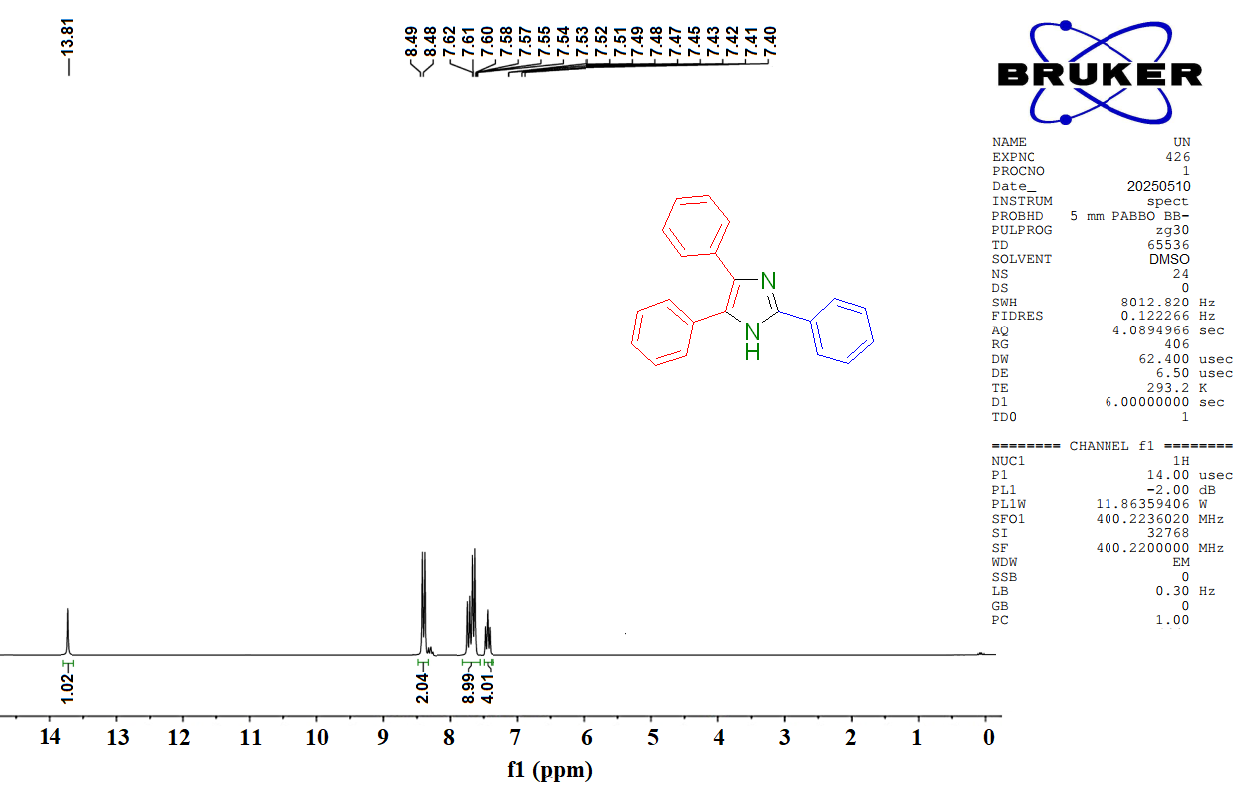


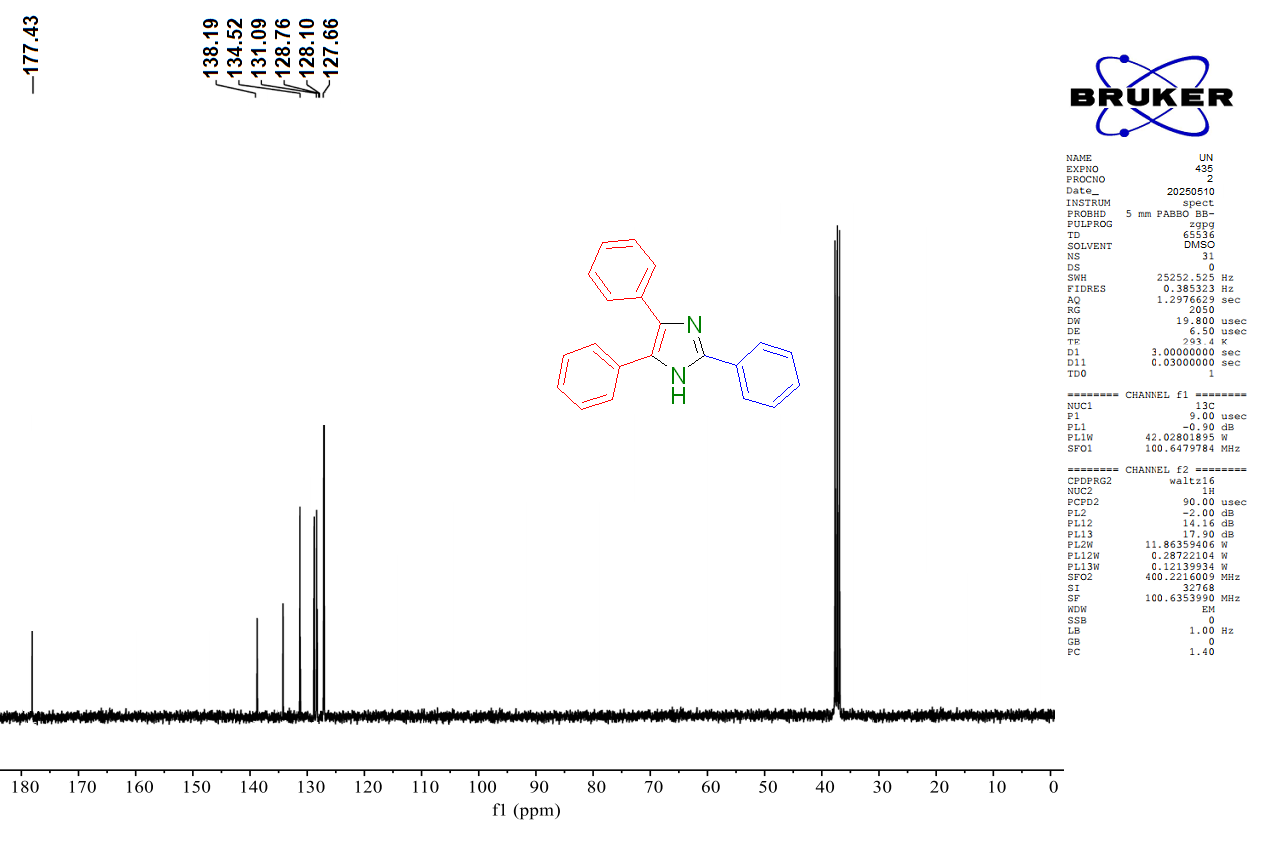


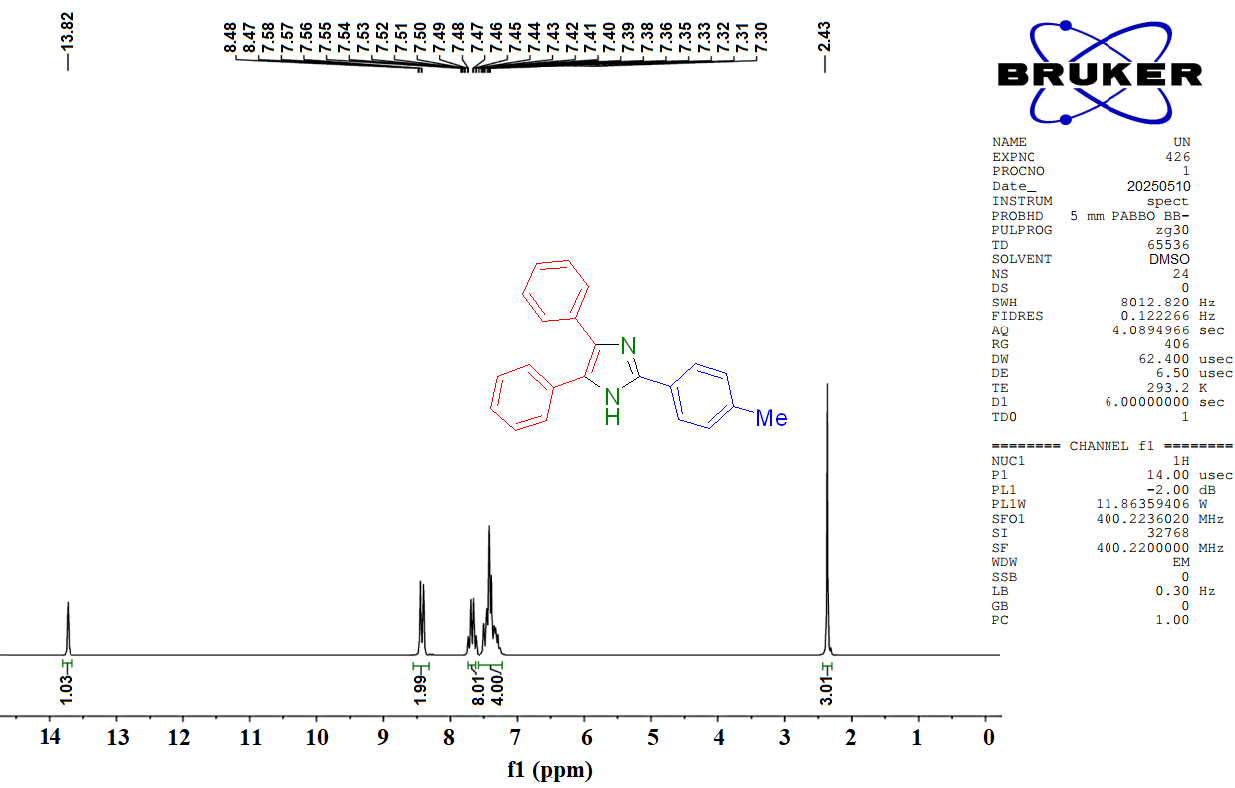


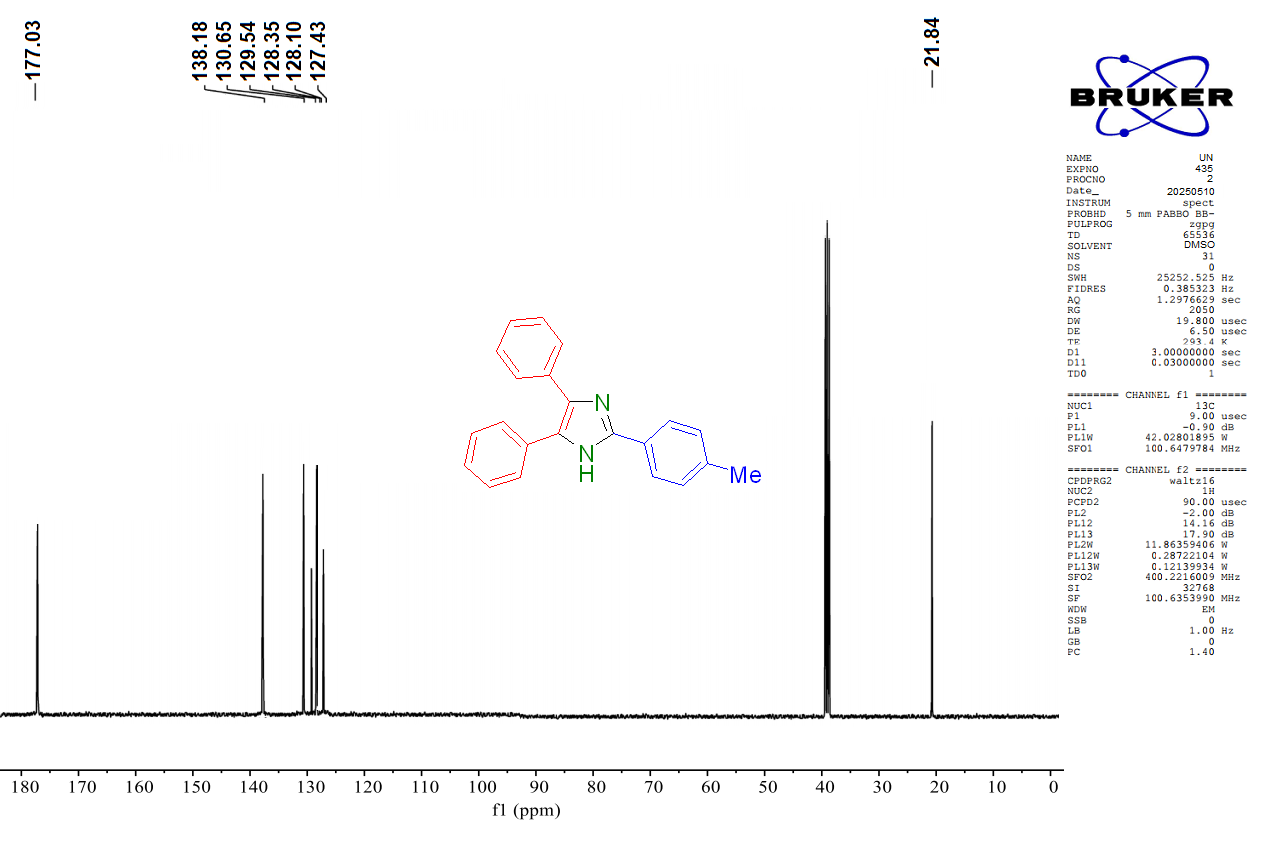


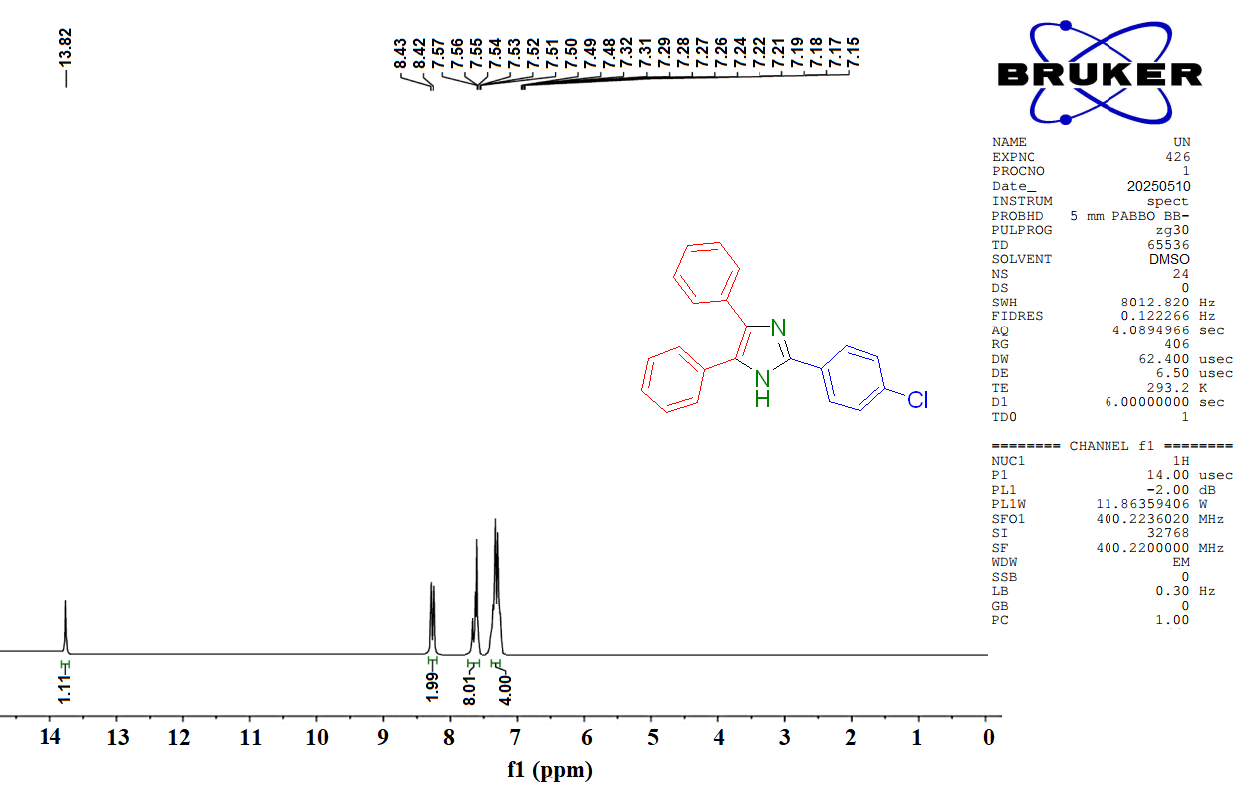


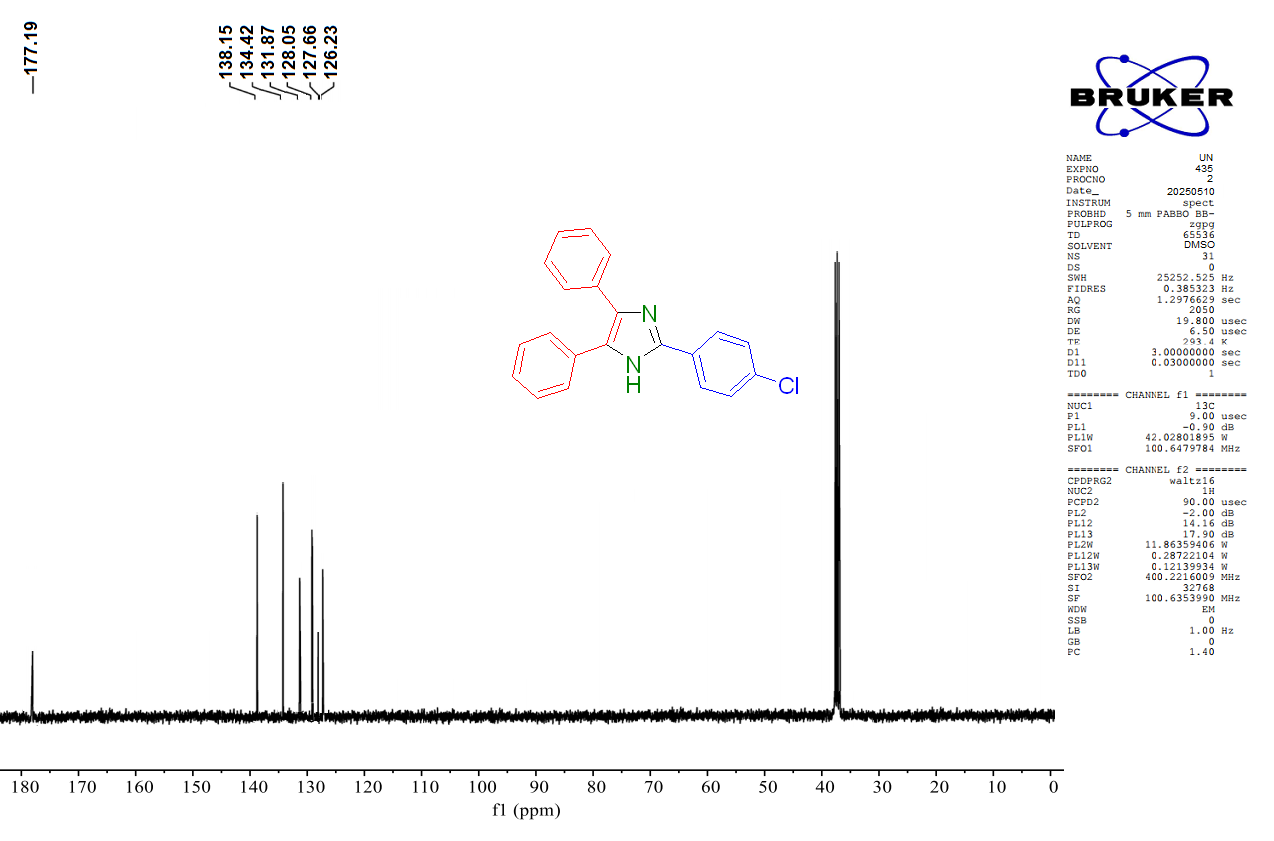


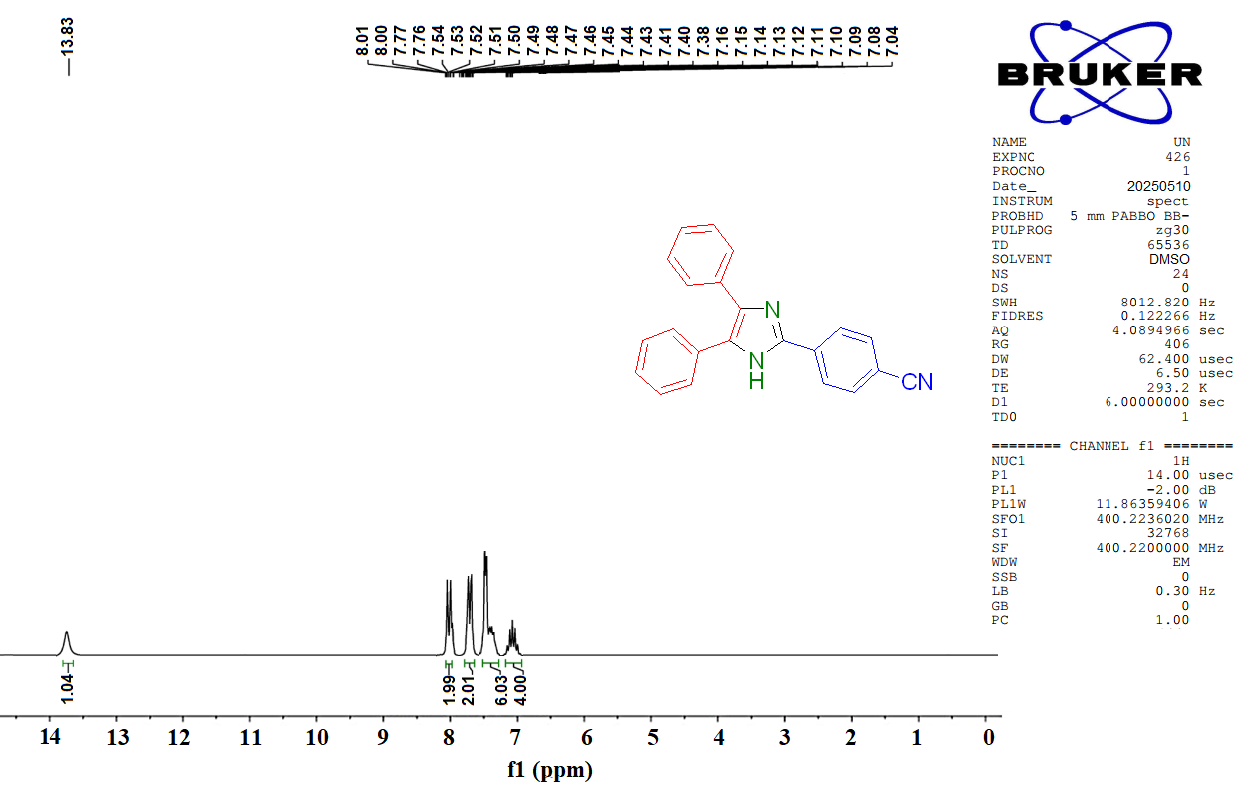


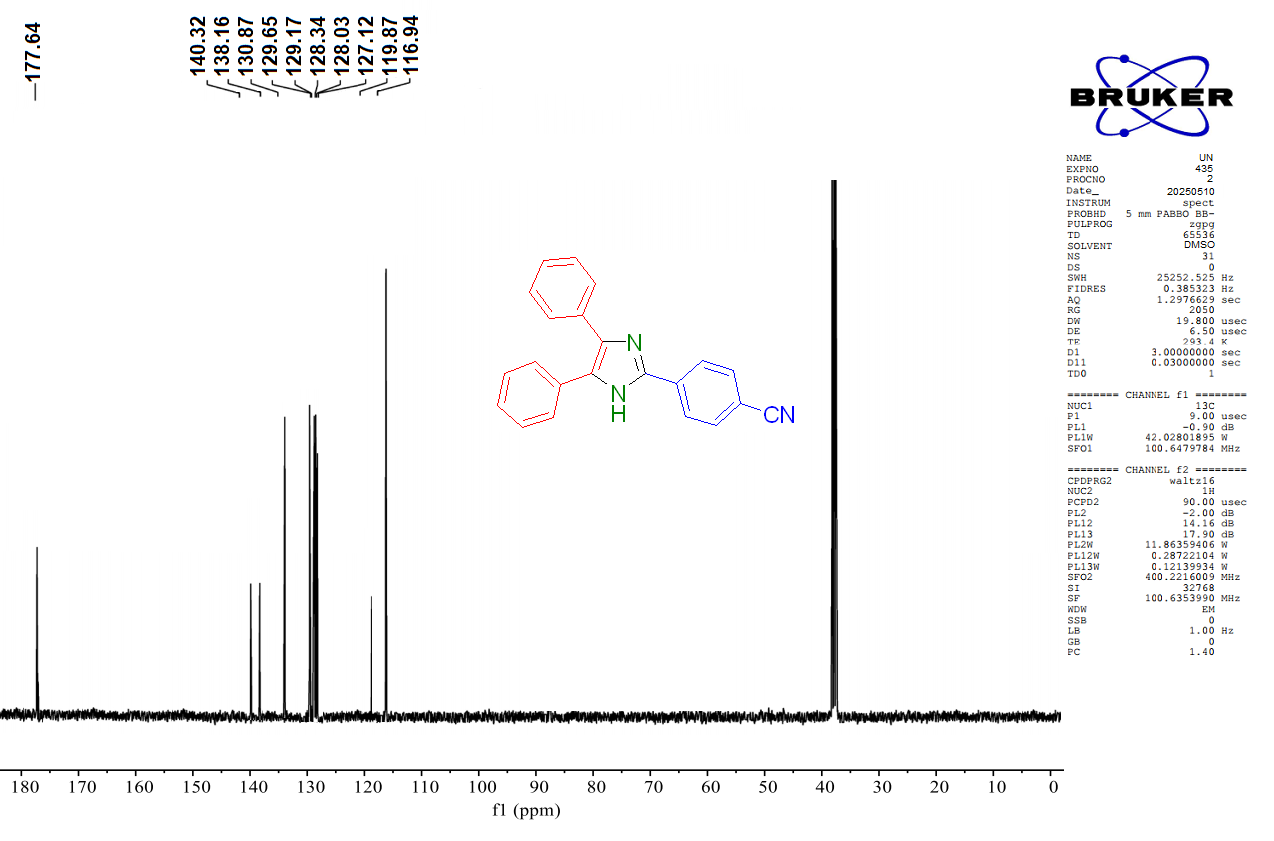


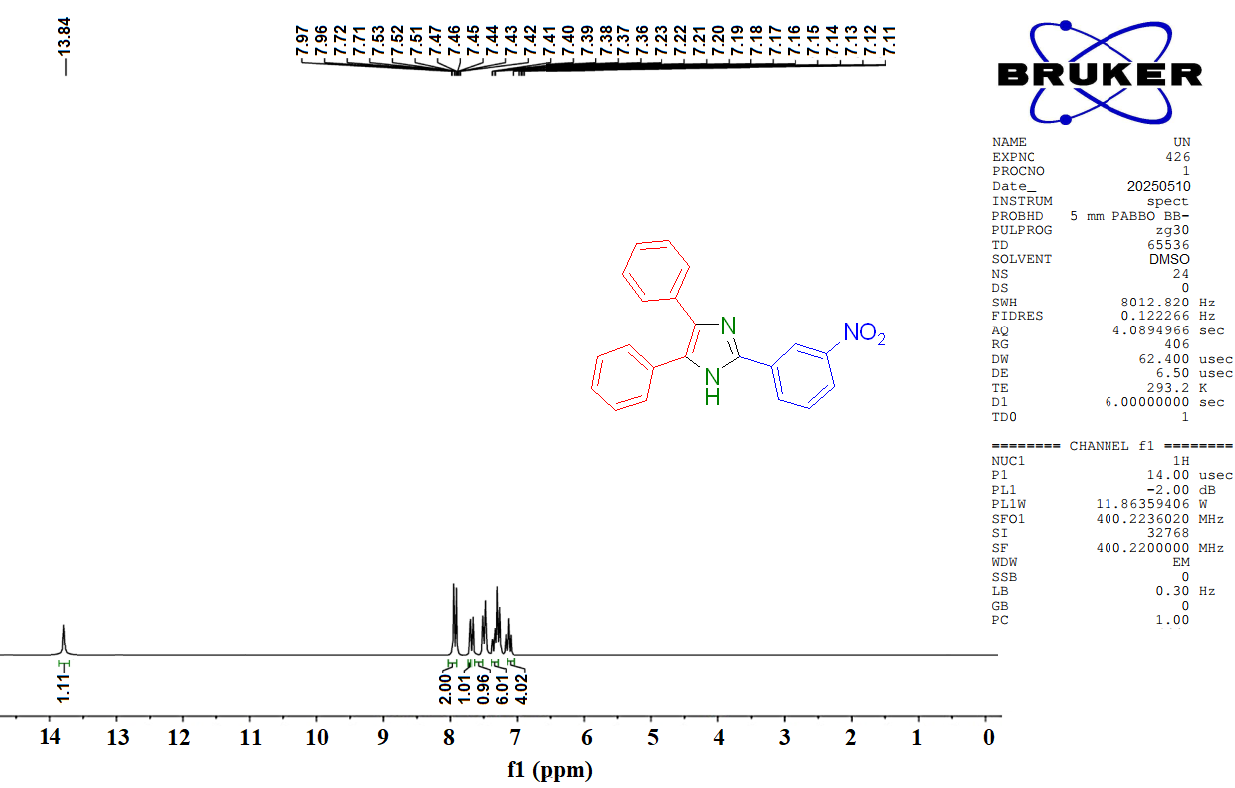


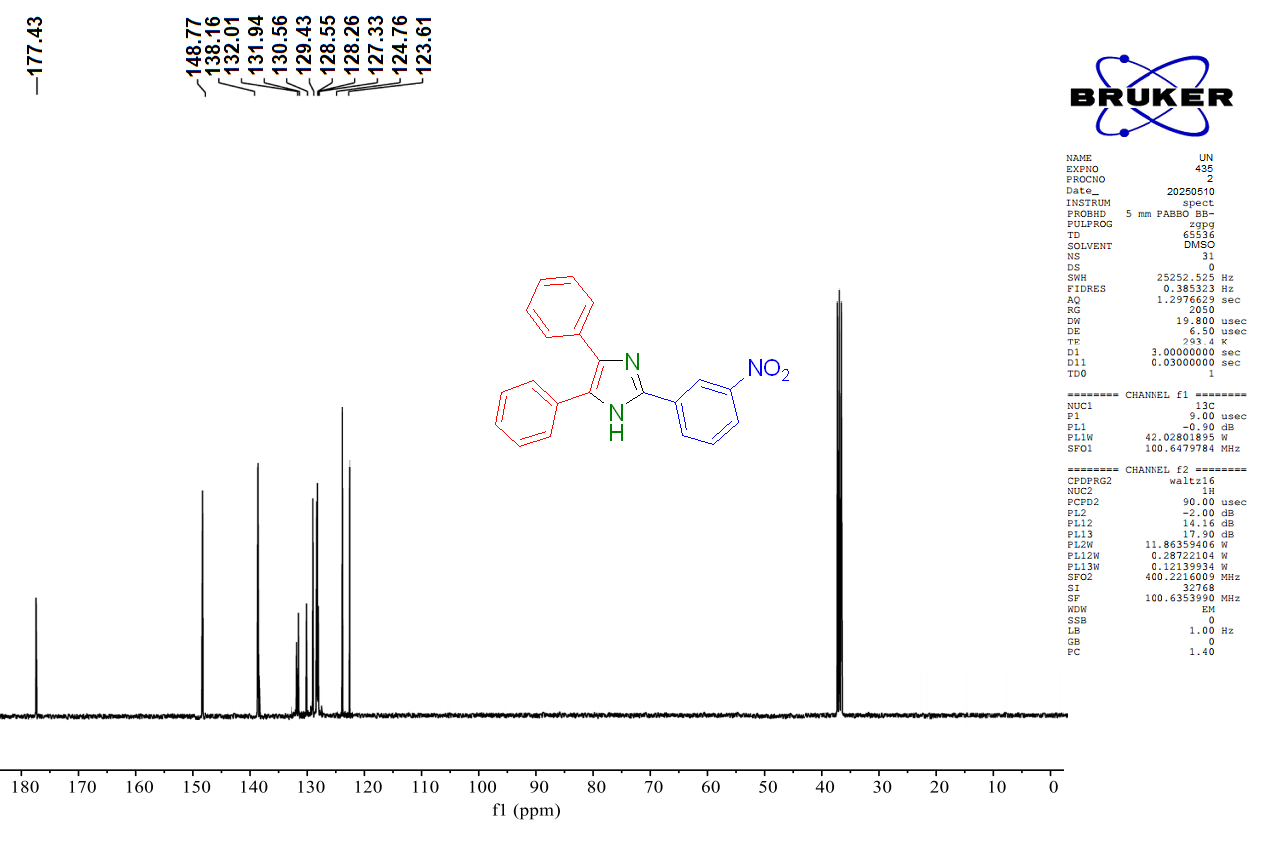


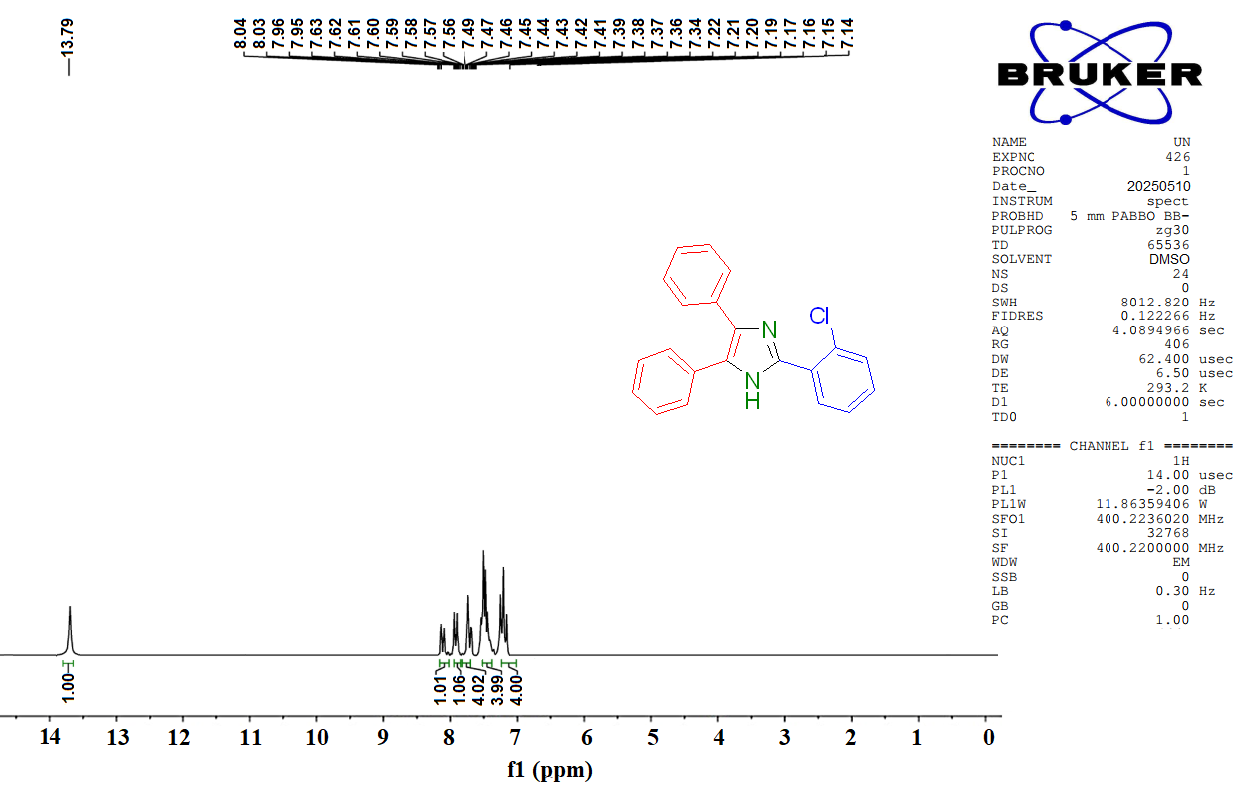


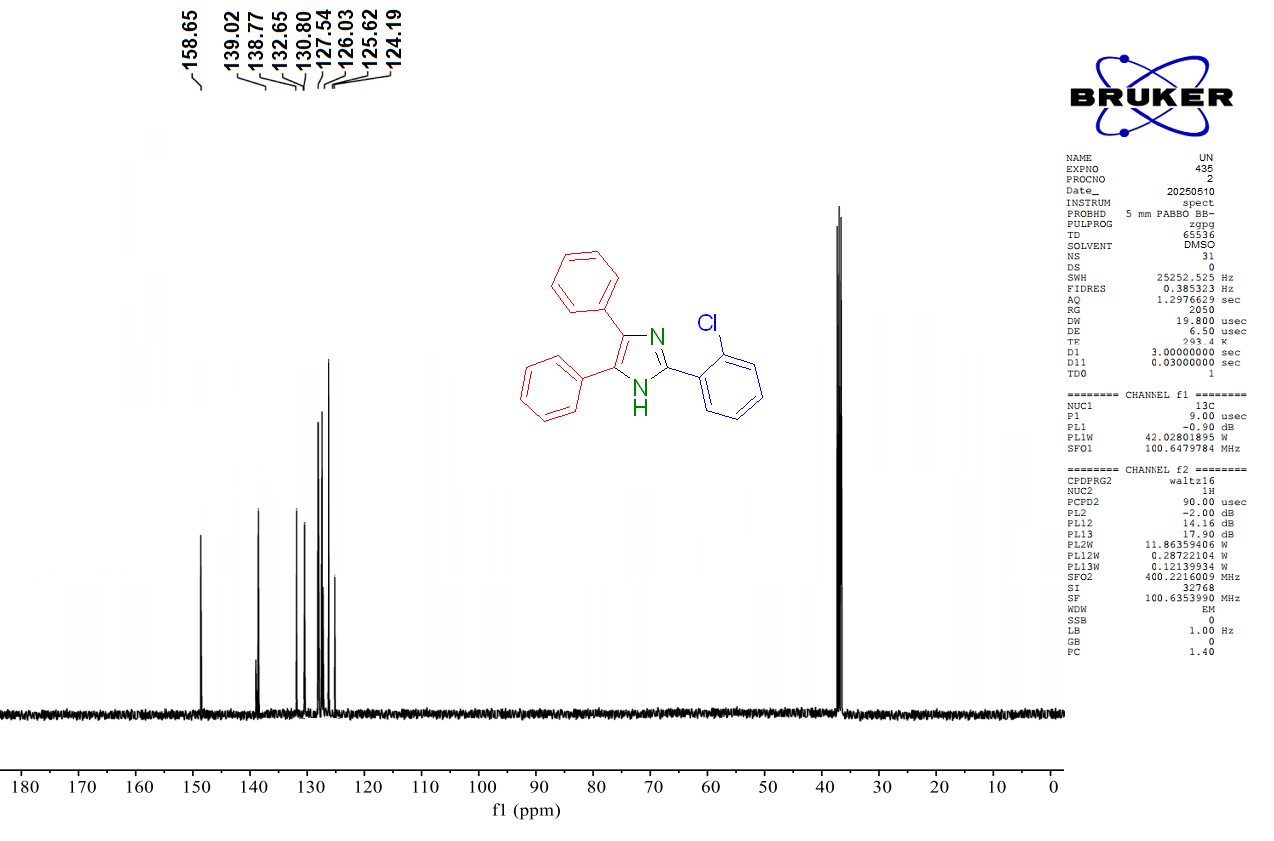


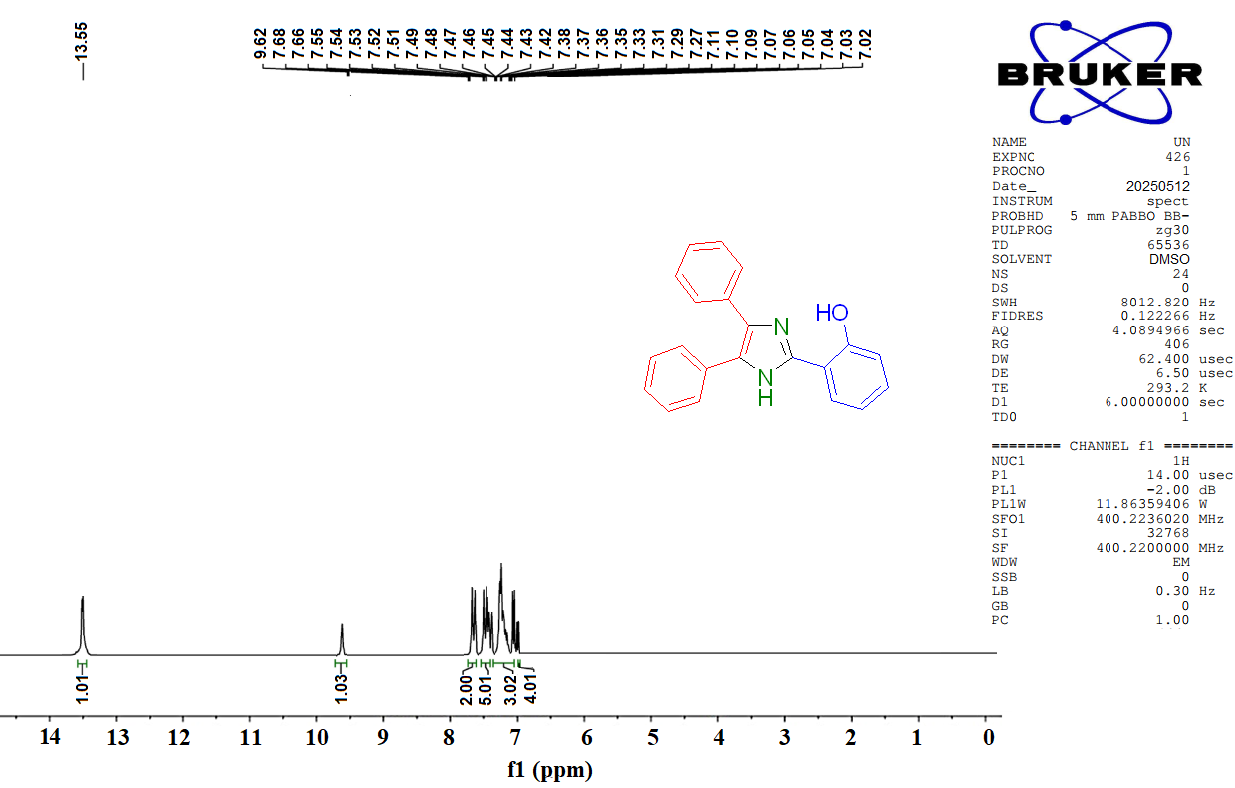


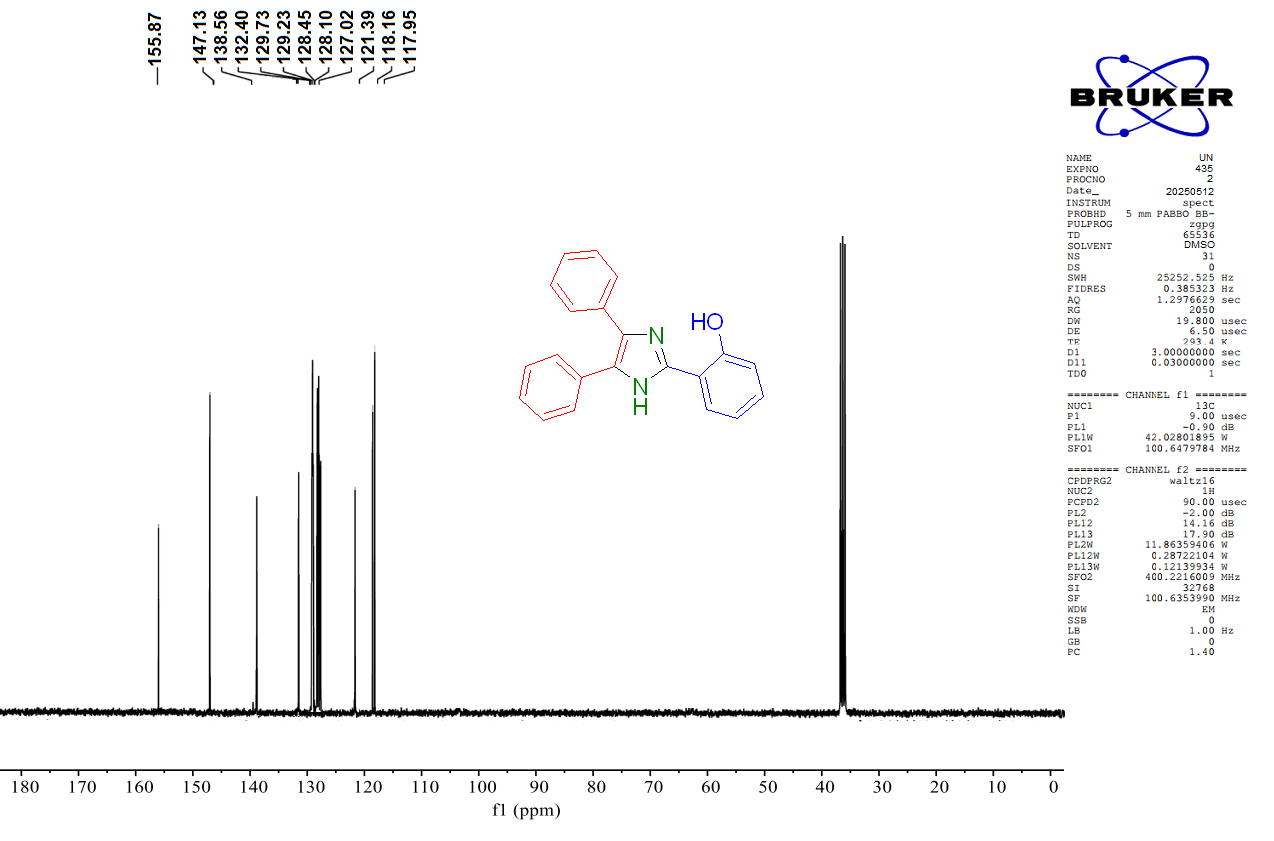


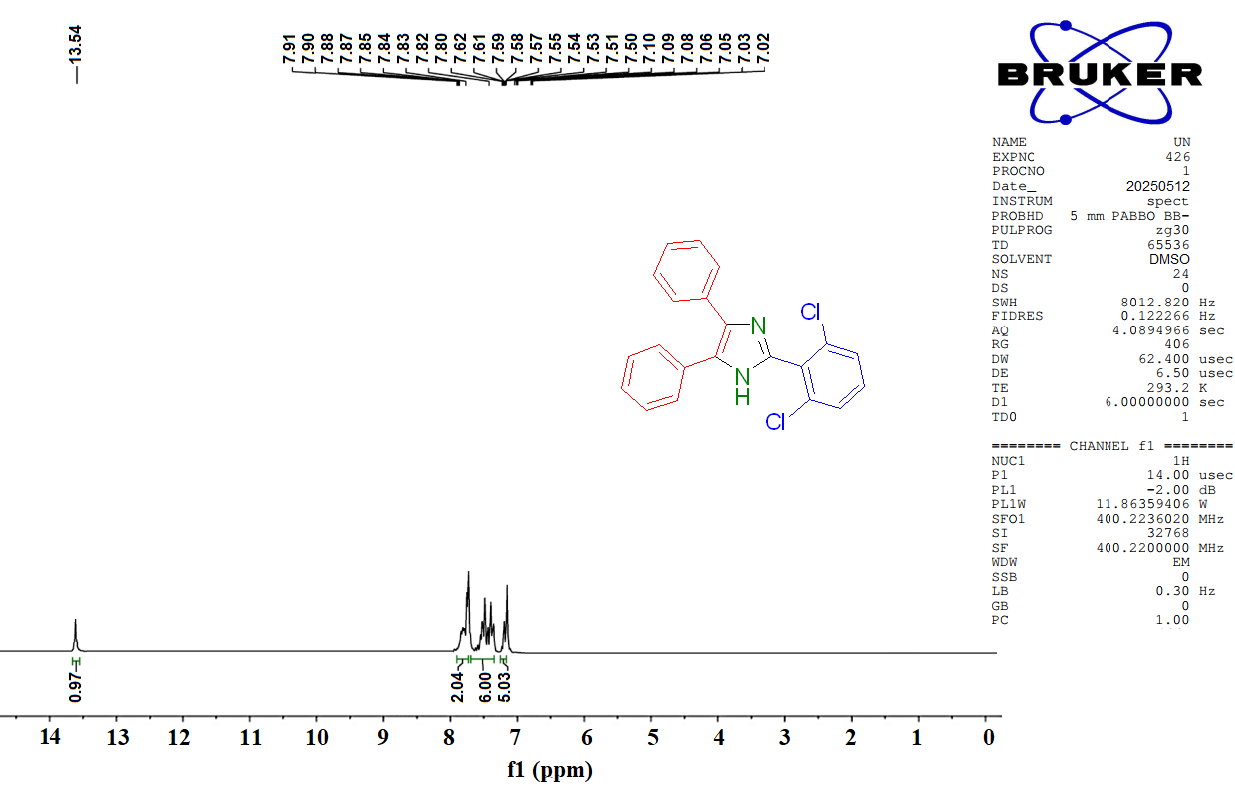


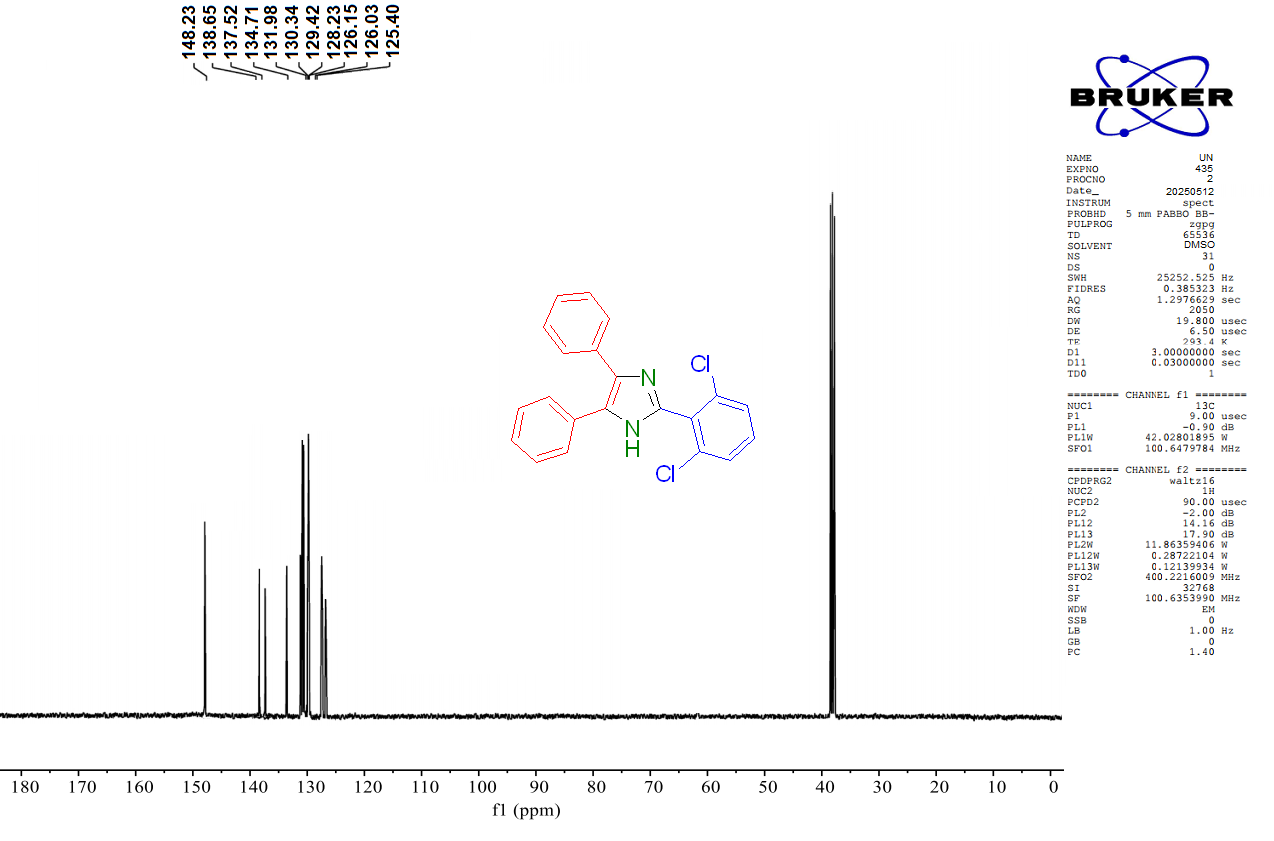


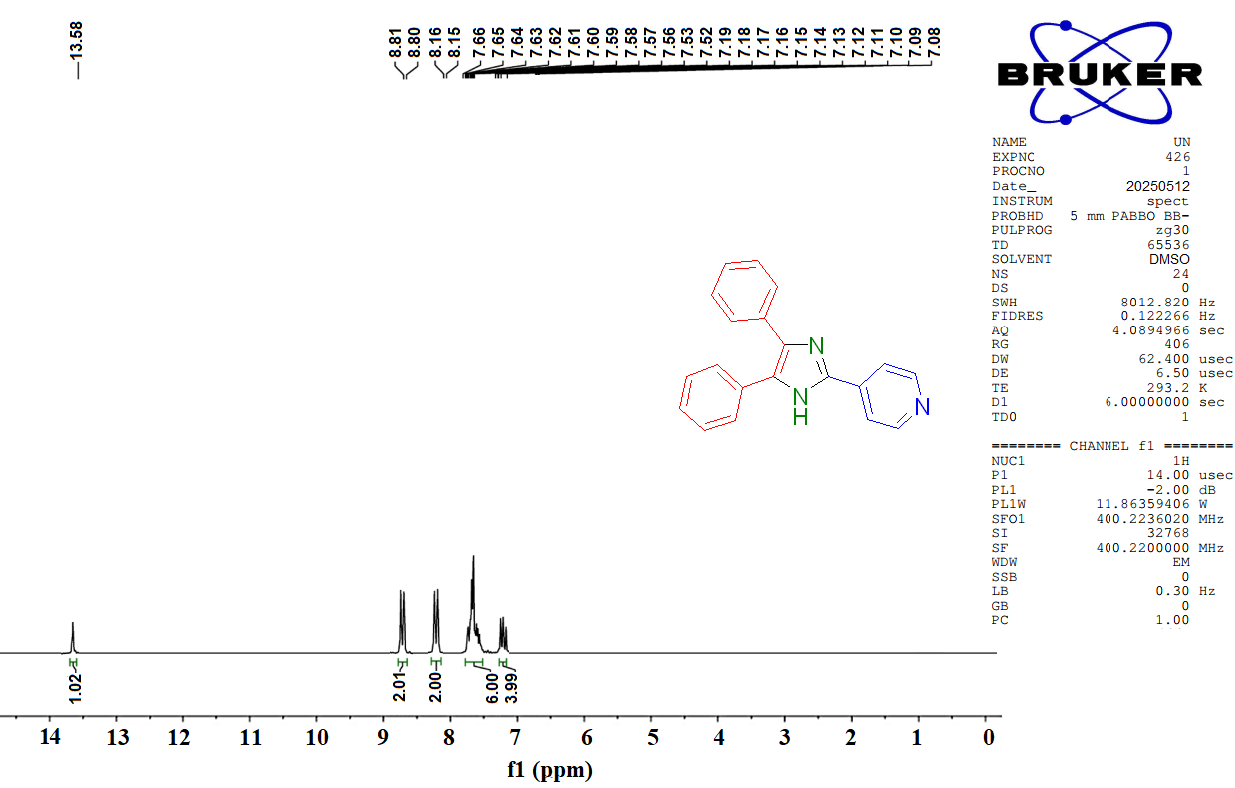


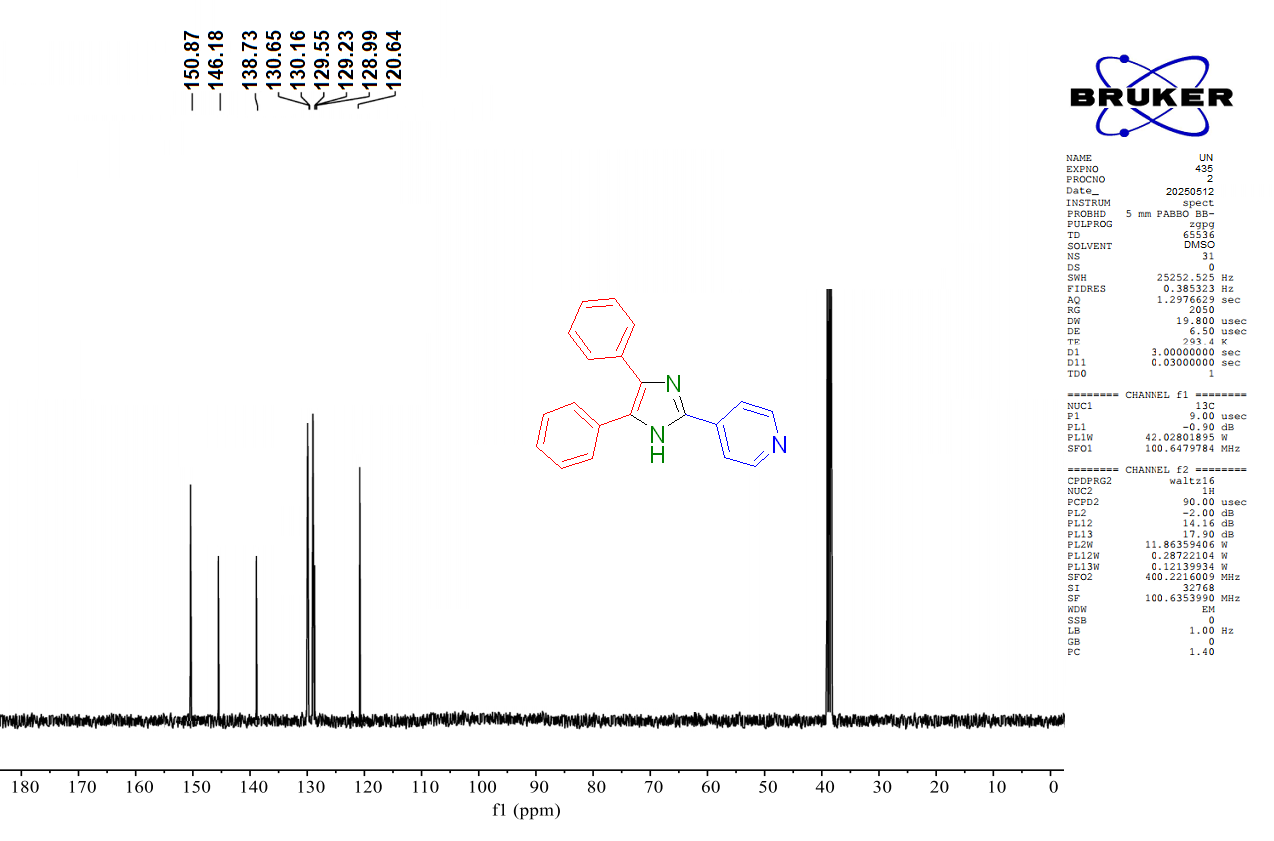


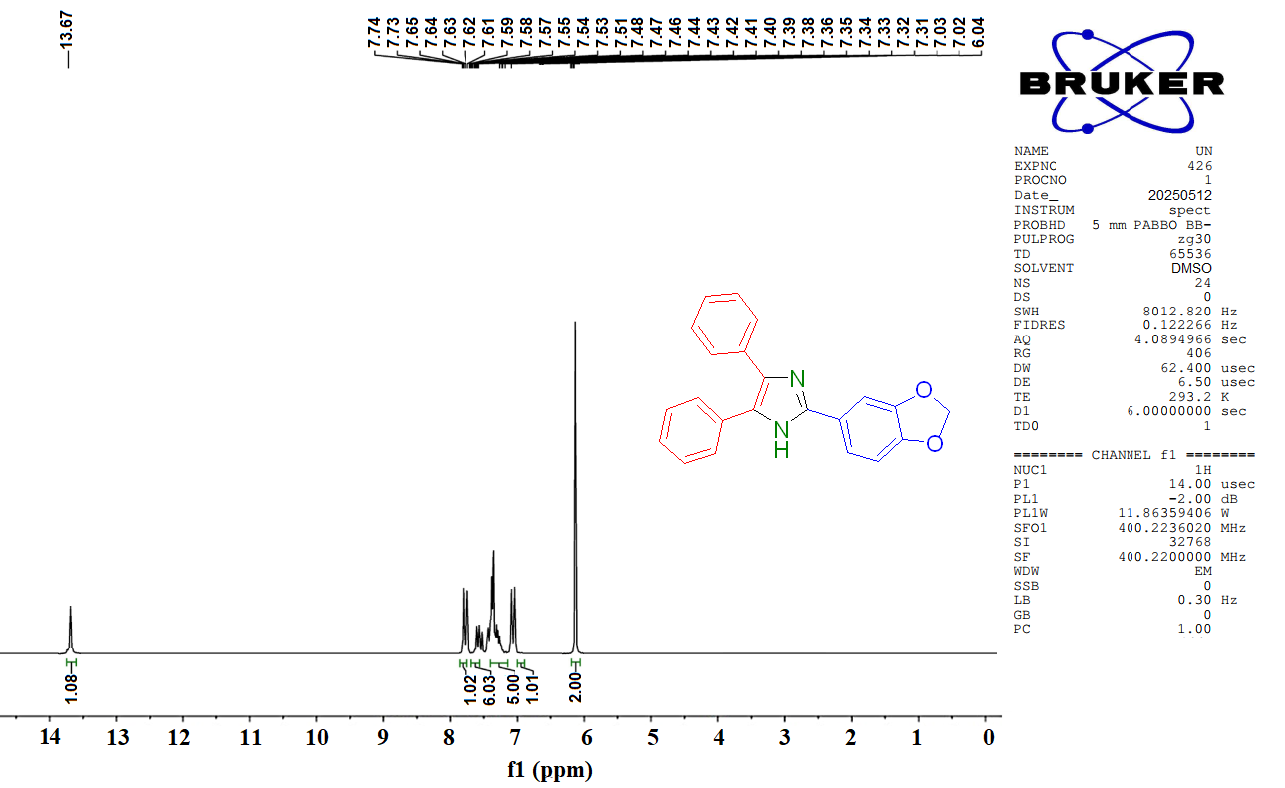


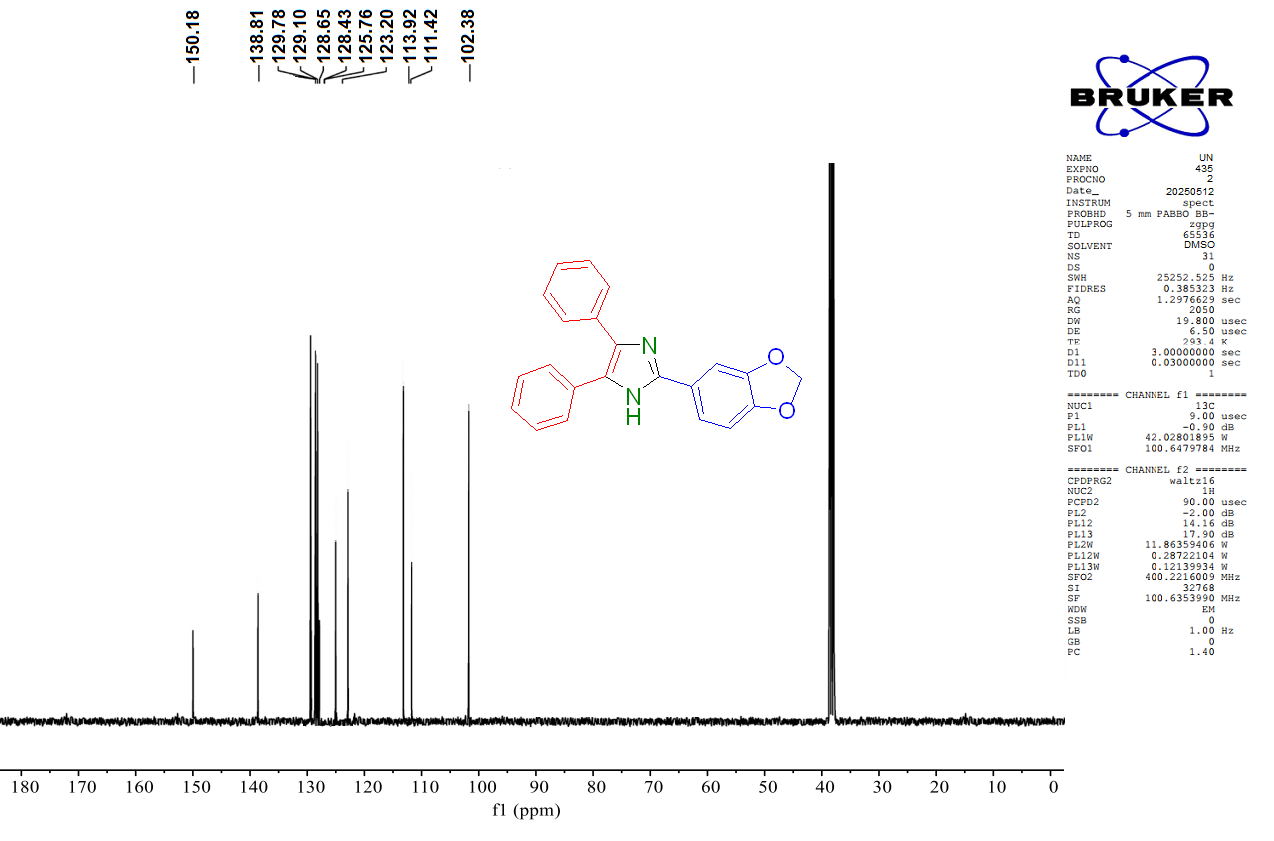


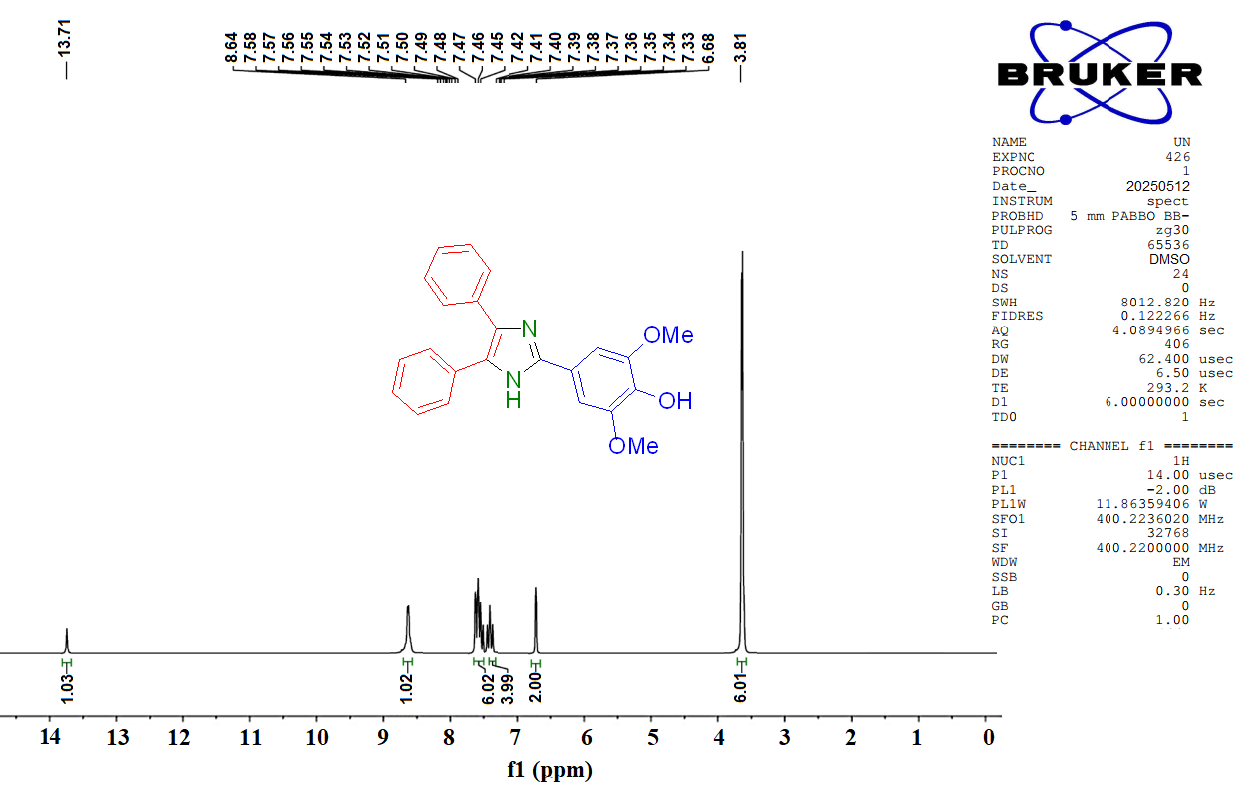


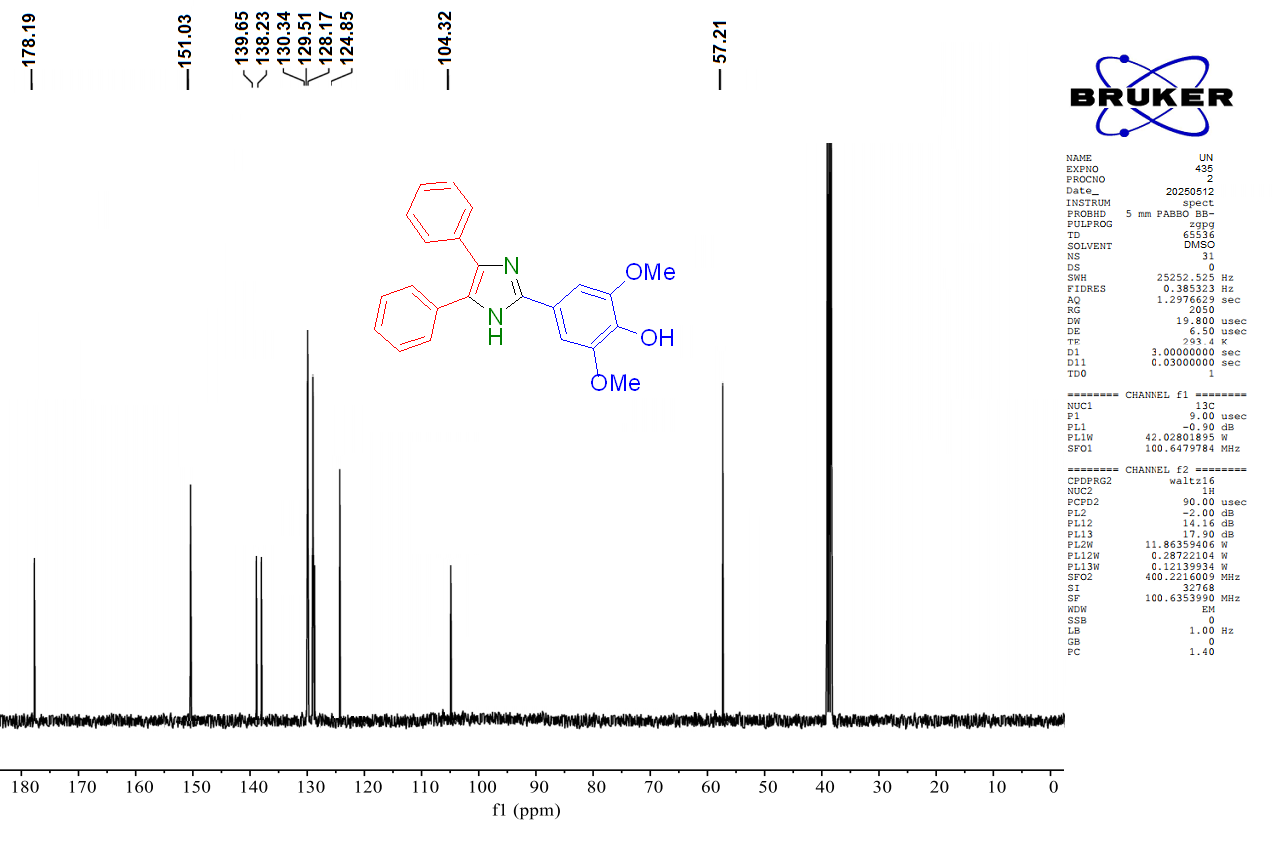


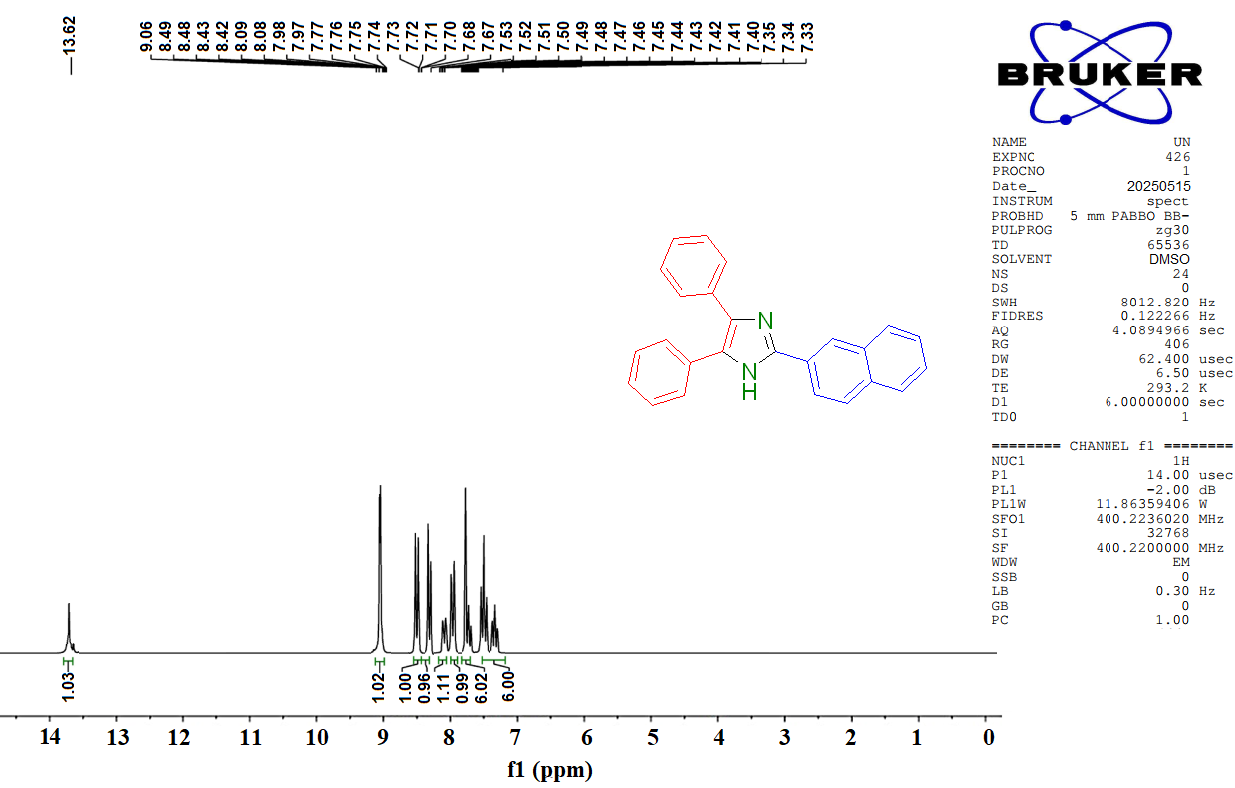


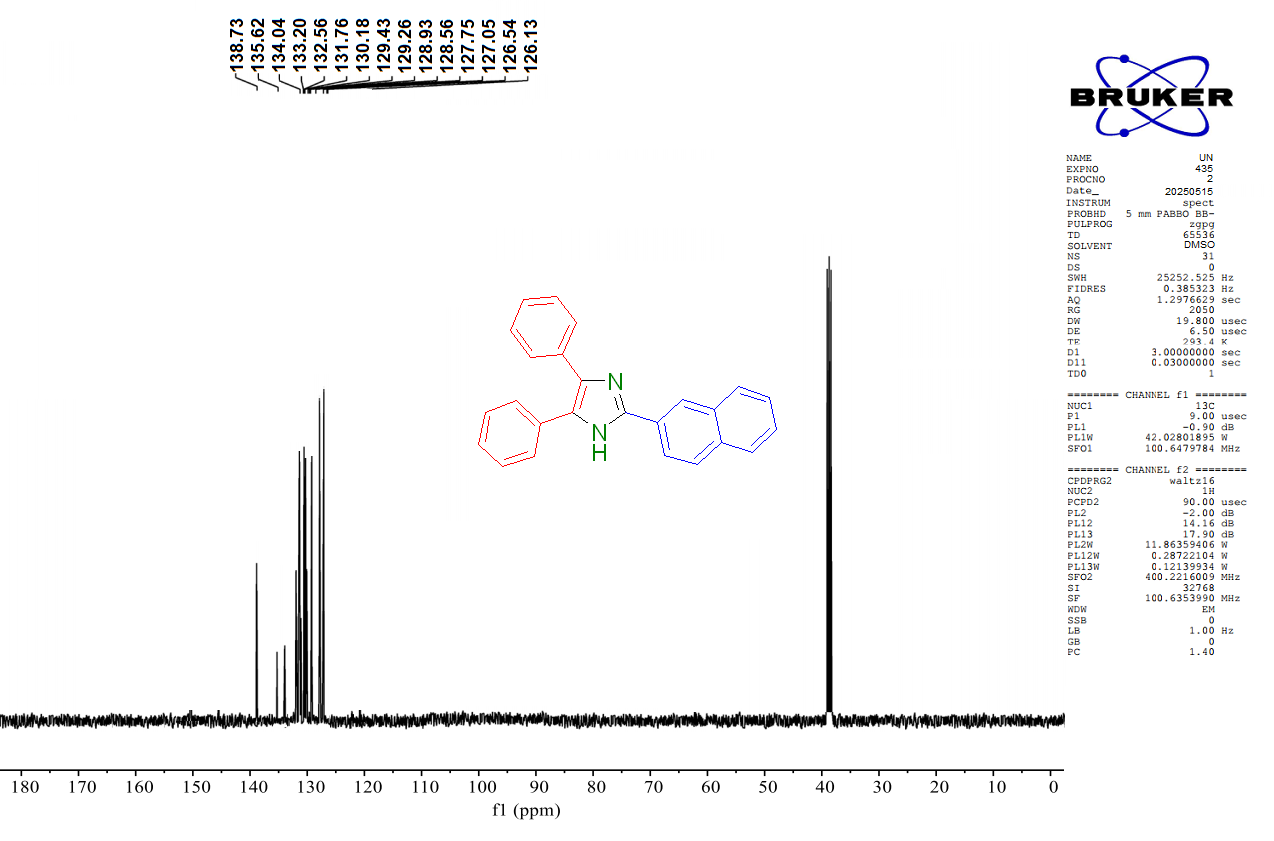


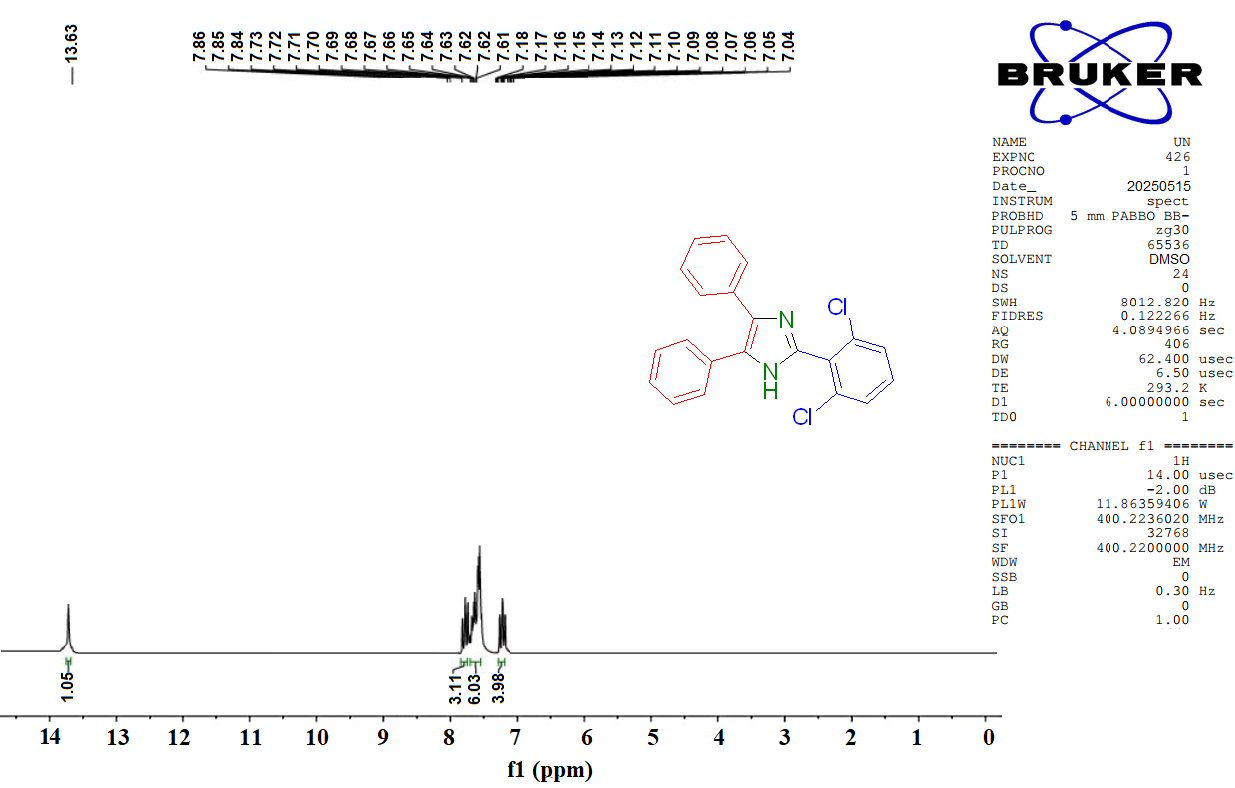


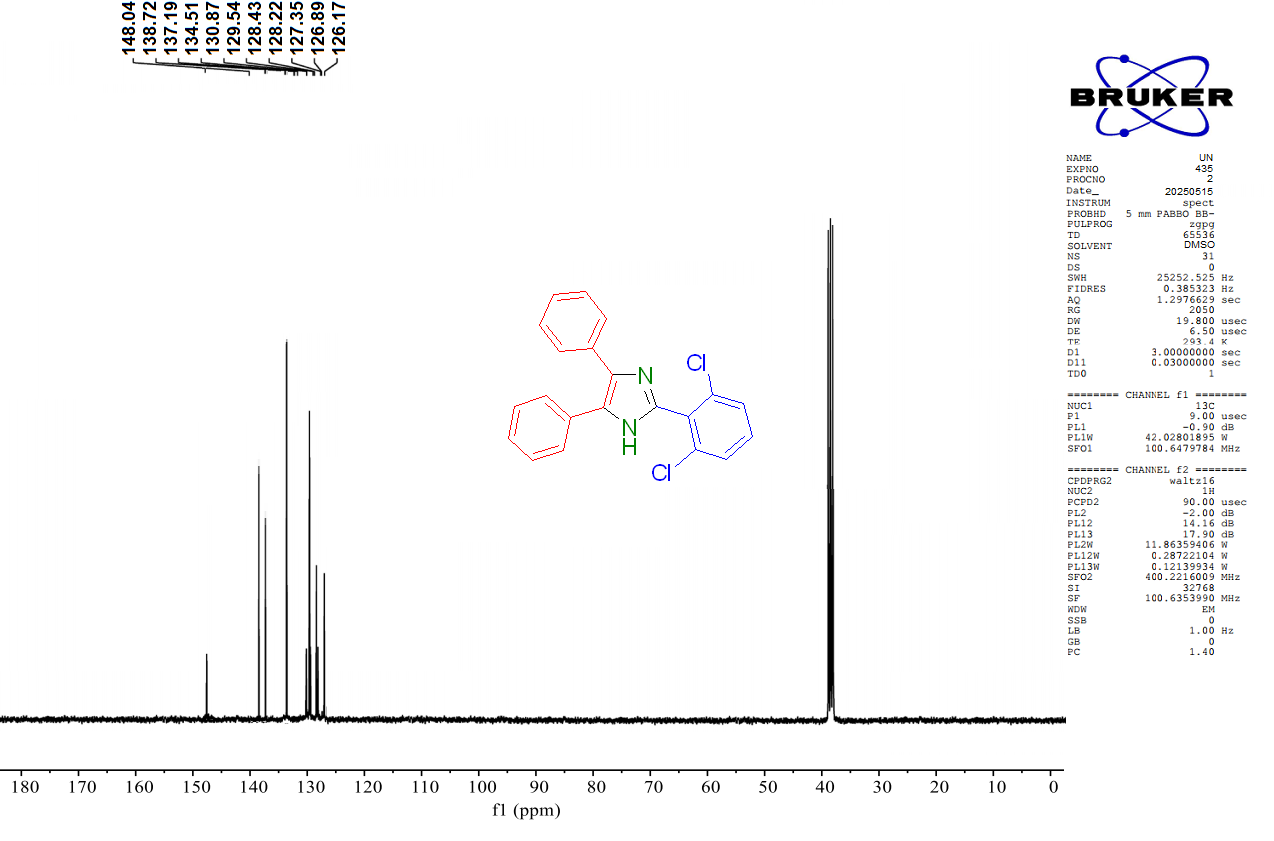


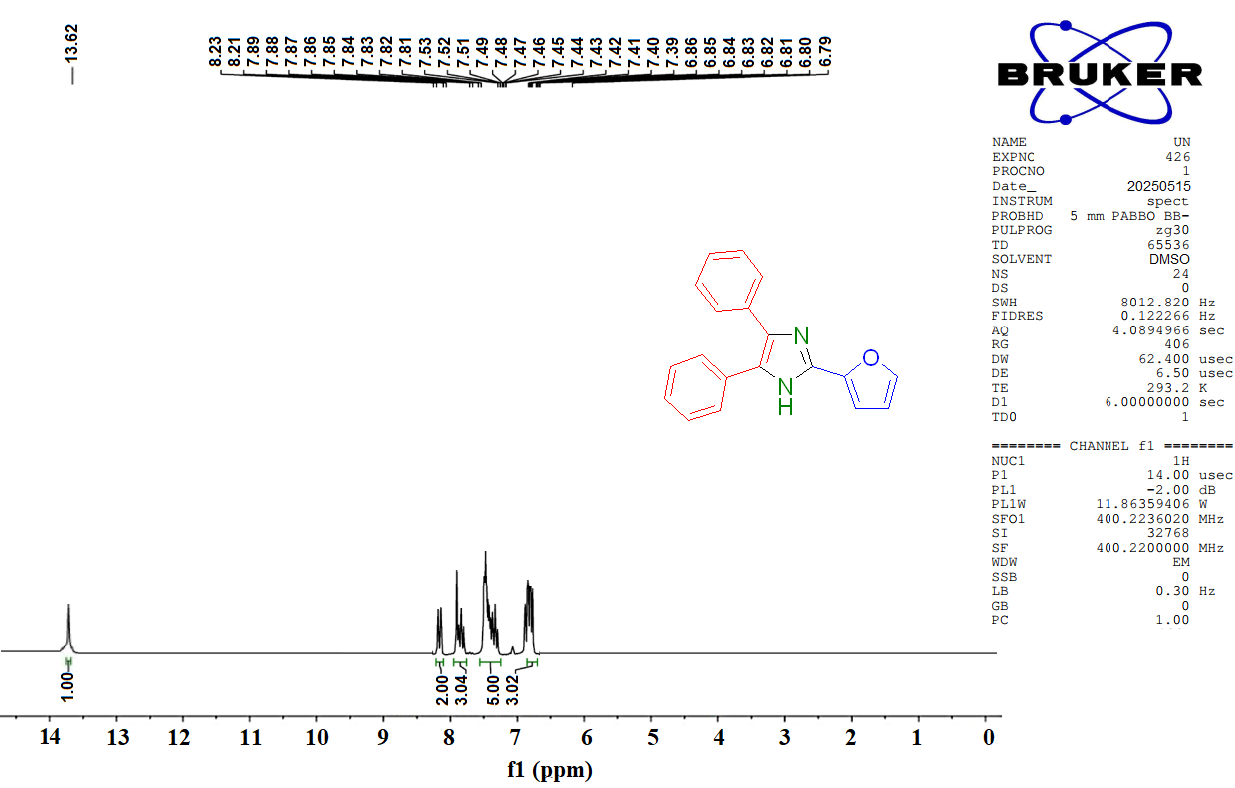


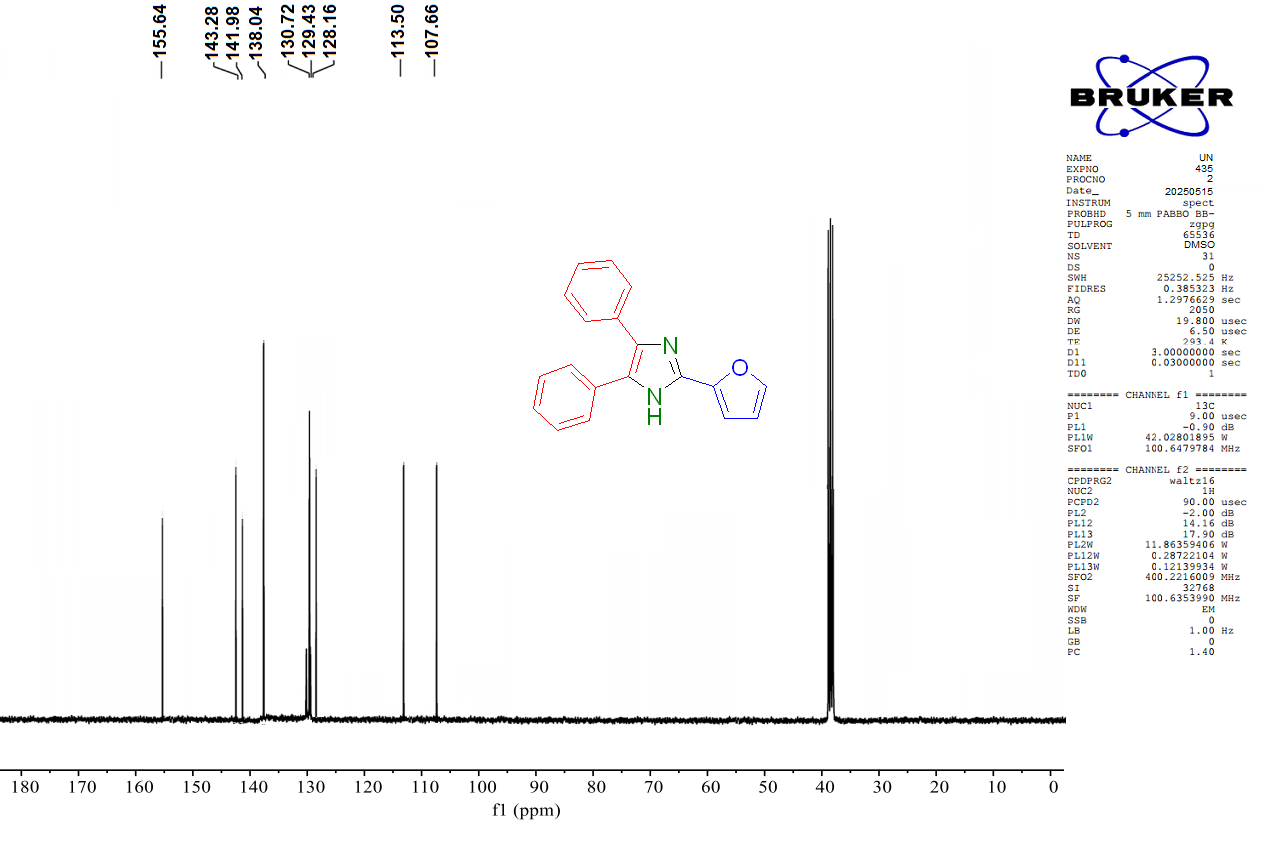


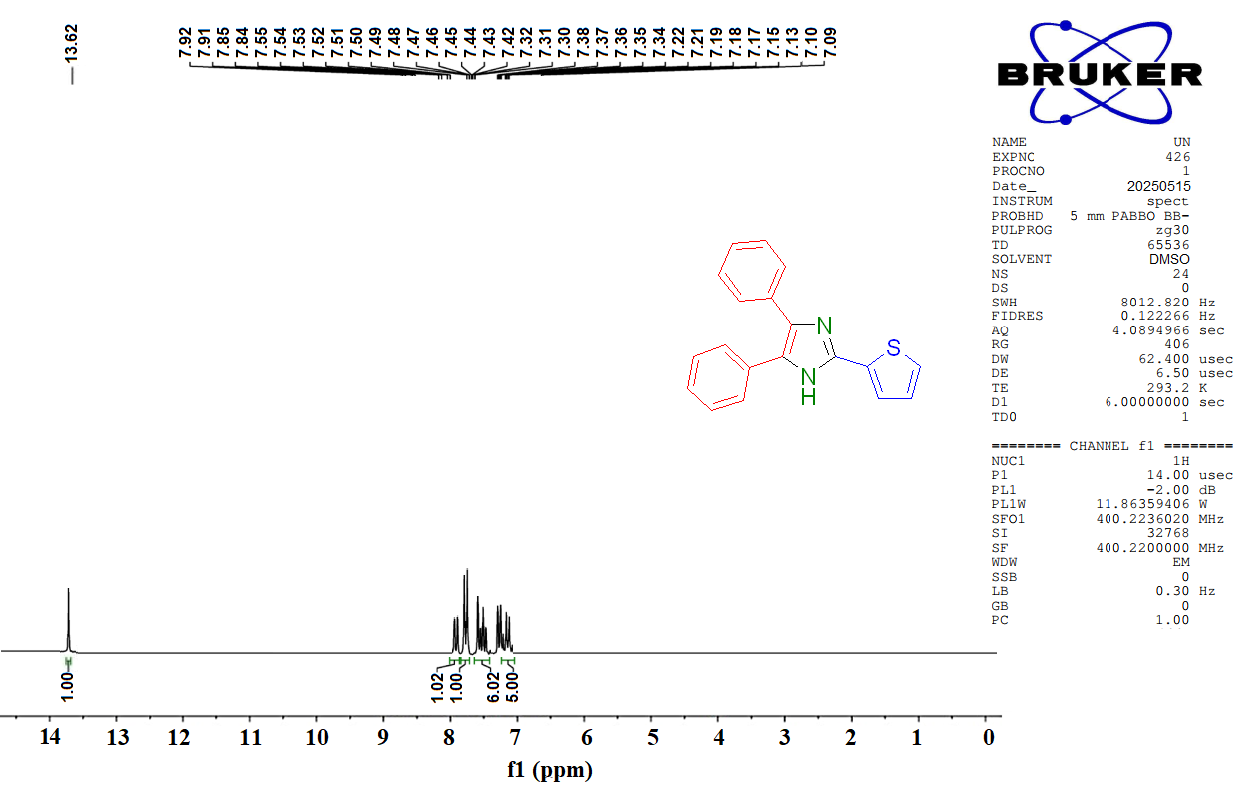


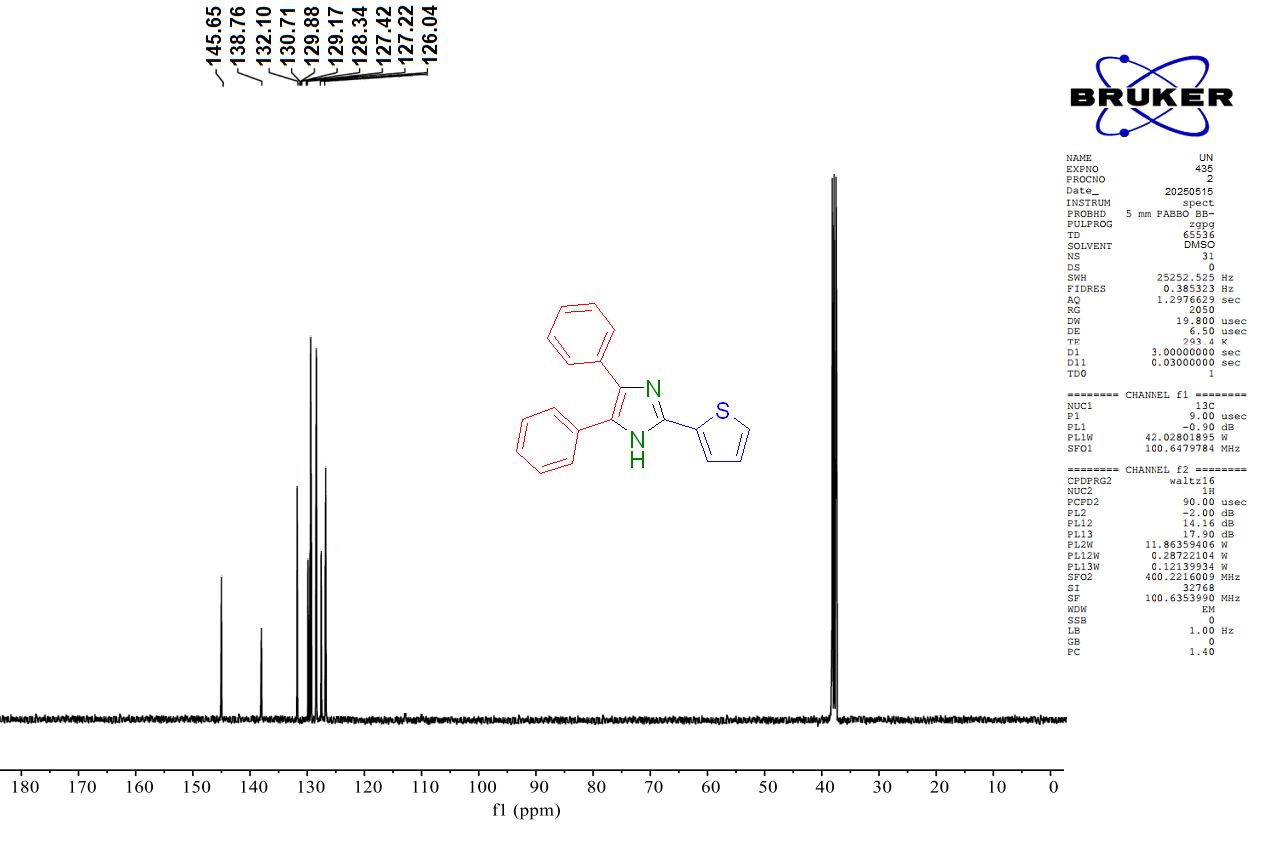


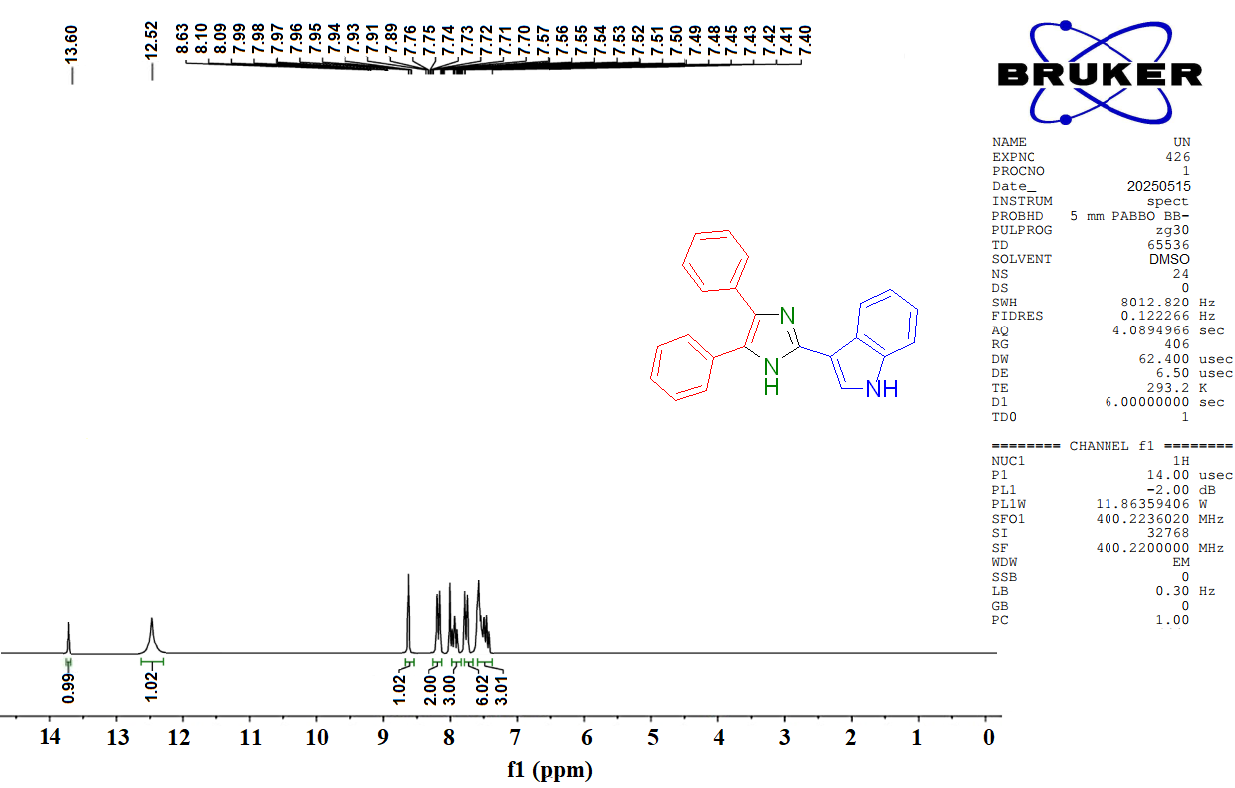


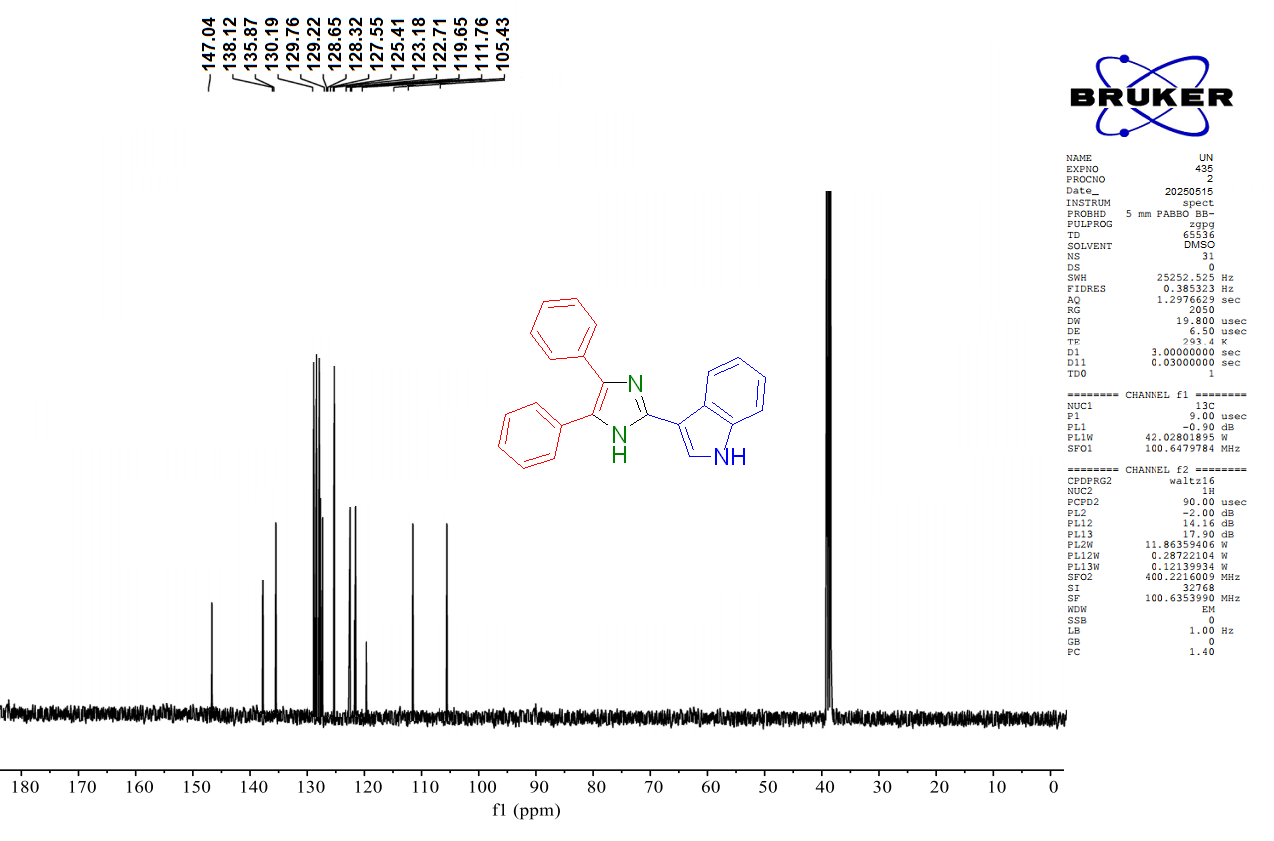


**NMR data for oxazole products**

**2,4,5-triphenyloxazole**

**(M.P: 111-113 ^o^C)**

^1^H NMR (400 MHz, DMSO) δ 8.34 (d, *J* = 7.6 Hz, 2H), 8.10 (d, *J* = 7.7 Hz, 2H), 7.91 (d, *J* = 8.3 Hz, 2H), 7.63-7.43 (m, 9H); ^13^C NMR (100 MHz, DMSO) δ 160.1, 156.9, 147.0, 130.5, 130.1, 129.7, 128.5, 128.2, 127.6, 127.4 ppm.

**4,5-diphenyl-2-(p-tolyl)oxazole**

**(M.P: 126-128 ^o^C)**

^1^H NMR (400 MHz, DMSO) δ 8.31 (d, *J* = 7.4 Hz, 2H), 8.02 (d, *J* = 8.0 Hz, 2H), 7.83 (d, *J* = 8.4 Hz, 2H), 7.58-7.39 (m, 6H), 7.16 (d, *J* = 7.7 Hz, 2H), 2.34 (s, 3H); ^13^C NMR (100 MHz, DMSO) δ 160.4, 146.3, 137.6, 132.0, 130.7, 129.5, 128.5, 127.1, 21.9 ppm.

**4-(4,5-diphenyloxazol-2-yl)benzonitrile**

**(M.P: 129-131 ^o^C)**

^1^H NMR (400 MHz, DMSO) δ 8.29 (d, *J* = 8.0 Hz, 2H), 8.03 (d, *J* = 7.6 Hz, 2H), 7.88 (d, *J* = 8.6 Hz, 4H), 7.55-7.38 (m, 6H); ^13^C NMR (100 MHz, DMSO) δ 160.2, 146.0, 137.4, 136.7, 133.5, 130.8, 129.7, 129.2, 128.1, 127.9, 127.3, 118.4, 113.8 ppm.

**2-(naphthalen-1-yl)-4,5-diphenyloxazole**

**(M.P: 125-127 ^o^C)**

^1^H NMR (400 MHz, DMSO) δ 8.96 (d, *J* = 7.3 Hz, 1H), 8.18 (d, *J* = 7.8 Hz, 1H), 8.03 (d, *J* = 8.0 Hz, 2H), 7.93 (d, *J* = 7.6 Hz, 3H), 7.81 (d, *J* = 7.7 Hz, 2H), 7.54-7.38 (m, 6H); ^13^C NMR (100 MHz, DMSO) δ 160.3, 146.7, 138.5, 137.1, 134.2, 130.1, 129.8, 129.2, 128.6, 128.3, 127.2, 126.9, 126.1, 125.4 ppm.

**4,5-diphenyl-2-(pyridin-3-yl)oxazole**

**(M.P: 105-107 ^o^C)**

^1^H NMR (400 MHz, DMSO) δ 9.33 (s, 1H), 8.75 (d, *J* = 9.0 Hz, 1H), 8.47 (d, *J* = 7.2 Hz, 1H), 8.03 (d, *J* = 8.0 Hz, 2H), 7.66 (d, *J* = 7.8 Hz, 2H), 7.47-7.29 (m, 6H); ^13^C NMR (100 MHz, DMSO) δ 159.8, 152.3, 146.7, 144.1, 137.6, 134.9, 130.2, 129.6, 129.1, 128.7, 128.4, 126.7, 125.1 ppm.

**4,5-diphenyl-2-(thiophen-2-yl)oxazole**

**(M.P: 113-115 ^o^C)**

^1^H NMR (400 MHz, DMSO) δ 8.13 (d, *J* = 8.6 Hz, 2H), 8.01 (d, *J* = 8.0 Hz, 2H), 7.90 (d, *J* = 7.6 Hz, 2H), 7.64-7.45 (m, 6H), 7.21 (t, *J* = 7.7 Hz, 1H); ^13^C NMR (100 MHz, DMSO) δ 153.2, 147.8, 136.9, 131.7, 129.4, 128.6, 128.1, 127.6 ppm.

**2,4,5-tris(4-chlorophenyl)oxazole**

**(M.P: 144-146 ^o^C)**

^1^H NMR (400 MHz, DMSO) δ 7.94 (d, *J* = 7.6 Hz, 2H), 7.57 (d, *J* = 7.4 Hz, 4H), 7.41 (d, *J* = 8.0 Hz, 2H), 7.18 (d, *J* = 7.7 Hz, 4H); ^13^C NMR (100 MHz, DMSO) δ 159.9, 145.1, 138.6, 136.0, 132.8, 129.7, 129.1, 128.4, 128.2, 127.6, 123.8, 122.1 ppm.

**4,5-bis(4-(tert-butyl)phenyl)-2-phenyloxazole**

**(M.P: 151-153 ^o^C)**

^1^H NMR (400 MHz, DMSO) δ 8.27 (d, *J* = 7.3 Hz, 2H), 7.84 (d, *J* = 7.8 Hz, 2H), 7.72-7.54 (m, 3H), 7.43 (d, *J* = 8.0 Hz, 2H), 7.32 (d, *J* = 7.6 Hz, 4H), 1.38 (s, 18H); ^13^C NMR (100 MHz, DMSO) δ 160.4, 153.1, 146.9, 138.6, 133.5, 133.0, 132.1, 129.7, 129.5, 128.7, 128.0, 127.4, 34.6, 30.8 ppm.

**4,5-bis(3-chlorophenyl)-2-phenyloxazole**

**(M.P: 116-118 ^o^C)**

^1^H NMR (400 MHz, DMSO) δ 8.12 (d, *J* = 8.0 Hz, 2H), 7.93 (s, 2H), 7.81 (d, *J* = 7.7 Hz, 2H), 7.52-7.30 (m, 7H); ^13^C NMR (100 MHz, DMSO) δ 160.5, 146.7, 137.2, 134.5, 132.9, 130.7, 127.6, 126.4, 125.8, 124.3, 123.4, 122.5, 121.0 ppm.

**2-phenyl-4,5-di(pyridin-3-yl)oxazole**

**(M.P: 131-133 ^o^C)**

^1^H NMR (400 MHz, DMSO) δ 9.28 (s, 2H), 8.69 (d, *J* = 7.7 Hz, 2H), 8.46 (d, *J* = 7.6 Hz, 2H), 8.19 (d, *J* = 8.0 Hz, 2H), 7.74-7.51 (m, 5H); ^13^C NMR (100 MHz, DMSO) δ 160.3, 147.8, 146.9, 145.0, 132.1, 132.0, 129.7, 127.6, 126.4, 125.8, 125.2, 122.3 ppm.

**4,5-di(furan-2-yl)-2-phenyloxazole**

**(M.P: 102-104 ^o^C)**

^1^H NMR (400 MHz, DMSO) δ 8.29 (d, *J* = 8.6 Hz, 2H), 8.01 (d, *J* = 7.7 Hz, 2H), 7.69 (d, *J* = 8.0 Hz, 1H), 7.55-7.37 (m, 3H), 7.31 (d, *J* = 7.8 Hz, 1H), 7.08 (t, *J* = 8.4 Hz, 1H), 6.64 (t, *J* = 8.6 Hz, 1H); ^13^C NMR (100 MHz, DMSO) δ 160.1, 158.7, 153.0, 144.3, 137.6, 132.5, 132.1, 131.4, 130.7, 129.8, 128.1, 127.6, 125.4, 124.9, 113.7, 108.3 ppm.


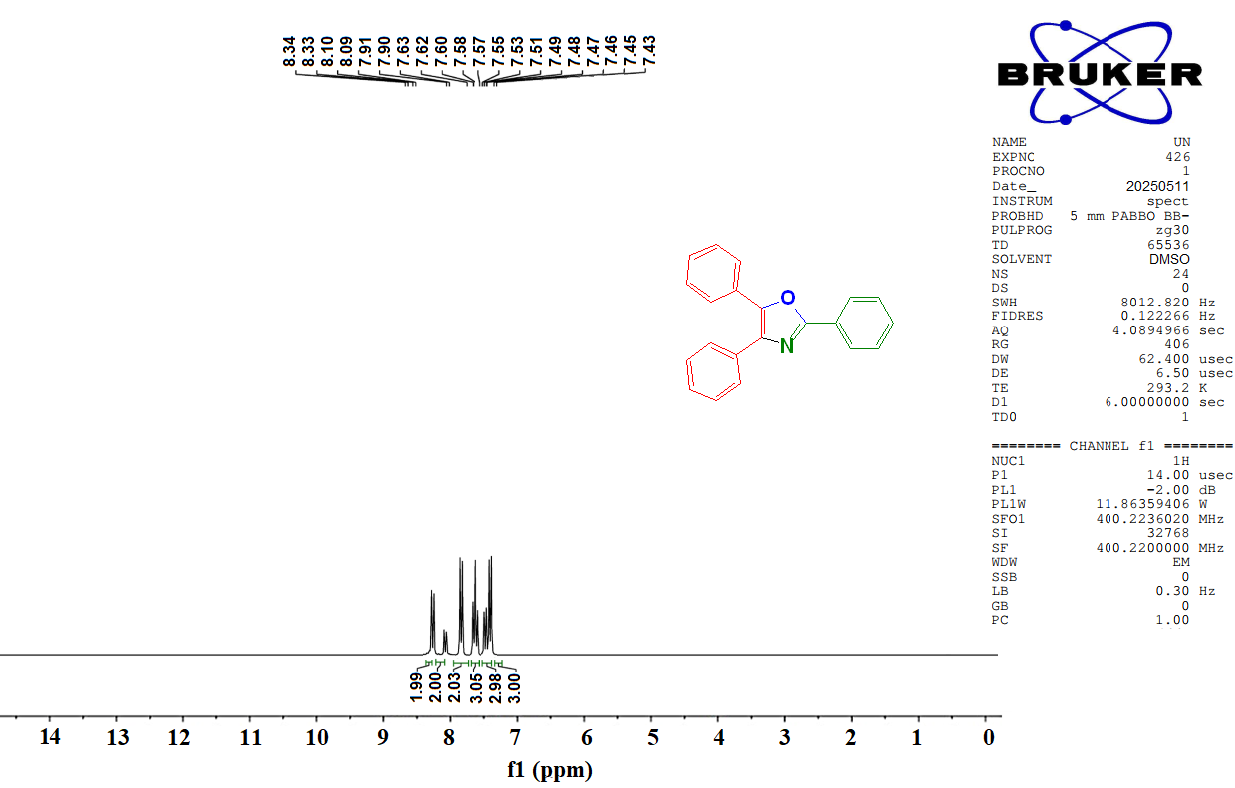


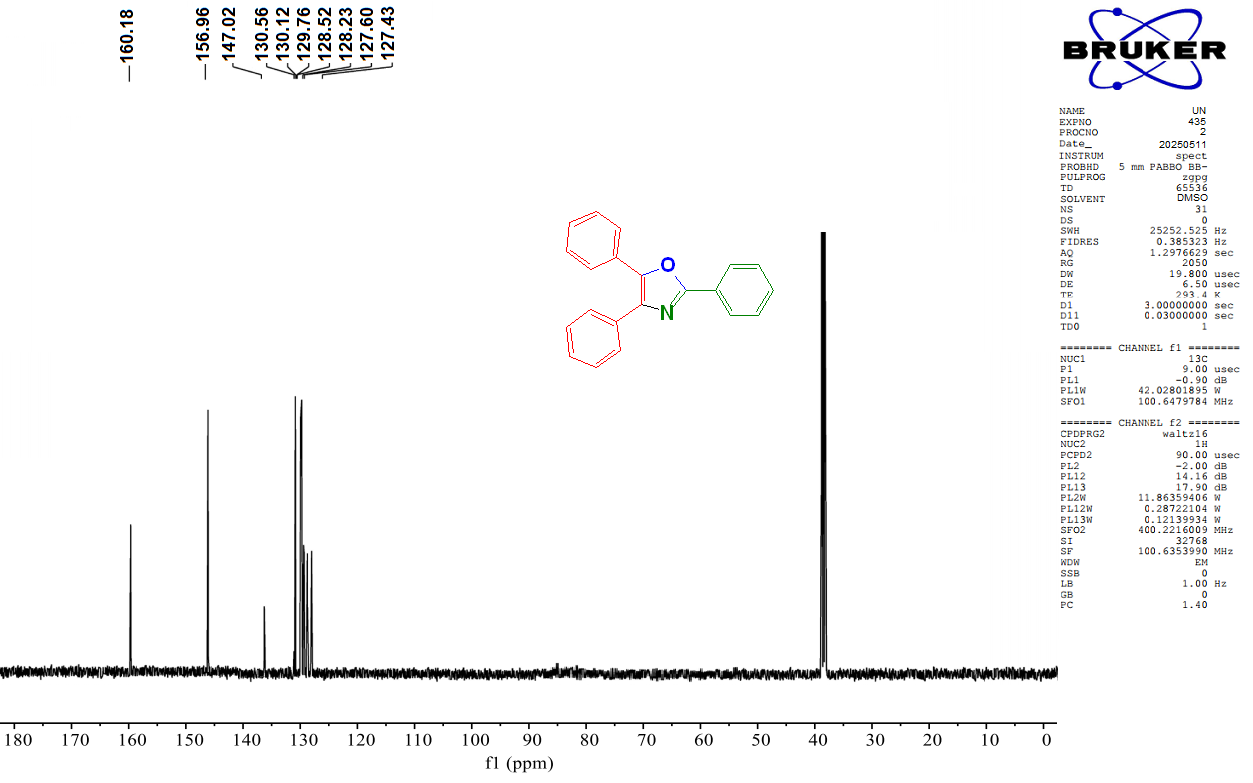


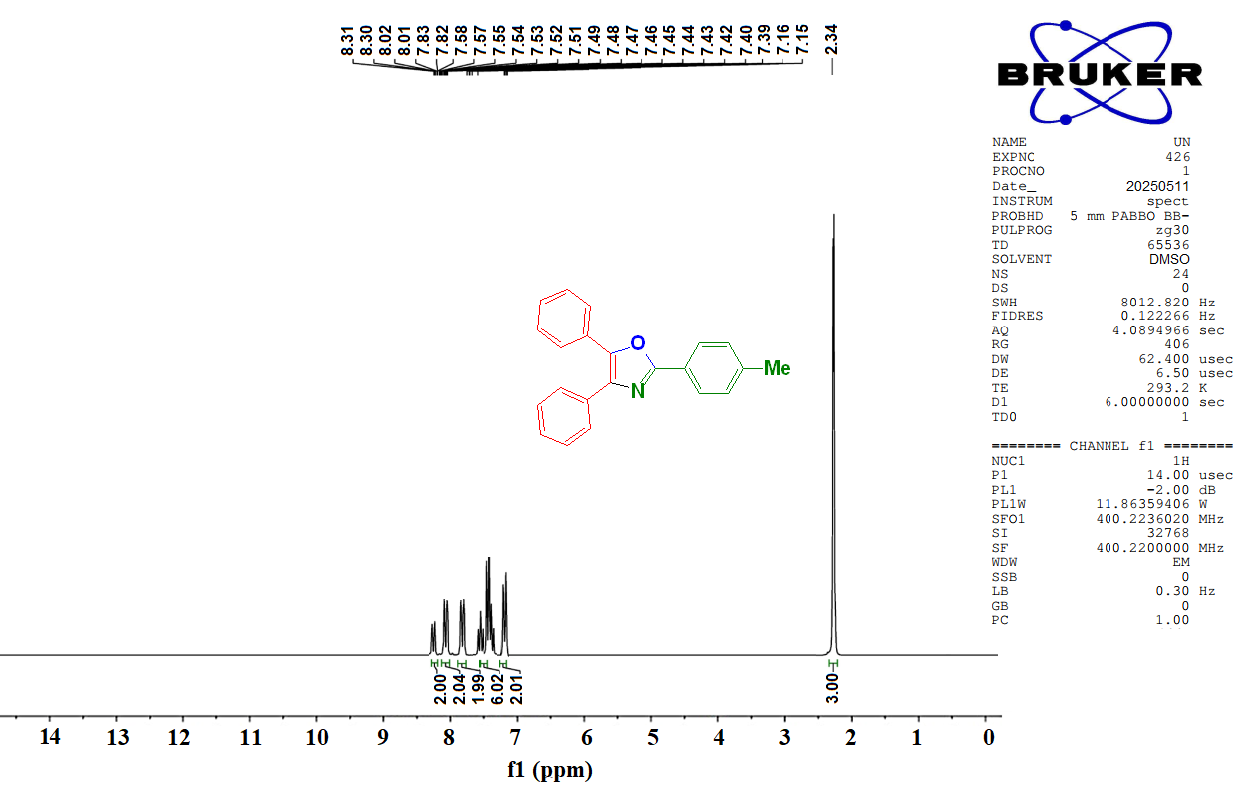


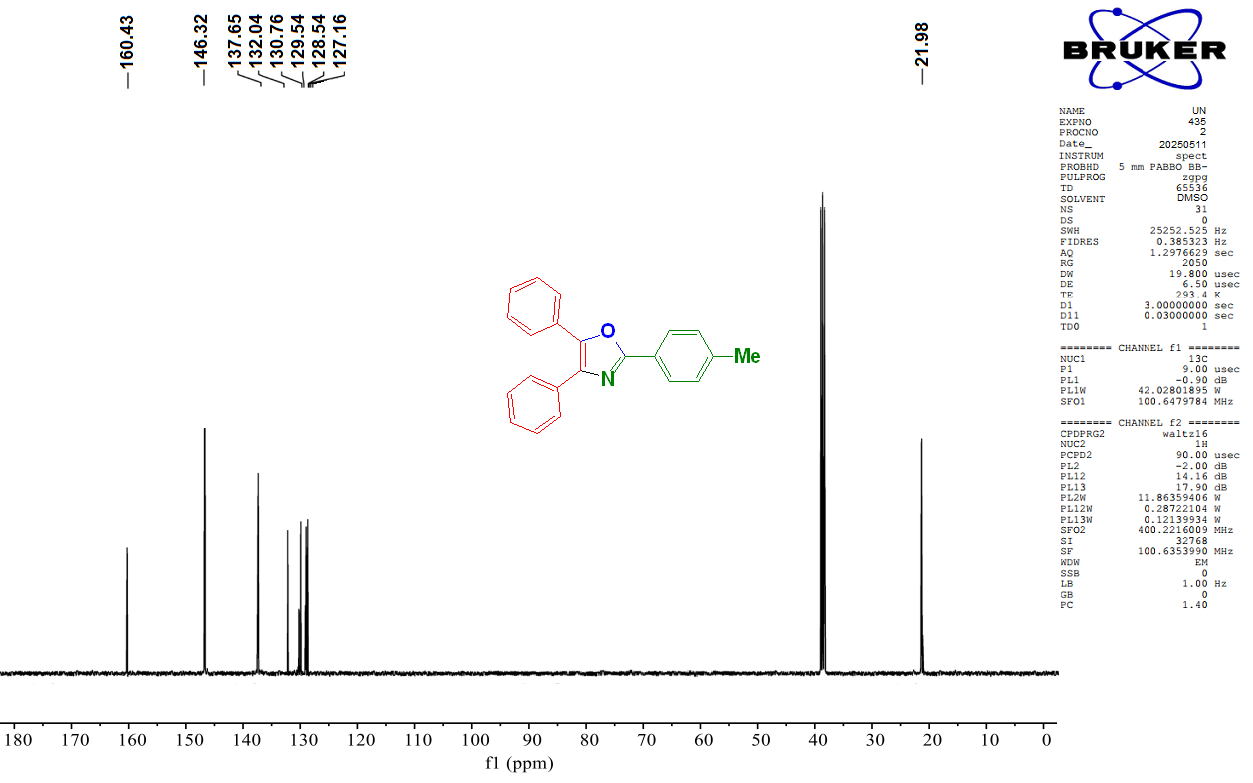


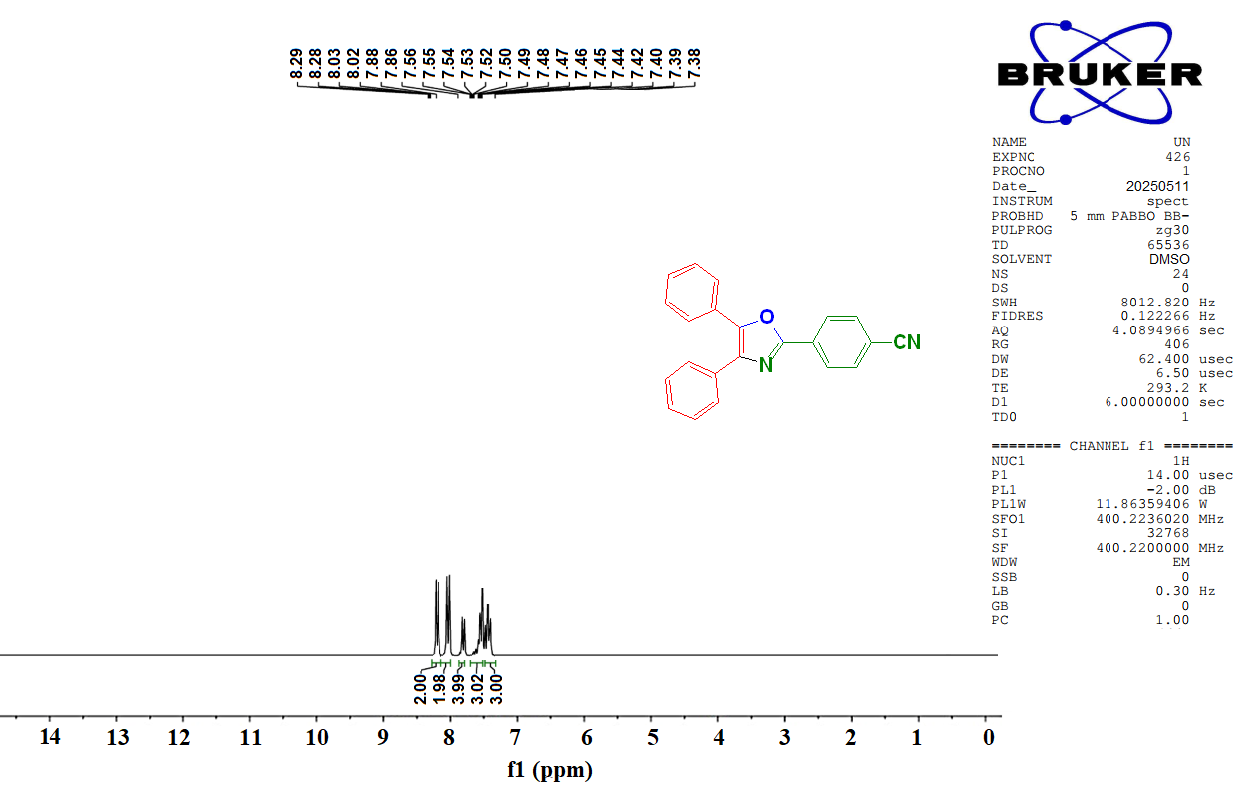


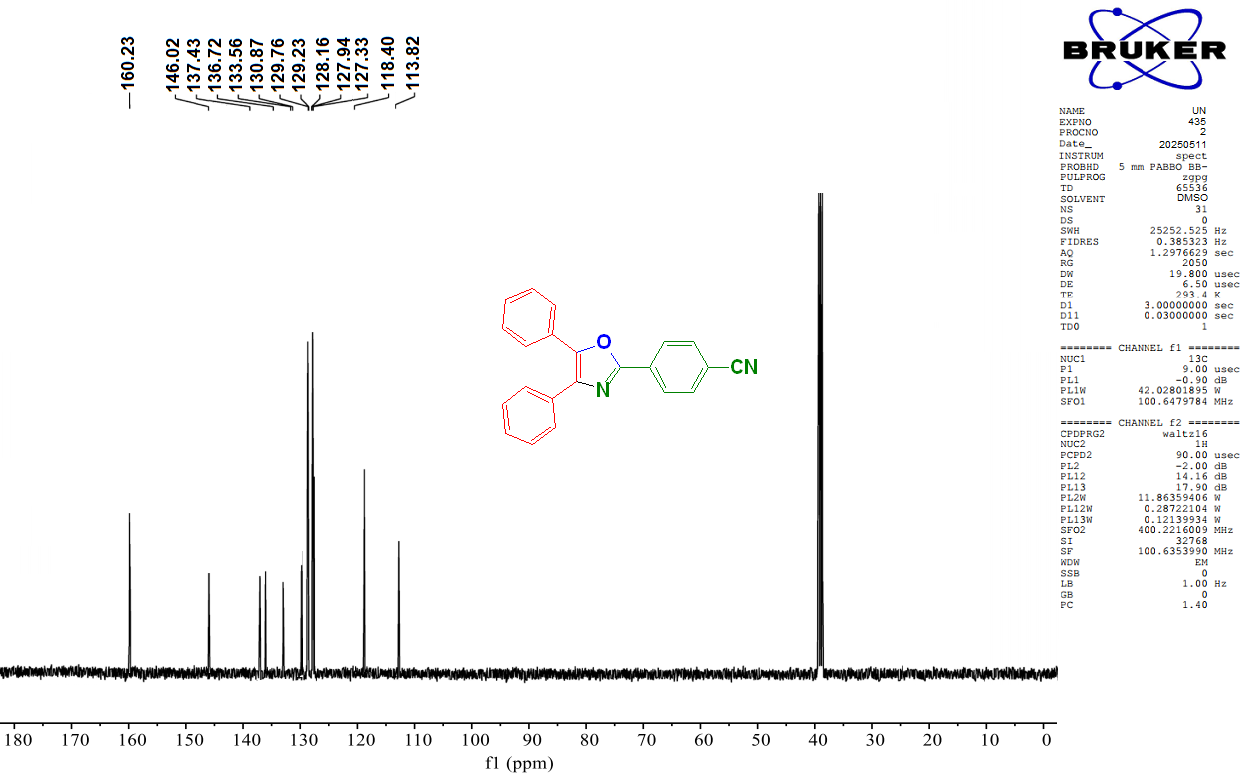


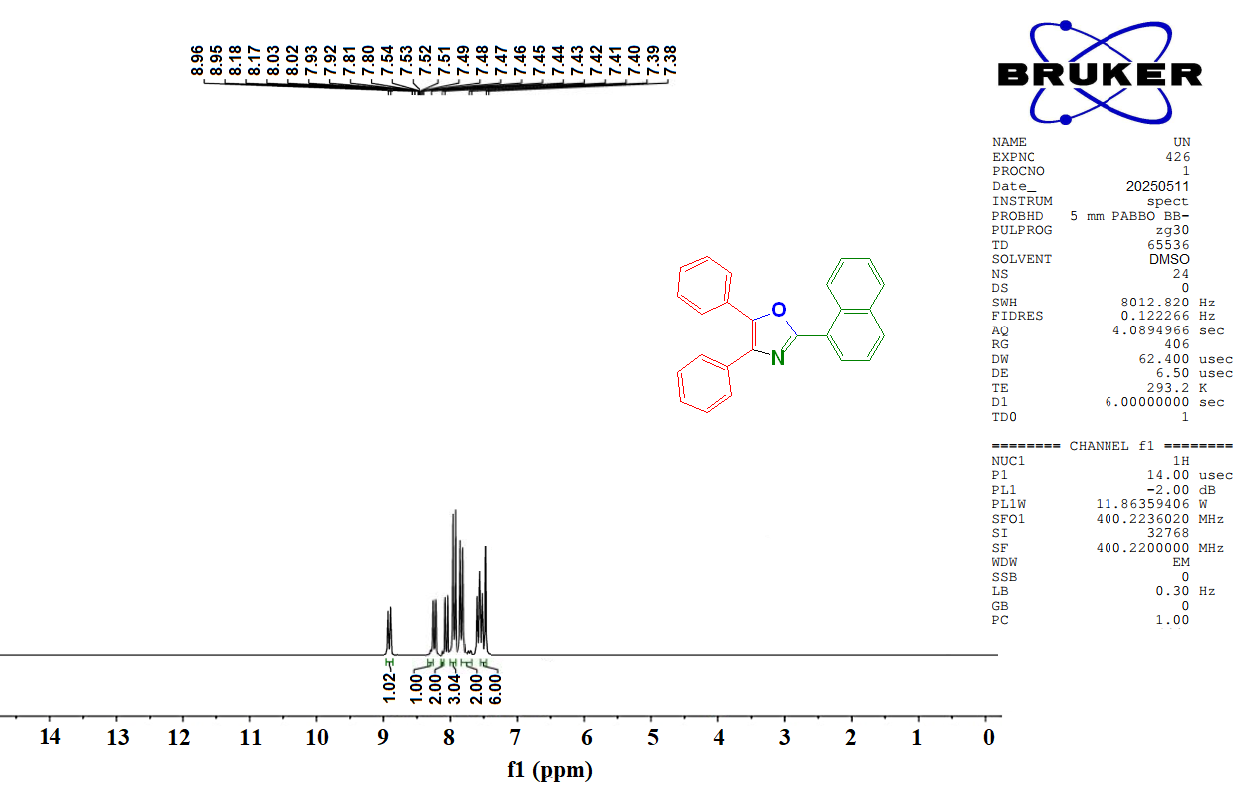


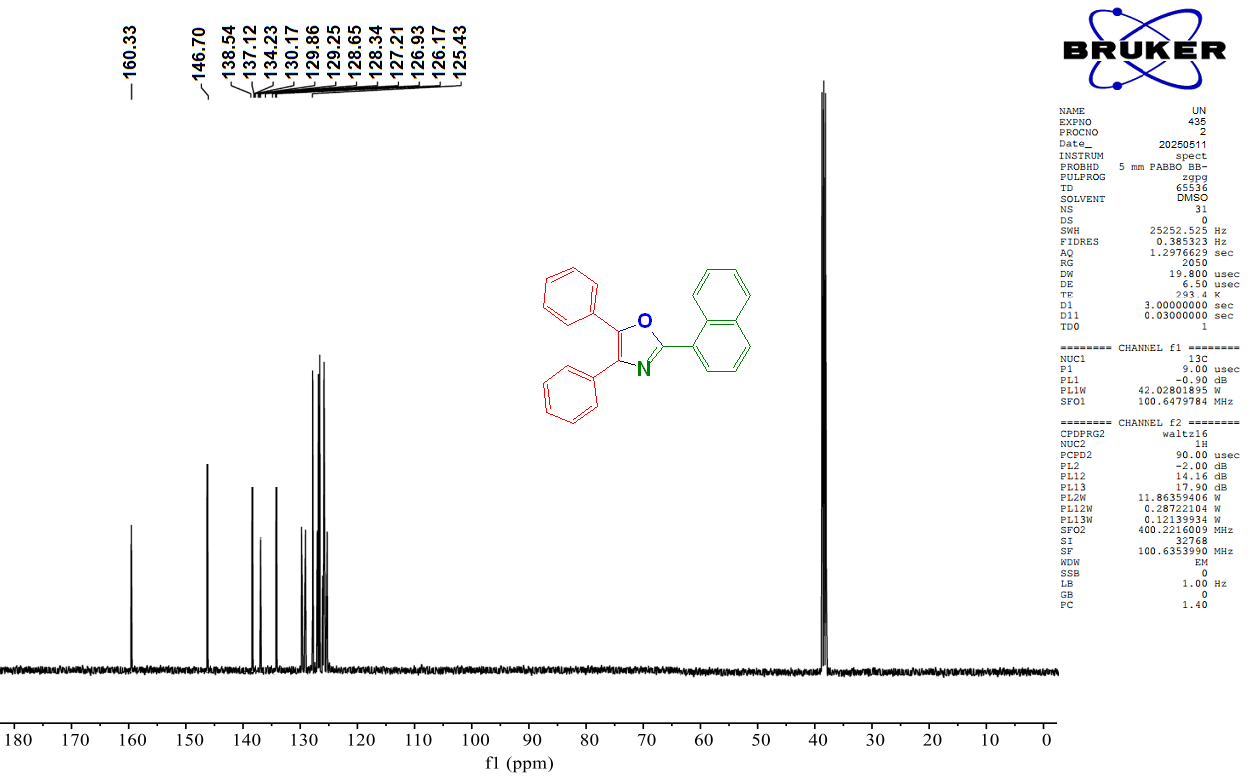


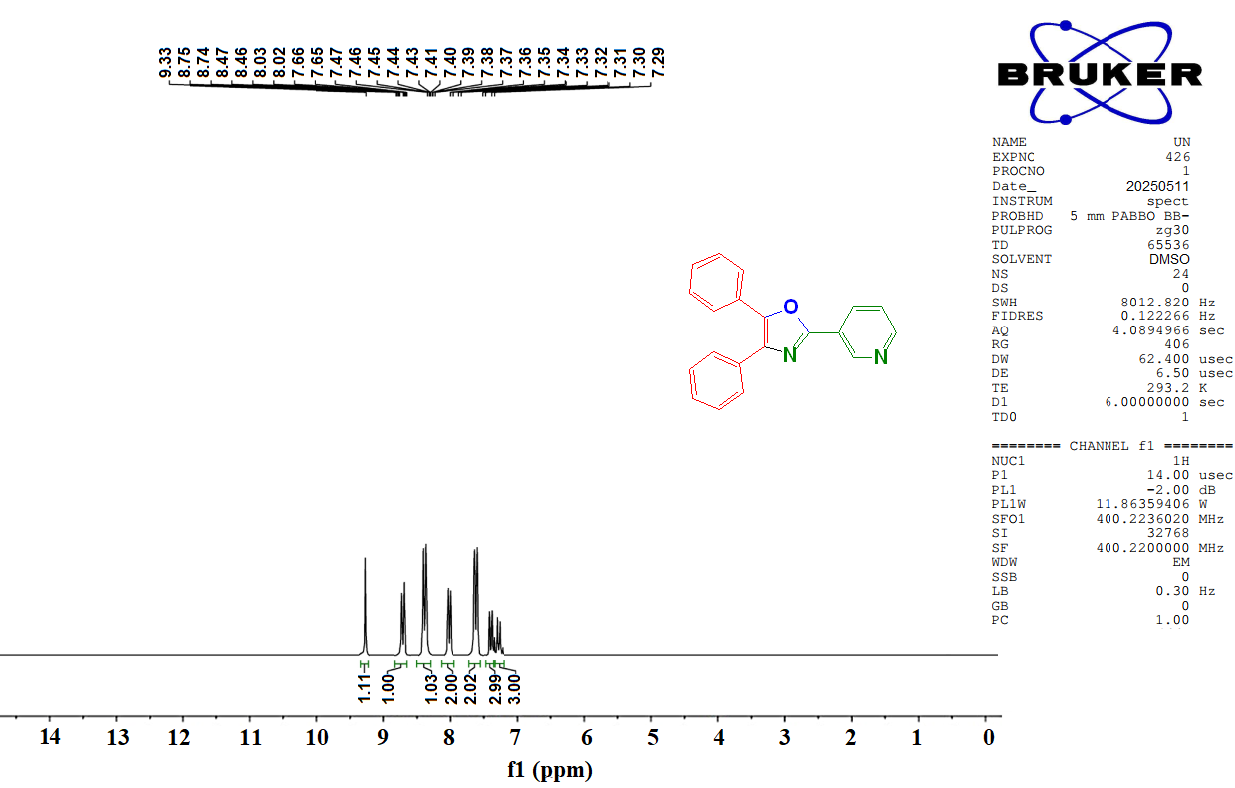


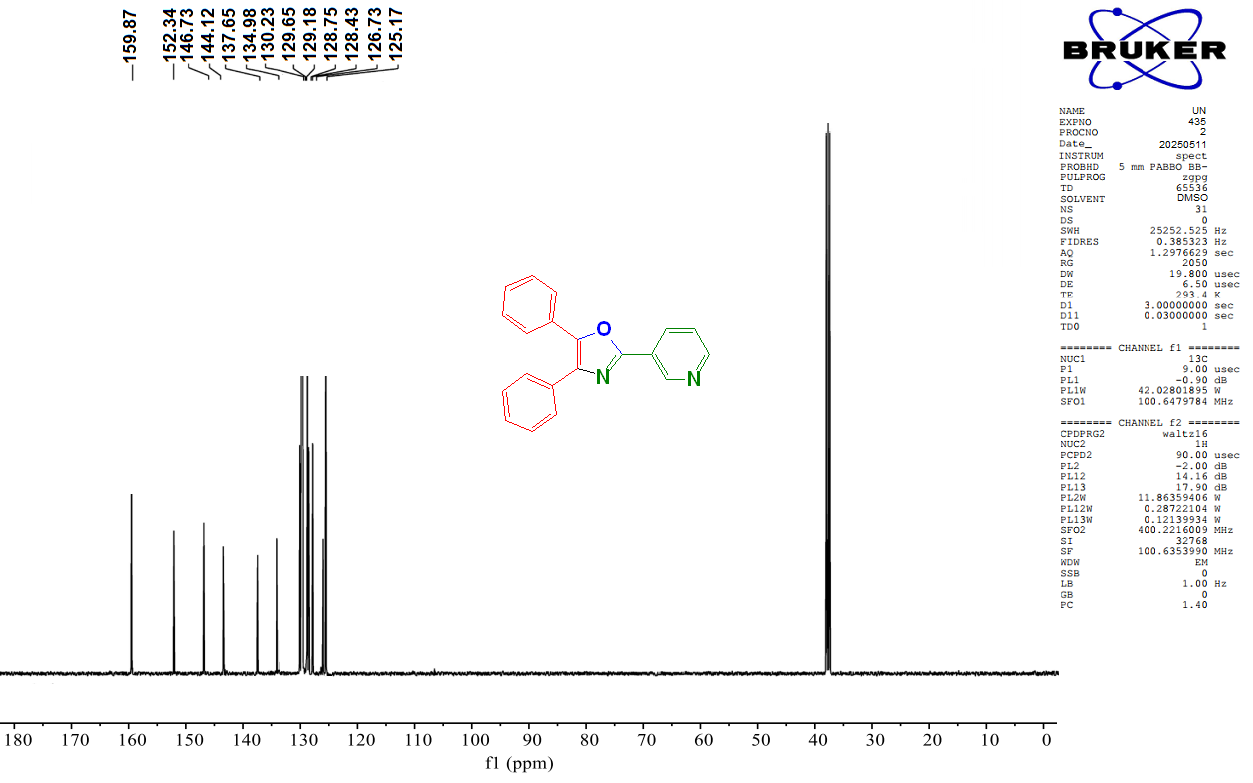


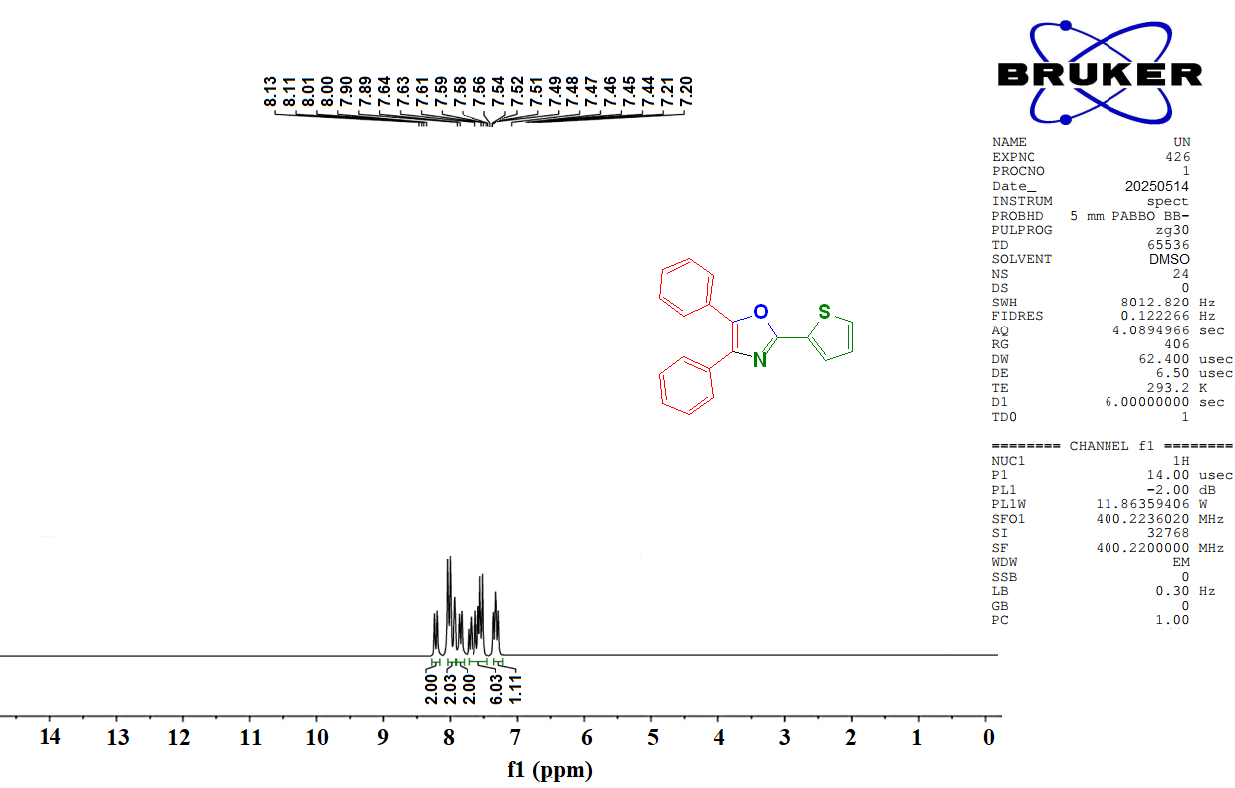

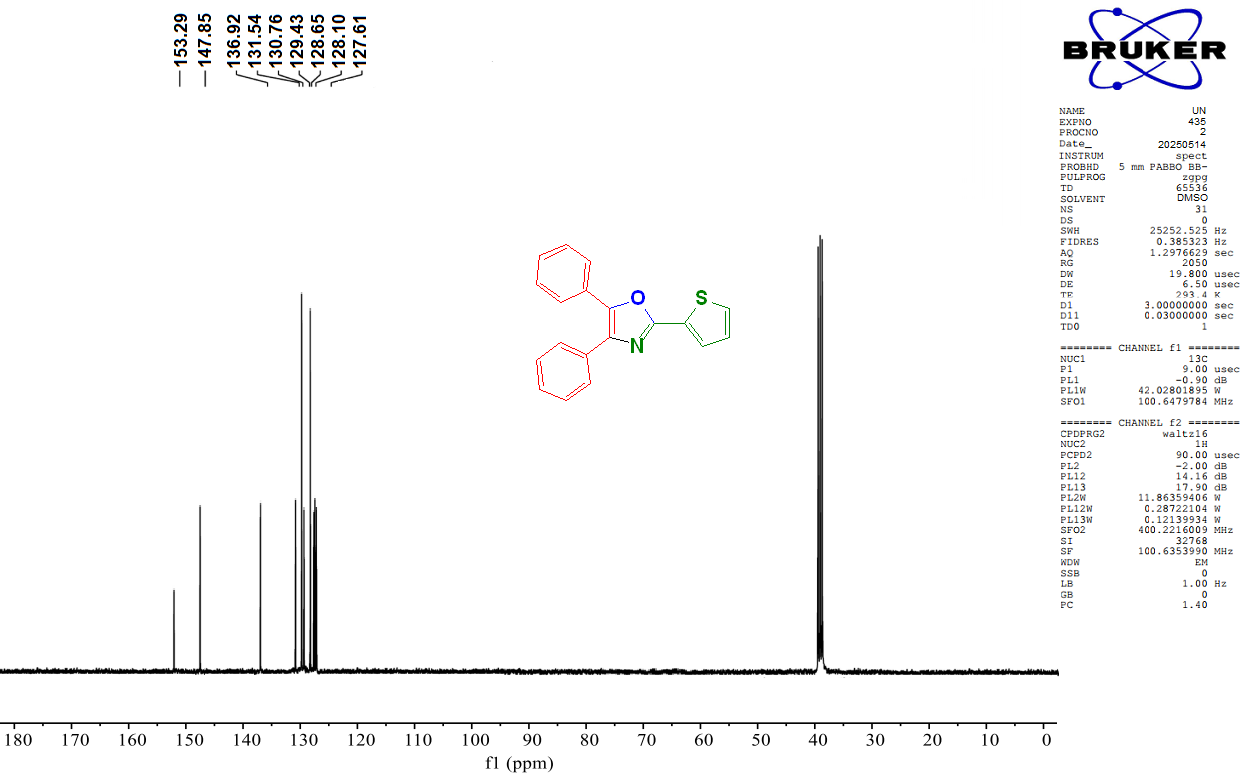


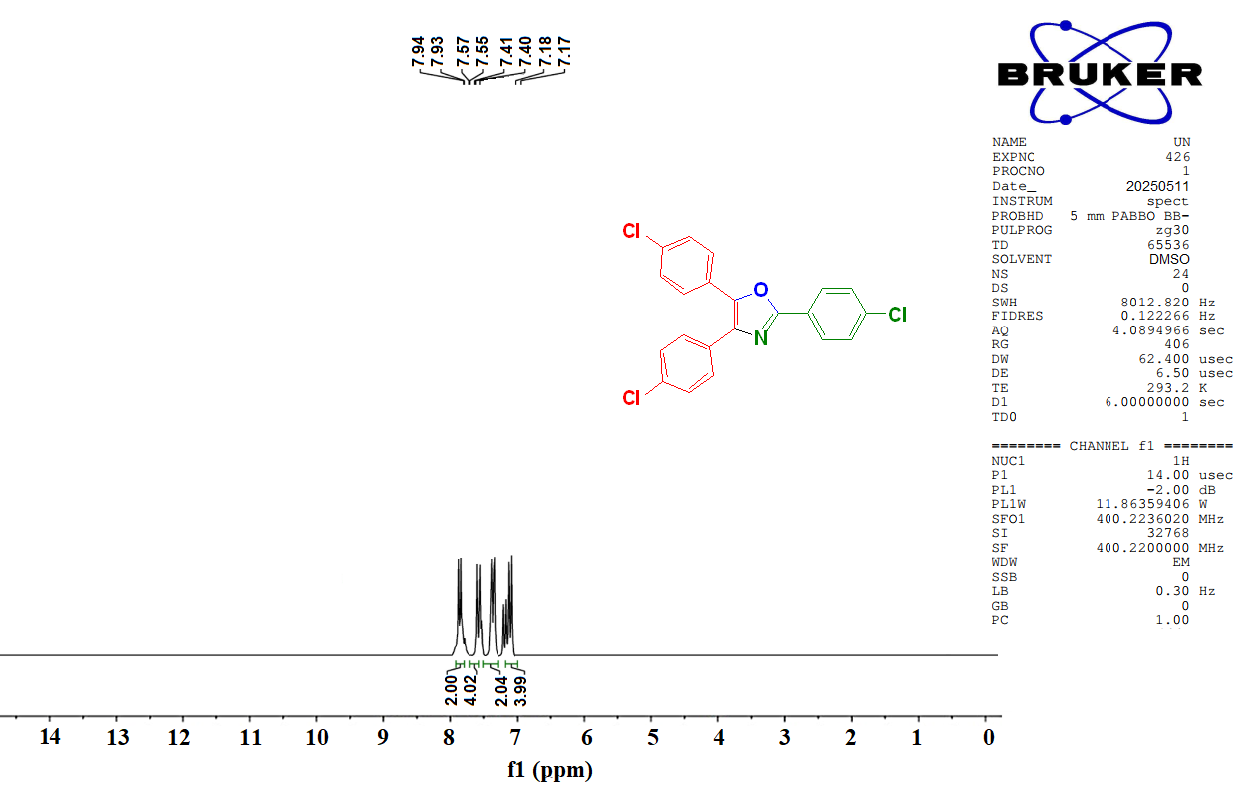


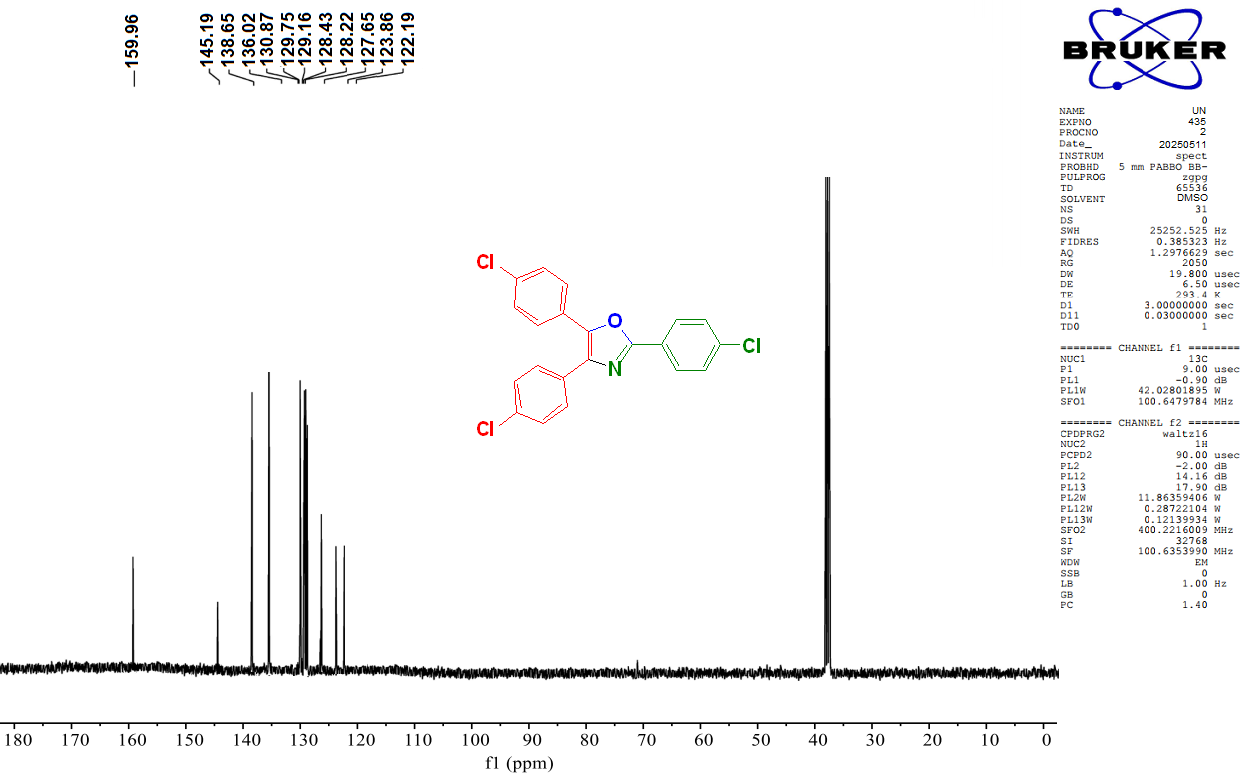


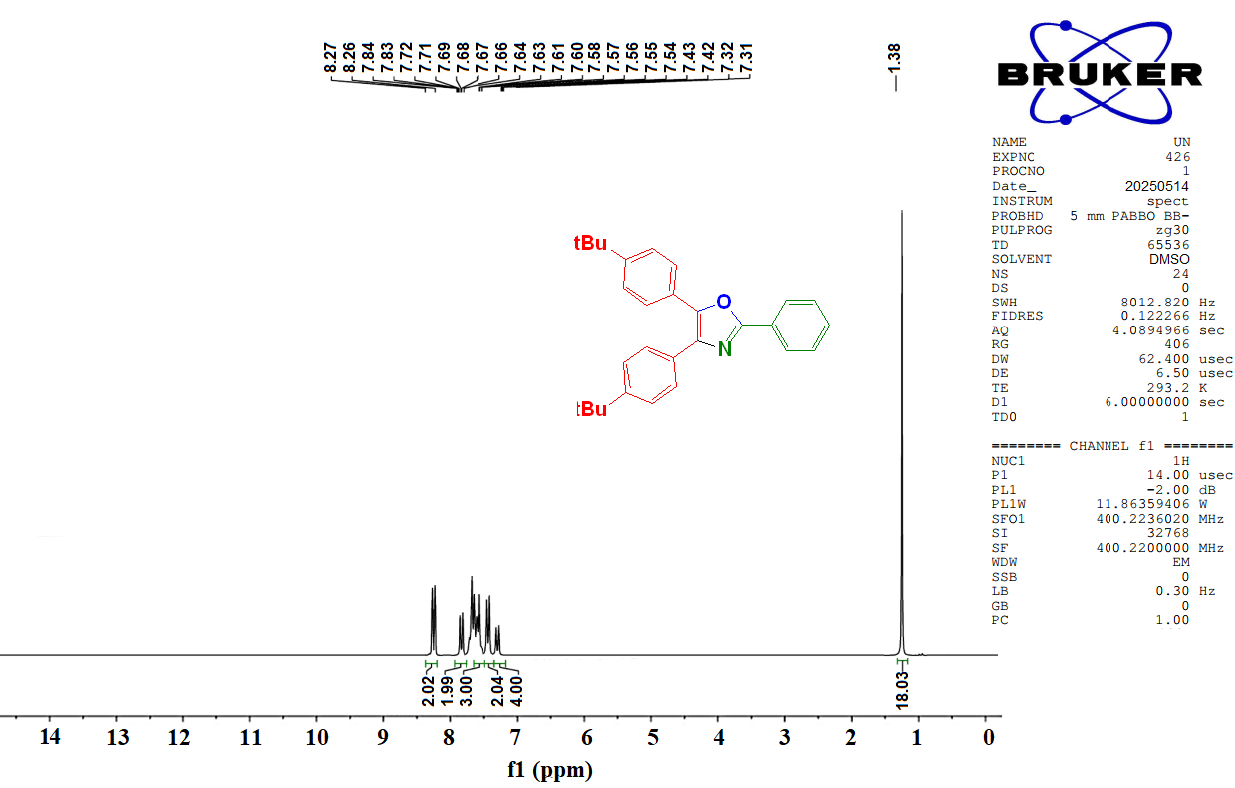


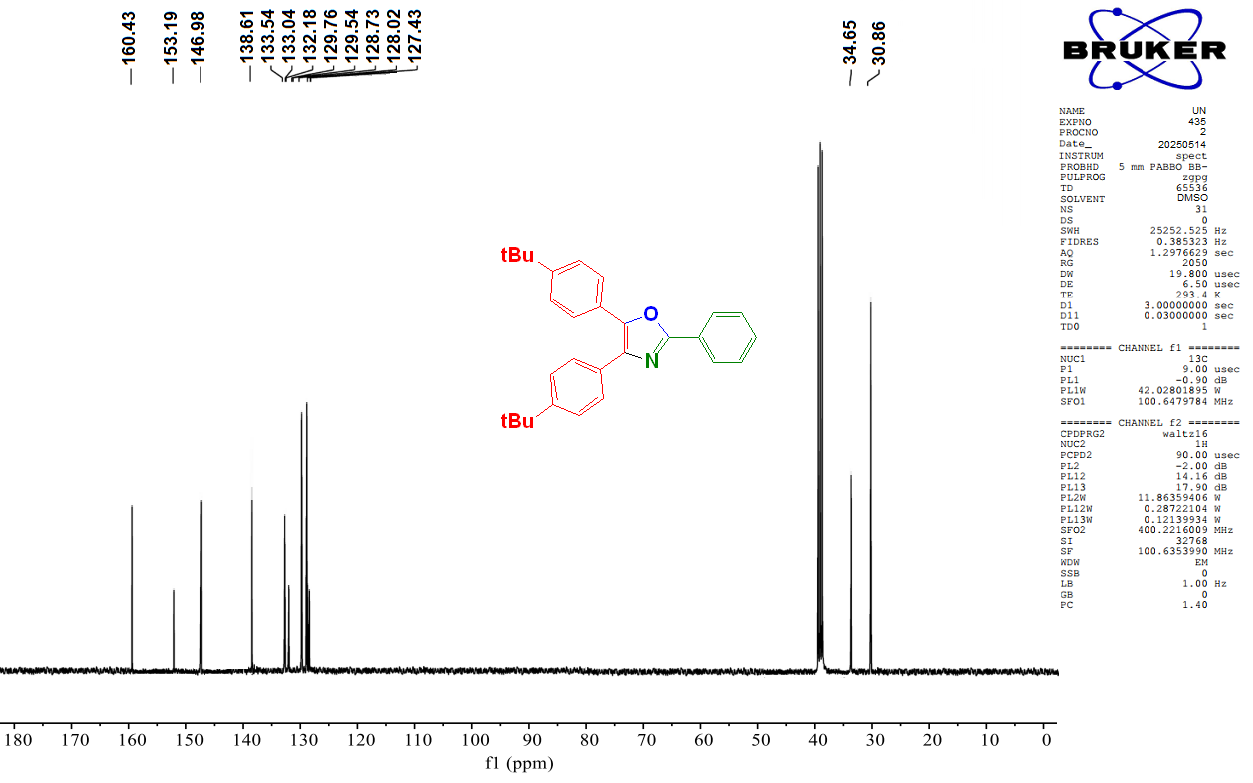


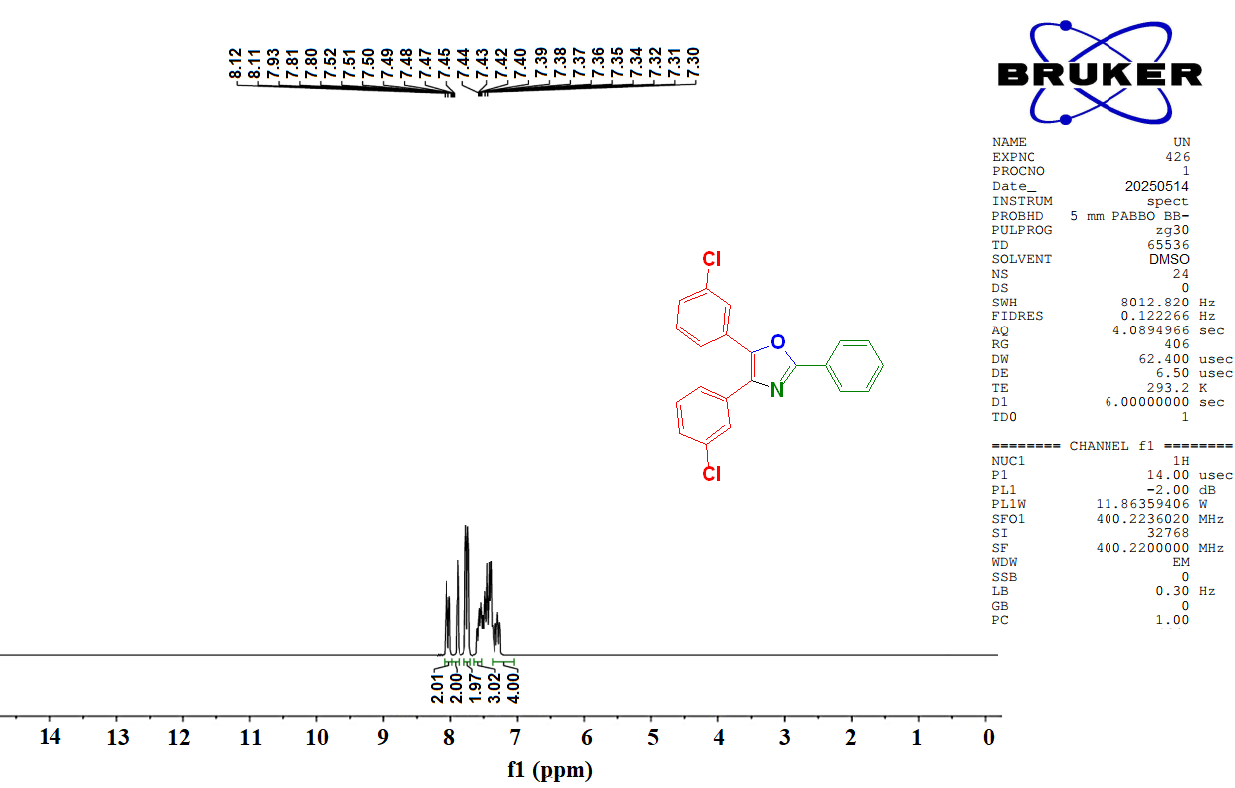


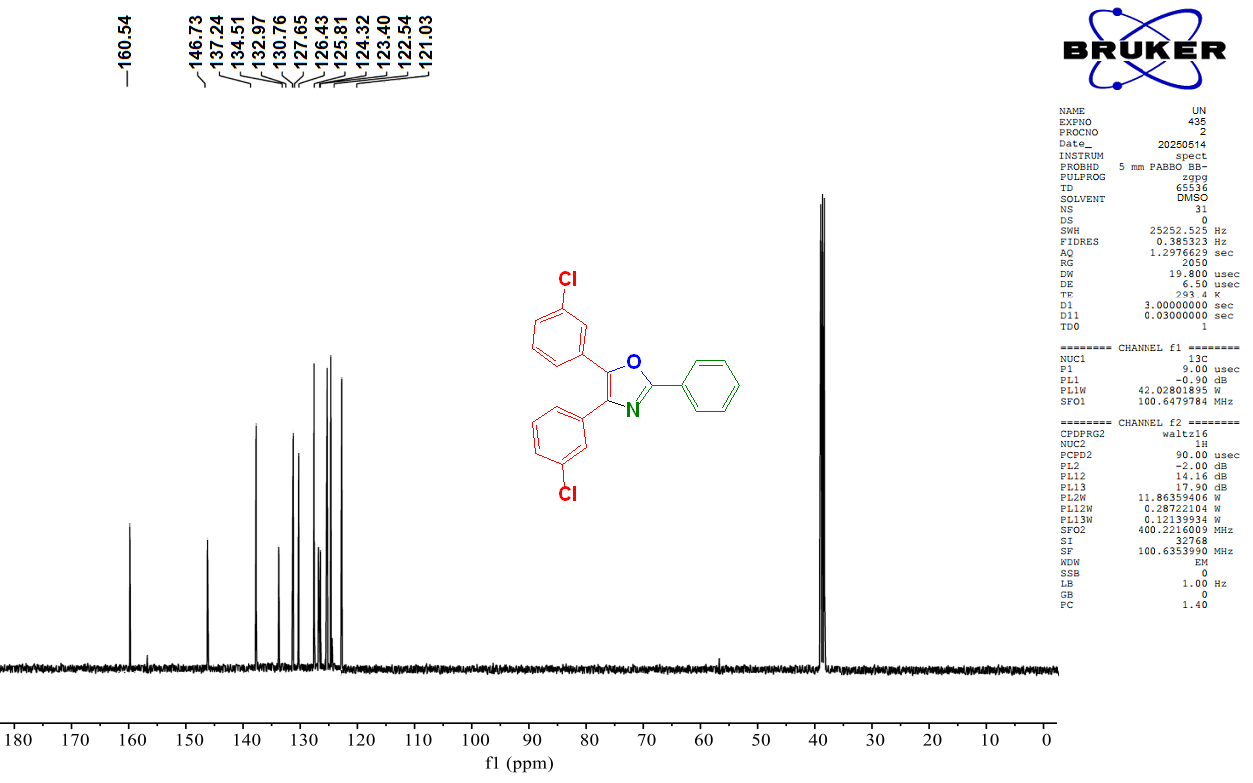


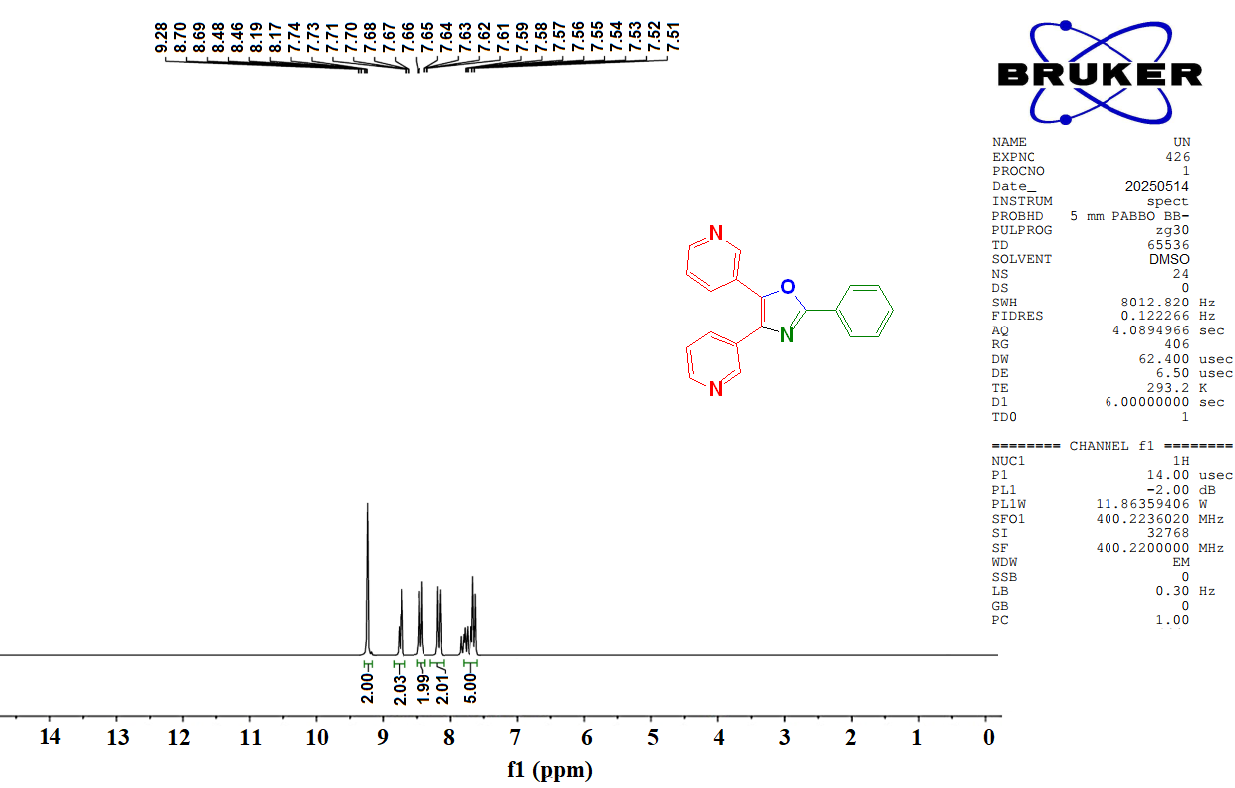


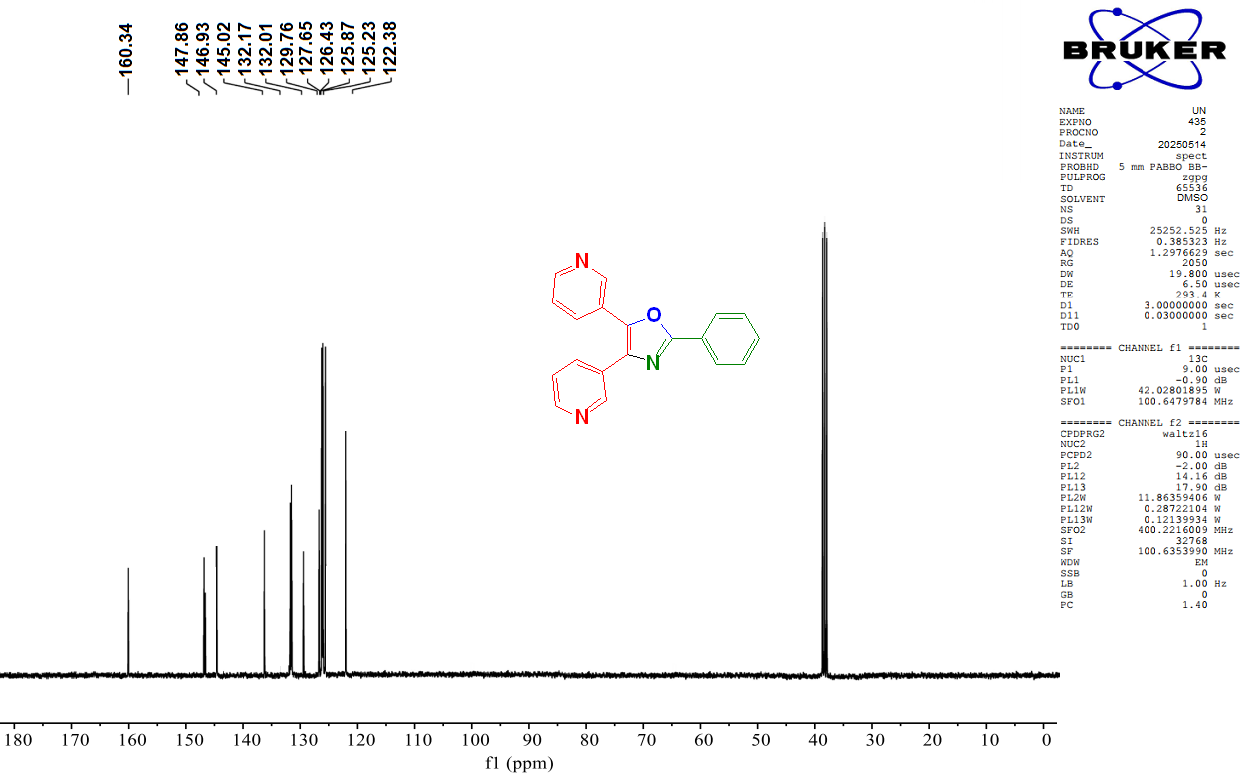


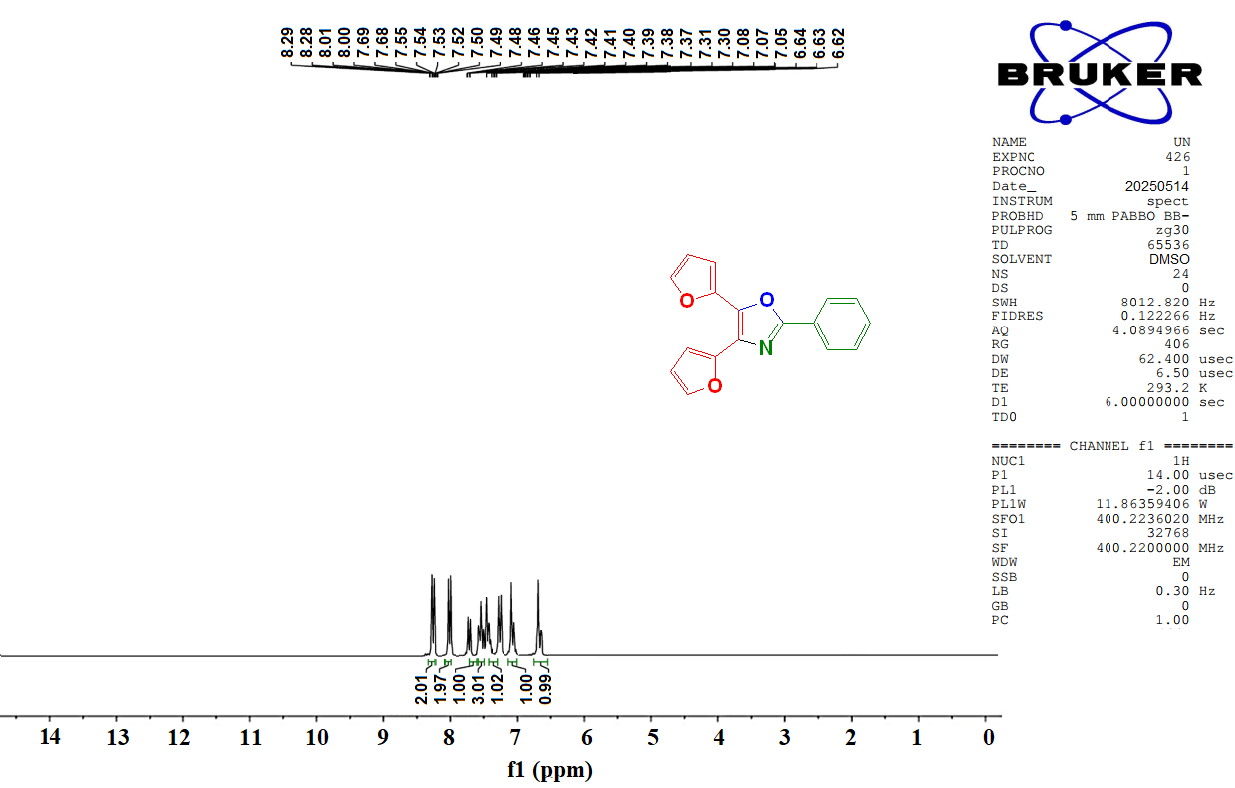


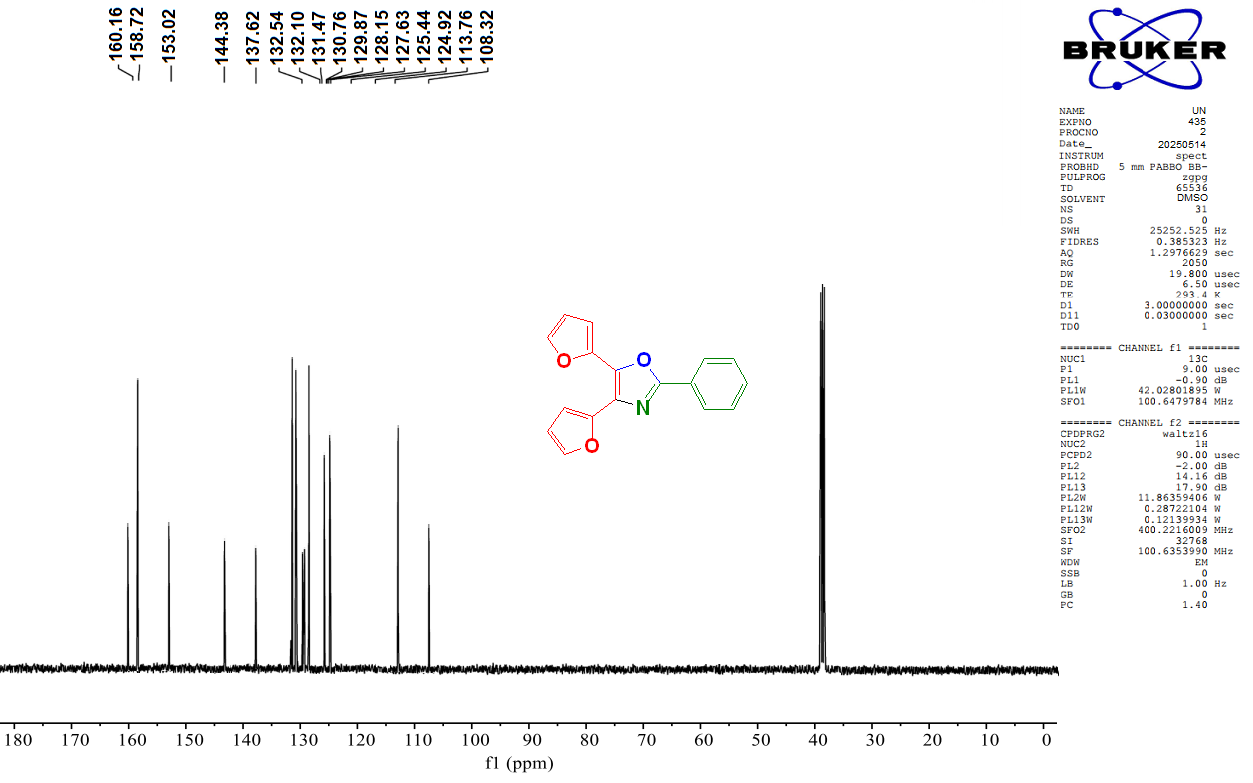


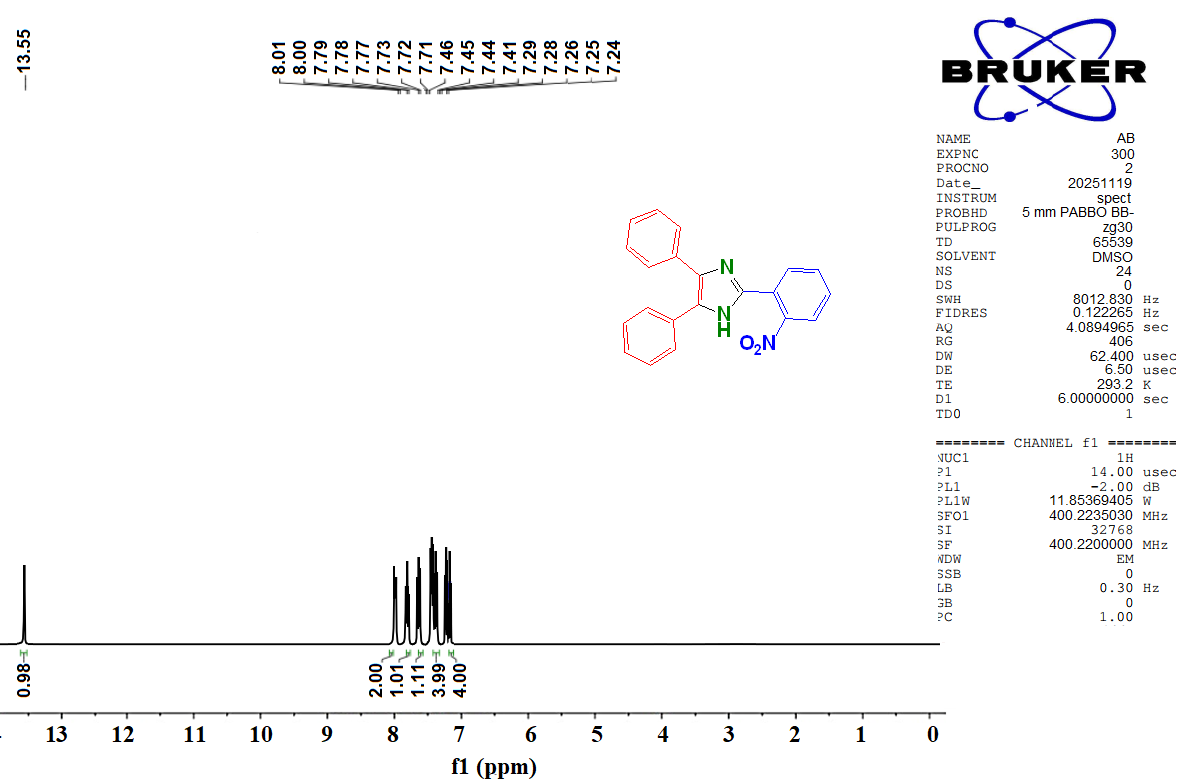


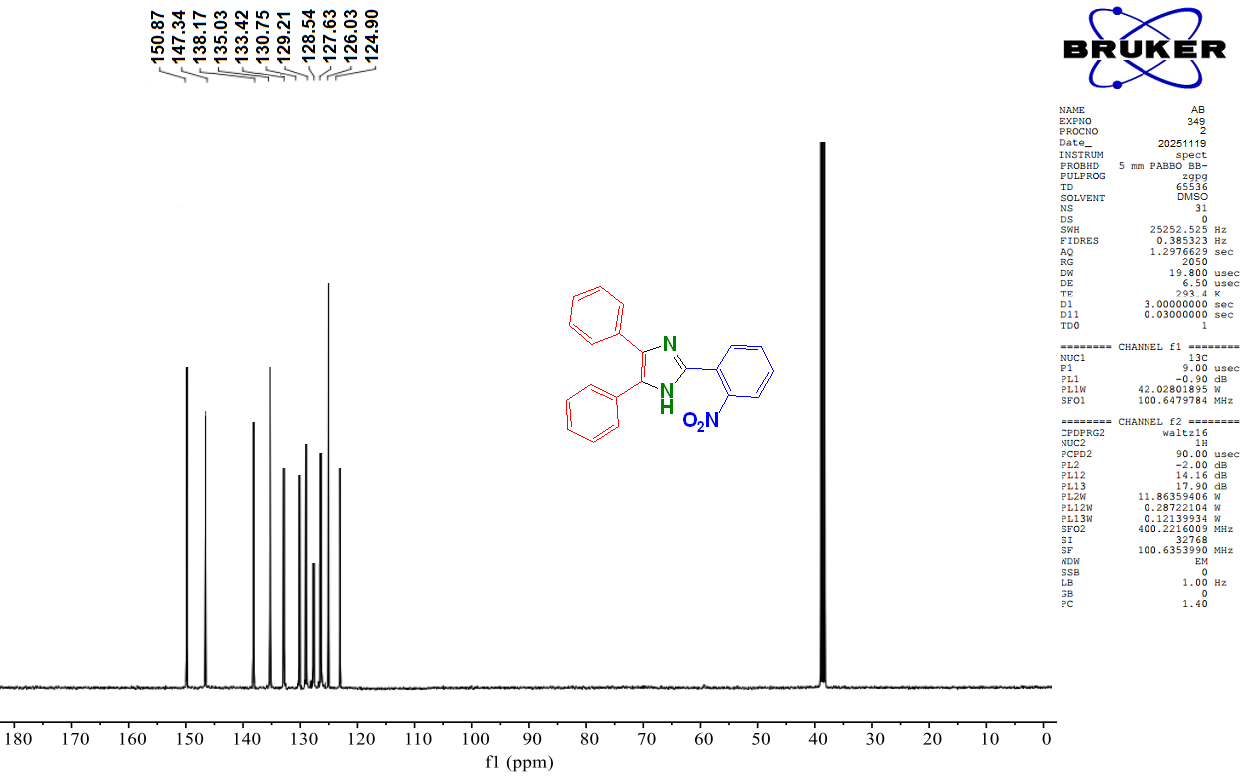


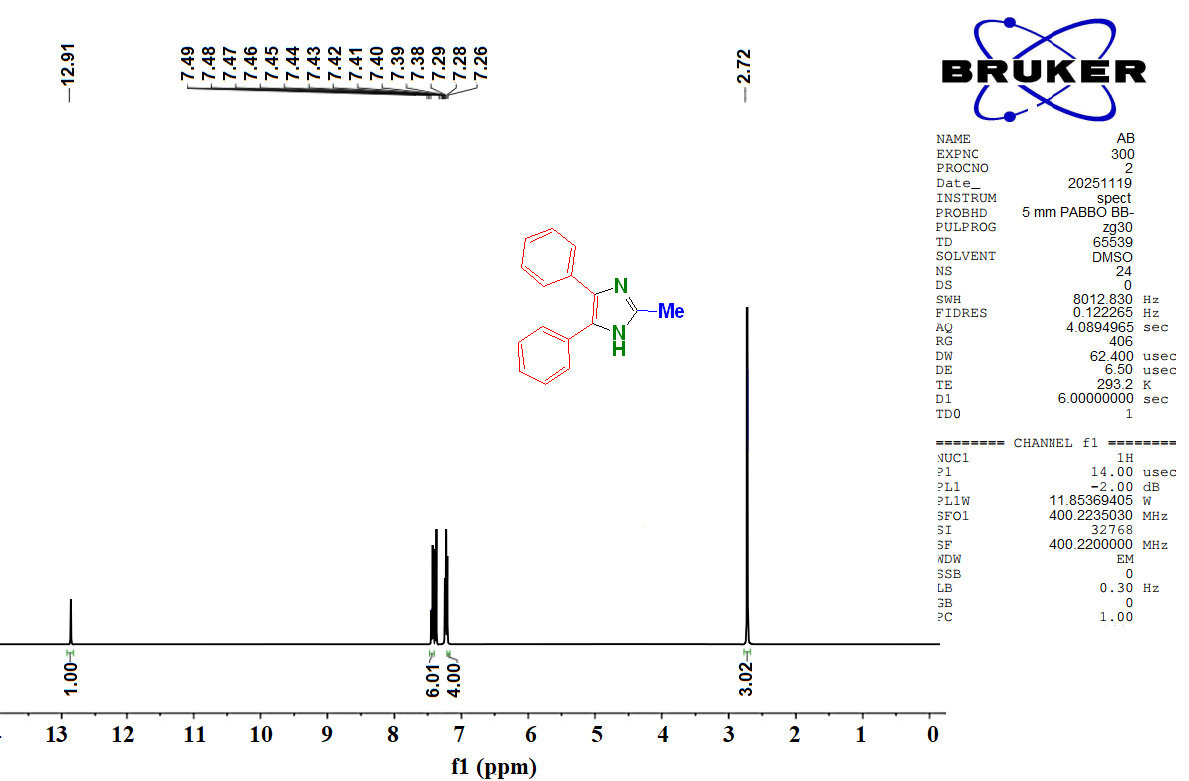


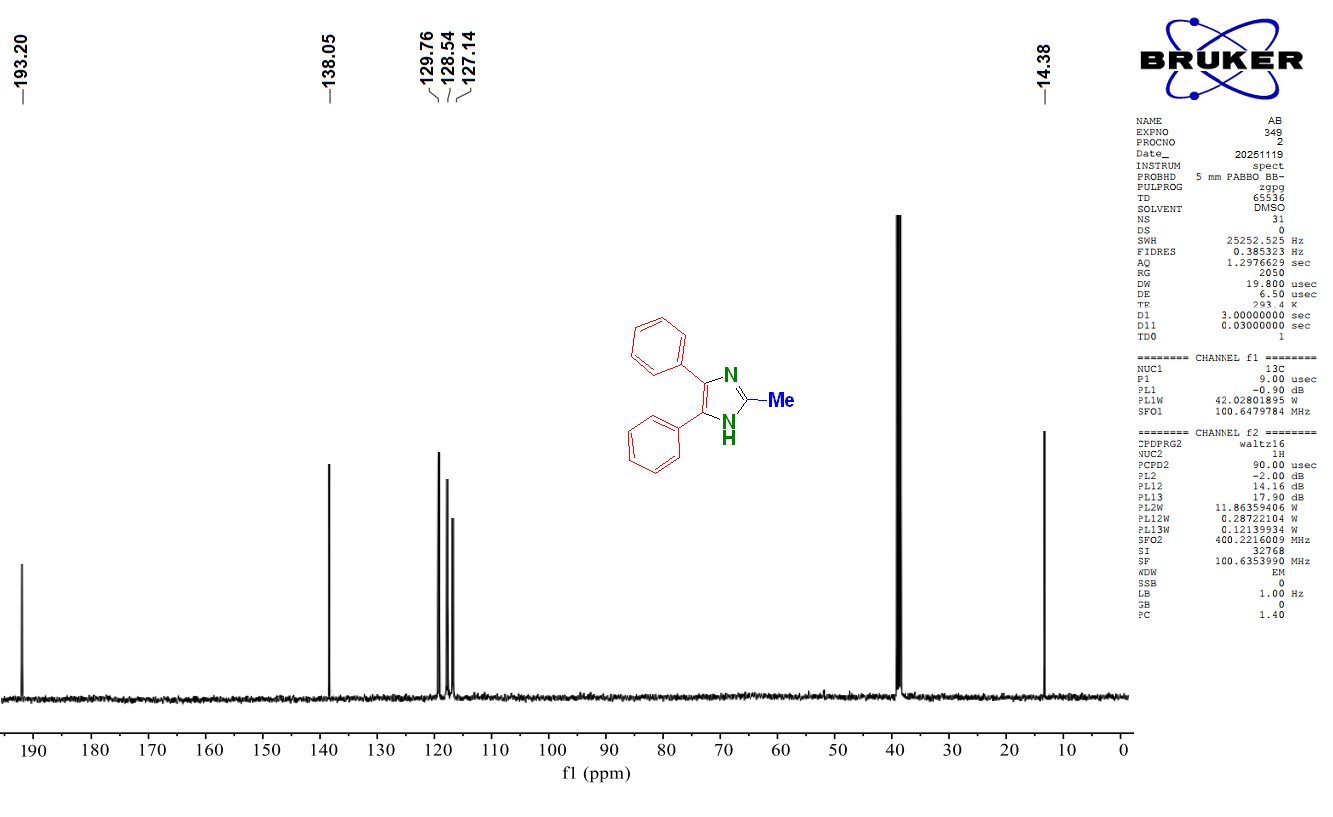


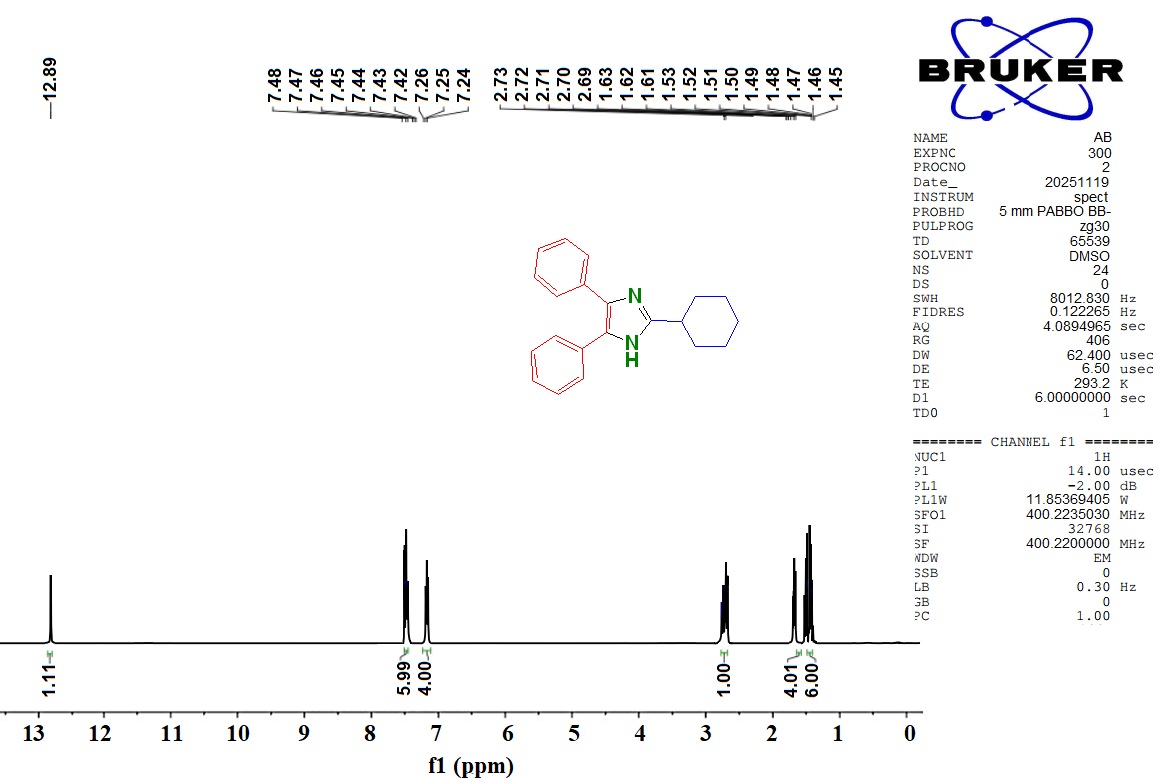


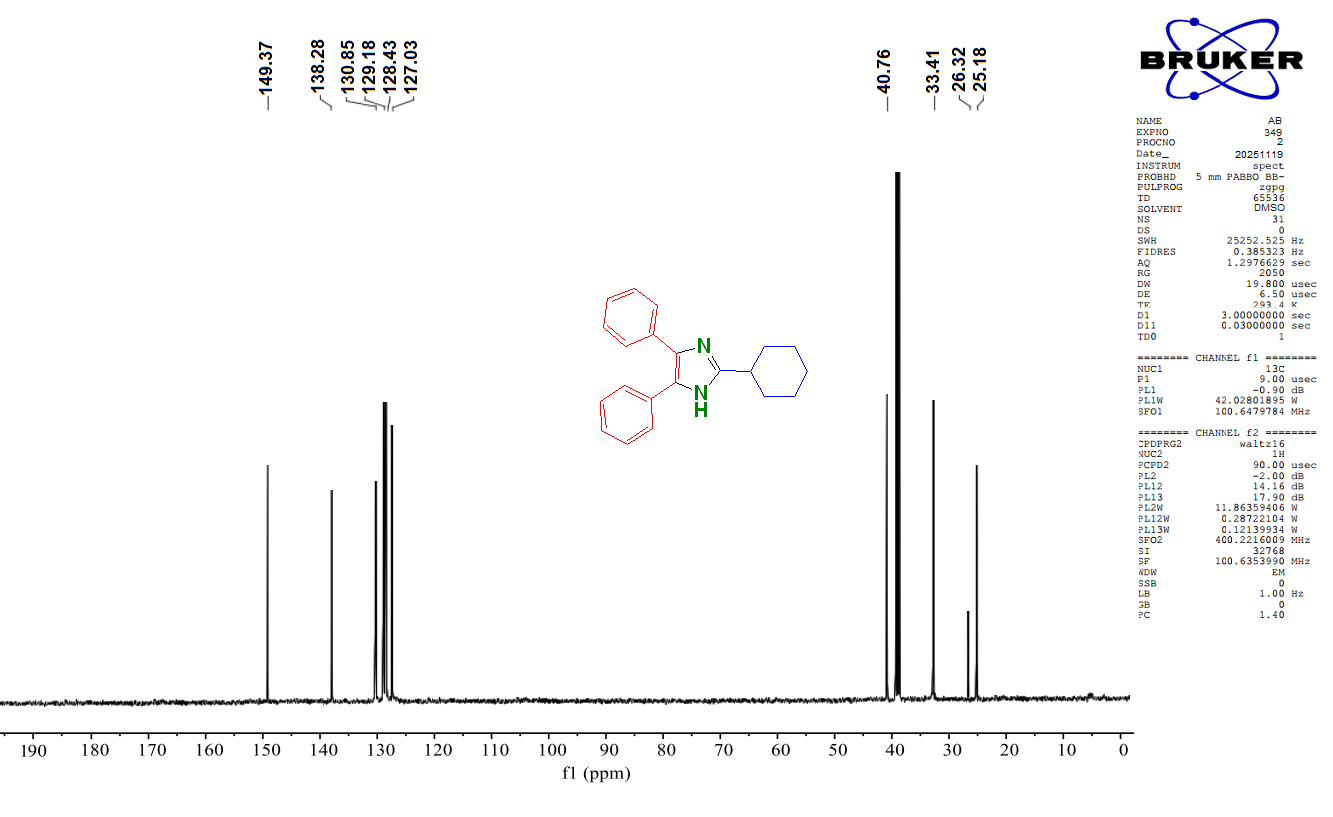


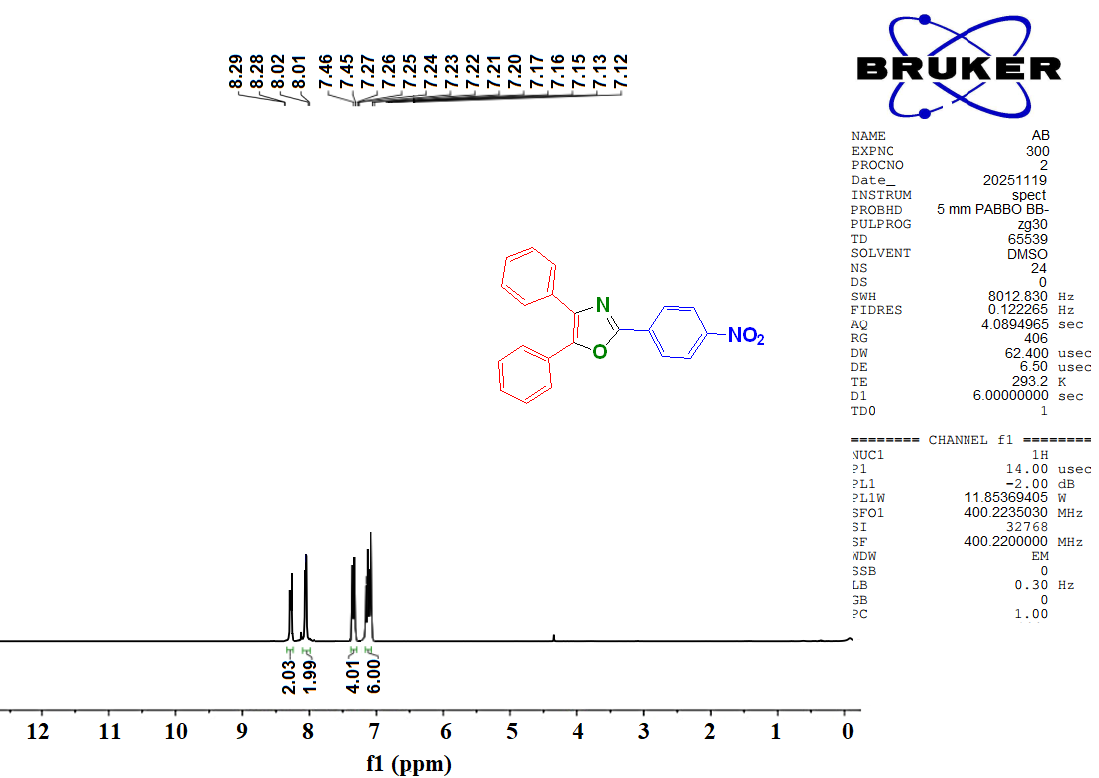


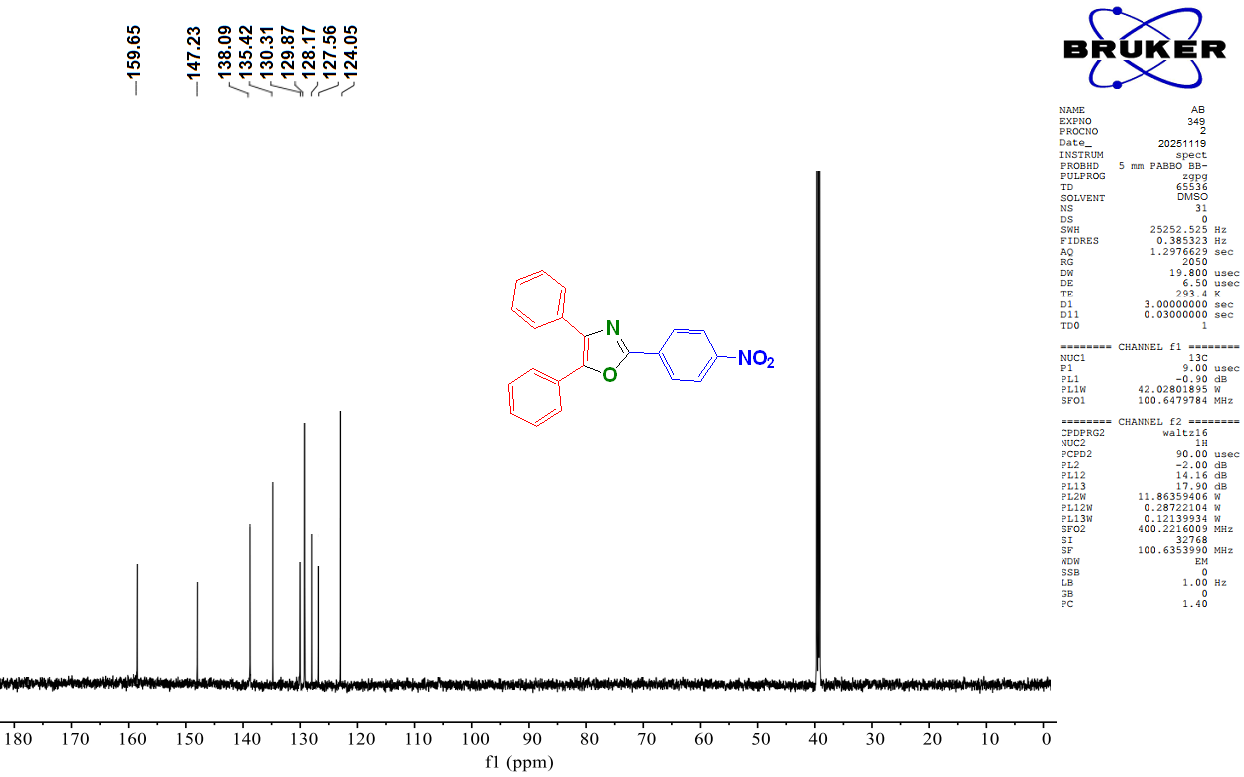

Supplement: Supplementary file 1 [file DataSheet1.docx]
